# Development of Non‐Hydrolysable Oligosaccharide Activity‐Based Inactivators for Endoglycanases: A Case Study on α‐1,6 Mannanases

**DOI:** 10.1002/chem.202101255

**Published:** 2021-05-21

**Authors:** Sybrin P. Schröder, Wendy A. Offen, Alexandra Males, Yi Jin, Casper de Boer, Jacopo Enotarpi, Laura Marino, Gijsbert A. van der Marel, Bogdan I. Florea, Jeroen D. C. Codée, Herman S. Overkleeft, Gideon J. Davies

**Affiliations:** ^1^ Leiden Institute of Chemistry Leiden University Einsteinweg 55 2333 CC Leiden The Netherlands; ^2^ Department of Chemistry, York Structural Biology Laboratory University of York Heslington York YO10 5DD UK

**Keywords:** Carbasugar, cyclophellitol, endoglycosidase, mechanism-based inhibitor, polysaccharides

## Abstract

There is a vast genomic resource for enzymes active on carbohydrates. Lagging far behind, however, are functional chemical tools for the rapid characterization of carbohydrate‐active enzymes. Activity‐based probes (ABPs) offer one chemical solution to these issues with ABPs based upon cyclophellitol epoxide and aziridine covalent and irreversible inhibitors representing a potent and widespread approach. Such inhibitors for enzymes active on polysaccharides are potentially limited by the requirement for several glycosidic bonds, themselves substrates for the enzyme targets. Here, it is shown that non‐hydrolysable trisaccharide can be synthesized and applied even to enzymes with challenging subsite requirements. It was found that incorporation of carbasugar moieties, which was accomplished by cuprate‐assisted regioselective trans‐diaxial epoxide opening of carba‐mannal synthesised for this purpose, yields inactivators that act as powerful activity‐based inhibitors for α‐1,6 *endo*‐mannanases. 3‐D structures at 1.35–1.47 Å resolutions confirm the design rationale and binding to the enzymatic nucleophile. Carbasugar oligosaccharide cyclophellitols offer a powerful new approach for the design of robust endoglycosidase inhibitors, while the synthesis procedures presented here should allow adaptation towards activity‐based endoglycosidase probes as well as configurational isosteres targeting other endoglycosidase families.

## Graphical abstract

***Carba*****‐trisaccharide cyclophellitols** are potent GH76 α‐1,6 *endo*‐mannanase inactivators and resistant to endoglycosidic bond cleavage.

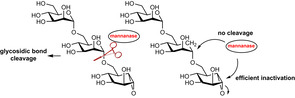

Activity‐based protein profiling is an emerging, powerful technique for the functional dissection of enzymes, often in complex milieu and frequently with major applications in human health and biotechnology.^[1]^ Consisting of three main components, a reactive (typically electrophilic) warhead, a targeting motif (largely reflecting the substrate specificity of the enzymes) in question and a reporter group (typically a fluorophore, biotin, or azide/alkyne handle for two‐step labelling). ABPs have been used across many enzyme classes. In the glycoside hydrolase/glycosidase field they have found particular use. Pioneering work by Withers trapping long‐lived covalent glycosyl‐enzyme intermediates was adapted by Vocadlo and Bertozzi to label β‐galactosidases and subsequently applied to various glycosidases.[^2^, ^3^, ^4^] Of particular note, has been the development of cyclophellitol epoxides and aziridines.[^1^, ^5^] Building upon the Madsen synthesis,^[6]^ such reagents have been used to probe numerous exoglycosidase classes, frequently those involved in lysosomal storage diseases including the Gaucher GBA β‐glucosidase,^[7]^ the Pompe α‐glucosidase,^[8]^ α‐L‐fucosidases,^[9]^ β‐glucuronidases,^[10]^ α‐L‐arabinofuranosidases,^[11]^ and others. All of these cyclophellitol‐derived inhibitors/probes were based on monosaccharide scaffolds for *“exo”* glycosidases. Very recently, the cyclophellitol concept has been extended to *endo*‐acting enzymes requiring disaccharide,^[12]^ or longer targeting/ specificity motifs ‐ compounds that proved synthetically tractable by (Lewis) acid mediated glycosylations using partially protected cyclophellitol epoxides or aziridines as the acceptor.^[13]^ Whilst this extends the potential enzyme range for glycosidase ABPs considerably, as the requirement for additional saccharide units increases, so does the potential for the ABP itself to become a substrate rendering data interpretation more complex. Indeed, recent transposition of cyclophellitol epoxides and aziridine ABPs to the biotechnology sector, exemplified by *endo*‐acting xylanases, did reveal hydrolysis of the disaccharide probes (by *exo*‐acting β‐xylosidases) to yield a functional monosaccharide ABP which could then inhibit β‐xylosidases giving rise to dual labelling of both endoxylanases and *exo*‐acting β‐xylosidases in fungal secretomes.^[12a]^ In this case, interpretation could be aided by the use of competition with exoglycosidase‐specific inhibitors, but it clearly raises the problems of using ABPs on endoglycanases generally, especially those which require inhibitors longer than disaccharides because of their complex subsite binding energies.

The CAZy (www.cazy.org) family GH76 α‐1,6 *endo*‐mannanases,^[14]^ in many ways, highlight these challenges. GH76 enzymes catalyse the hydrolysis of α‐1,6‐mannans, such as those found in the yeast cell wall, with net retention of anomeric configuration.^[15]^ In 2015 their 3D structure and putative catalytic itinerary were identified revealing ‐ in the case of the *Bacillus circulans* enzyme ‐ five enzyme subsites “‐3 to +2” (using the described nomenclature)^[17]^ with Asp124 acting as catalytic nucleophile and Asp125 as catalytic acid/base in a classic Koshland double‐displacement reaction.^[16]^ Observed −1 mannose distortion ^O^S_2_ (Michaelis complex) and B_2,5_ (*man*‐isofagomine inhibitor) conformations implied a catalytic itinerary ^O^S_2_‐[B_2,5_]^≠^ ‐^1^S_5_ for the formation of the covalent intermediate; a pathway supported by QM/MM metadynamics simulations of the reaction pathway. Notably, however, both S‐linked disaccharides and 1,6‐man‐deoxymannojirimycin bound in the −3 and −2 subsites and did not engage the catalytic apparatus hinting at very strong, indeed dominating, interactions at the −3 and −2 subsites which would challenge future inhibitor design.[^16^, ^18^] Subsequent attempts to obtain inhibitor complexes of the *B. circulans* GH76 were indeed hindered both by the domination of −3 and −2 to binding and the requirement for non‐/poorly hydrolysable inhibitors in order to occupy −3 to −1 subsites without degradation during prolonged incubations.^[18]^ This is especially important given that fungal GH76 enzymes, implicated in transglycosylation involving glycosylphosphatidylinositol anchors, are essential proteins and therefore potential broad spectrum anti‐fungal drug targets.^[19]^ We therefore thought GH76 α‐mannanases would be a challenging testbed system for oligosaccharide cyclophellitol mechanism‐based inhibitors.

Here, we report the design, synthesis and application of di‐ and trisaccharide α‐1,6‐α‐*manno*‐cyclophellitol mechanism‐based inhibitors **1**–**3** (Figure 1). We show, notably through mass spectrometry and high resolution 3‐D structural analyses, how the disaccharide cyclophellitol is indeed a poor inhibitor, but how the trisaccharide versions, in particular the carbasugar‐stabilised one, potently inhibit *B. circulans* GH76. This work now provides a strategy for the successful development and application of oligosaccharide activity‐based probes across a broad range of polysaccharide utilising enzymes with complex or stringent subsite demands.

**Figure 1:**
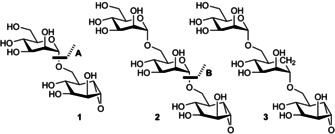
1,6‐epi‐*manno*‐cyclophellitol di‐ and trisaccharides subject of the here‐presented studies. **A** (in disaccharide **1**) denotes a possible exoglycosidase cleavage site. **B** (in trisaccharide **2**) denotes a possible endoglycosidase cleavage site, which is stabilised in **3**.

Disaccharide analogues are rarely the optimal inhibitors for endoglycosidases, but trisaccharide analogues, by virtue of their length, are necessarily also substrates lessening their potential for enzyme inhibition and application. Several strategies to make non‐hydrolysable extended inhibitors for endoglycosidases have been described in the literature. Most classically these involve the incorporation of non‐hydrolysable *S*‐ or *C*‐“glycosidic” linkages,^[20]^ although mis‐matched stereochemistry, such as in the case of the chitinase inhibitor allosamindin,^[21]^ has also been used but is totally dependent on the target enzyme accommodating but not hydrolysing the incorrect sugar and thus will not be generally applicable. In the case of GH76 α‐mannanases, not least because of the potential issues with *S*‐oligosaccharides described by Belz and colleagues,^[18]^ we chose to synthesise cyclophellitol di‐ and trisaccharides **1** and **2**, respectively, and to render the latter less hydrolysable through the incorporation of a carba sugar moiety (as in **3**). The synthesis of di‐and trisaccharides **1** and **2** follows general procedures we have previously reported for the construction of xylobiose and cellobiose cyclophellitols, and is based on the preparation of partially protected cyclitol epoxides (and aziridines) that subsequently are glycosylated using contemporary glycosylation chemistries.[^12a^, ^12b^] Details on the synthesis of compounds **1** and **2** can be found in the Supporting Information. The synthesis of carba‐trisaccharide cyclophellitol **3** necessitated the development of new methodology, which we achieved as depicted in Scheme 1.

**Scheme 1:**
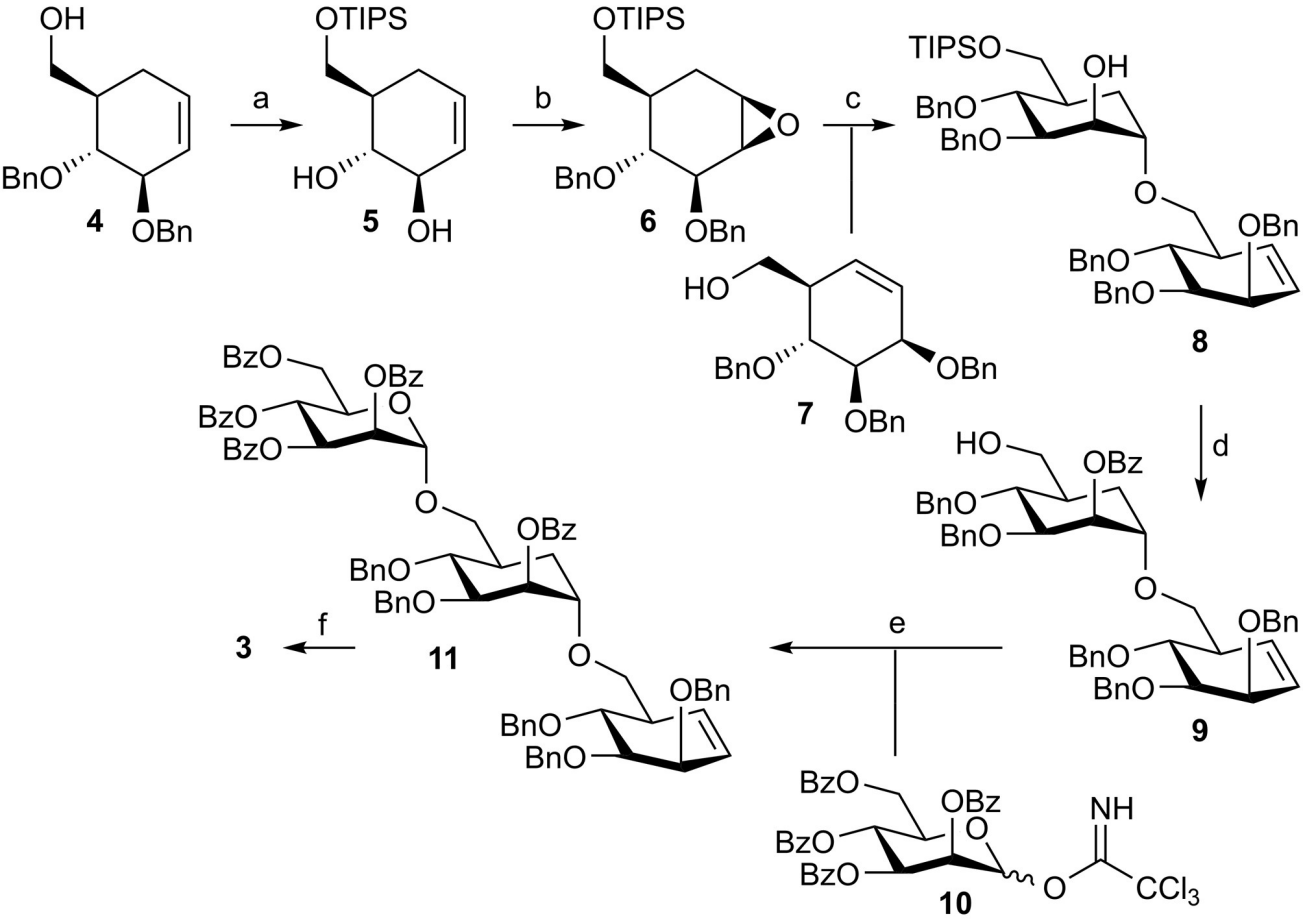
Reagents and conditions: (a) i) TIPS−Cl, imidazole, DMF, quant.; ii) Li, naphthalene, THF, −78 to −20 °C; (b) i) *m*‐CPBA, DCM, −20 °C to rt, 93 % over two steps; ii) BnBr, NaH, TBAI, THF, 0 °C to rt, 75 %; (c) acceptor **7** (2 equiv.), Cu(OTf)_2_, toluene, 40 °C, 66 %; (d) i) BzCl, pyridine, *N*‐methylimidazole, DCM; ii) TBAF, THF, 70 % over two steps; (e) donor **10**, TMSOTf, DCM, −30 °C, 1 h, 96 %; (f) i) 1,1,1‐trifluoroacetone, oxone, NaHCO_3_, EDTA, H_2_O, MeCN, EtOAc, 0 °C, 75 %; ii) NaOMe, MeOH, DCM, 83 %; iii) Pd(OH)_2_/C, H_2_, MeOH, dioxane, H_2_O, 85 %.

The key step in the synthesis is the copper(II) triflate mediated regioselective opening of orthogonally protected 1,2‐deoxy‐carbaglucose‐1,2‐epoxide **6**, for which purpose we adapted methodology recently reported by Di Bussolo and co‐workers.^[22]^ Epoxide **6** needed for this purpose was prepared as follows. TIPS protection of the primary alcohol in protected carba‐glucal **4** and ensuing Birch debenzylation gave cyclohexene **5**.^[23]^ Treatment of **5** with mCPBA in dichloromethane led to stereoselective epoxidation as directed by the allylic alcohol, and ensuing benzylation of the two secondary alcohols gave epoxide **6** in good yield. Regioselective, trans‐diaxial nucleophilic opening of the epoxide in **6** with the primary alcohol of partially protected *manno*‐cyclohexene **7** (see for the synthesis of **7** the Supporting Information) was effected under the agency of a catalytic amount of Cu(OTf)_2_ under slightly elevated temperature,^[22]^ affording **8** in good yield and as the single regioisomer observed. Protective group manipulations then gave **9**, which was glycosylated with mannosyl trichloroacetimidate **10** (Scheme 1) to yield pseudo‐trisaccharide **11** in high yield. Epoxidation of the alkene in **11** using in situ generated 1,1,1‐trifluoro‐dimethyldioxirane proceeded with good yield and stereoselectivity. Global two‐step deprotection of both benzoyl esters and benzyl ethers finally gave target trisaccharide cyclophellitol **3**.

Armed with the 6‐epi‐*manno*‐cyclophellitol di‐ and trisaccharides **1**–**3**, we next sought to study the inhibition and mode of binding of these compounds in crystal structures of *Bacillus circulans* GH76 (*Bc*GH76).^[16]^ We used a more easily crystallizable variant, R341Q, designed to prevent formation of an intermolecular salt bridge due to crystal packing and allow ligand complex formation, but which is otherwise unchanged in catalytic machinery and efficiency.^[16]^ Details of X‐ray structures are given in Table 1 and Supporting Information Table S1.

**Table 1:** Details of *Bc*GH76 complexes with 6‐epi‐*manno*‐cyclophellitol di‐ and trisaccharides **1**–**3**. Full details in Table S1.

	D125 N (**2**)	D125 N (**3**)	WT^[a]^ (**3**)
Resolution	1.40 Å	1.47 Å	1.35 Å
PDB Code	6ZBW	6ZBM	6ZBX

[a] WT and D125 N crystals refer to an R341Q variant (with WT active site residues) and D125 N/R341Q respectively.

Initial attempts to soak the α‐1,6‐α‐*manno*‐cyclophellitol disaccharide **1** into crystals of a crystallization variant, R341Q, of the “wildtype” enzyme (R341Q variant) yielded a complex with density showing only the −3/‐2 subsites occupied (not shown, but similar to that seen in the −3/‐2 sites for the trisaccharide complexes below). A soak with the trisaccharide **2** revealed a mixture of partially occupied trisaccharides and hydrolysed disaccharide product, with the latter again occupying subsites −3 and −2 (Supporting Information Figure S1). The refined structure (whose interpretation was rendered far more facile when subsequently viewed in Iight of the complexes D125 N with **2** and **3**, described below) showed low, partial, occupancy by a covalently bound sugar in the −1 subsite with the C1 position refining to approximately 1.55 Å from the OD2 carboxylate oxygen of the nucleophile Asp 124. Further examination of the electron density map calculated after modelling the covalent species revealed the additional presence of an unreacted epoxide in the −1 subsite (Supporting Information Figure S1). Given the clarity of density for an α‐1,6 mannobiose in the −3 and −2 subsites, which was sufficient to allow refinement of fully occupied disaccharide in these sites, the presence of mixed di‐ and partial trisaccharide species clearly highlights the challenges of the unmodified trisaccharide inhibitor on this system.

In order to test our hypothesis about partial occupancy and ligand hydrolysis, we harnessed the D125 N variant of *Bc*GH76. This is a variant of the catalytic acid/base which is inactive against unactivated glycosides, but which we reasoned would react with the 6‐epi‐*manno*‐cyclophellitol epoxides, even if at a reduced rate. Indeed, rapid (30 mins) soaking of **2** into these D125 N crystals led to a partially occupied “disaccharide” complex (density only really clear in −3 an −2, not shown), but a 10‐day soak (1 mM **2**) produced a full occupancy covalent adduct to the catalytic nucleophile Asp124, Figure 2a. This complex shows that fully‐reacted covalent adduct may be formed when the enzyme is depleted in catalytic activity.

**Figure 2:**
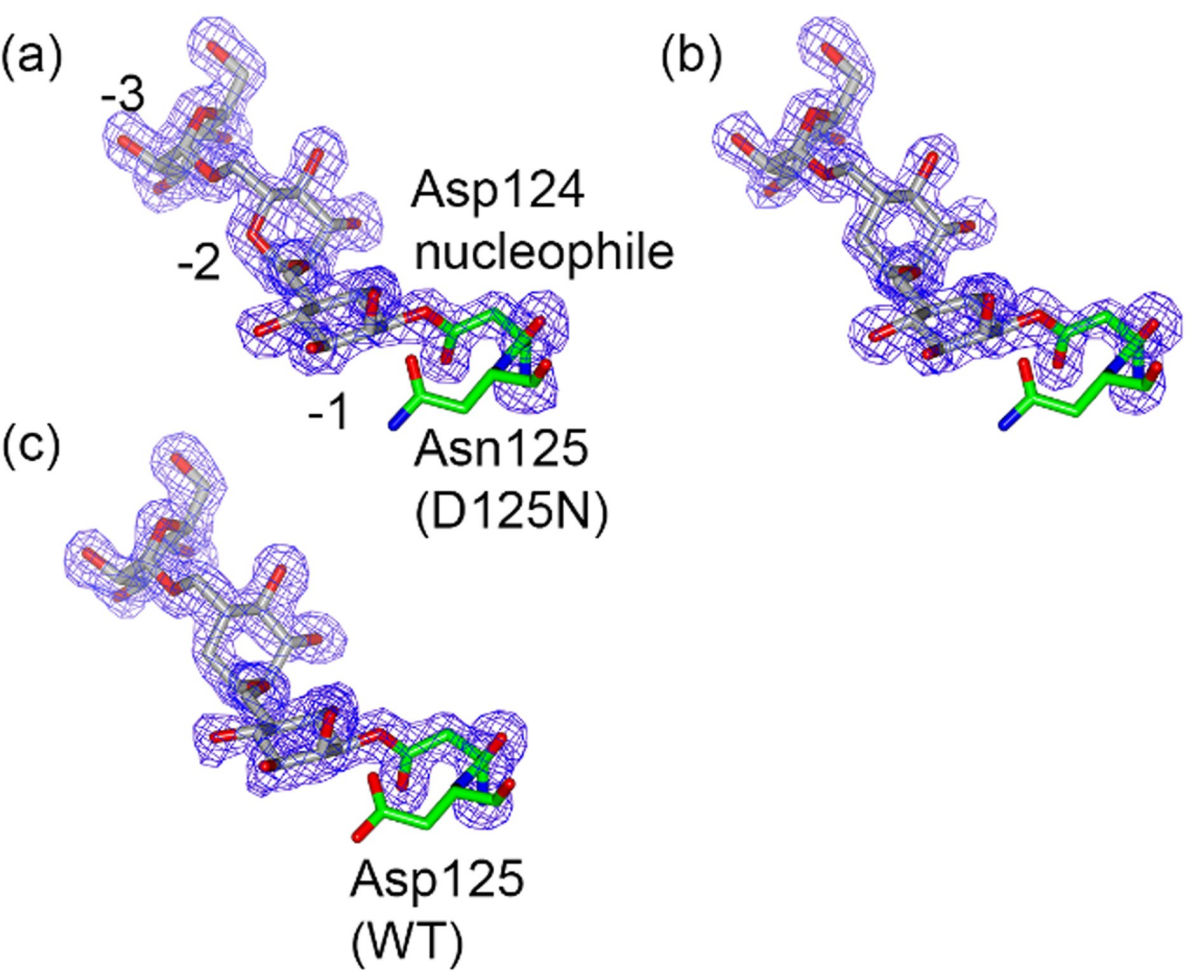
Fo−Fc “omit” electron density for the 6‐epi‐*manno*‐cyclophellitol “trisaccharide” **2** and **3** bound to *Bc*GH76. (a) **2** bound to D125 N variant, (b) **3** bound to 125 N (c) carbacycle‐containing “trisaccharide” **3** bound to the WT enzyme. Contour levels are 3 r.m.s.d. (0.46, 0.52, and 0.45 e^−^ Å^−3^, respectively).

The trisaccharide binds in the −3 to −1 subsites, with identical interactions to previous GH76 complexes in −3 and −2. In the −1 subsite, the 6‐epi‐*manno*‐cyclophellitol has opened (or is at least subsequently observed bound) trans‐equatorially (as opposed to the more common transdiaxial opening), Figure 2. The mannose ring is observed in ^4^C_1_ chair conformation ‐ one inconsistent with the proposed conformational pathway (^O^S_2_‐[B_2,5_]^≠^‐an intermediate initially in ^1^S_5_ conformation)_._ Comparison of the covalent adduct here, with the ^O^S_2_ Michaelis complex (PDB 5AGD),^[16]^ shows that interactions at the −3, and −2 subsites are identical. However, in the −1 subsite, the pseudo‐sugar ring of the *manno*‐cyclophellitol is displaced from its position in the Michaelis complex with its ring plane rotated greatly (beyond perpendicular) to that seen in the natural substrate, to such an extent that the oxygens equivalent to O3 and O4 actually lie 5–6 Å from their positions observed for the Michaelis complex in a position that would not be possible for a natural substrate (Supporting Information Figure S2). Whilst other trapped cyclophellitols we have observed appear in conformations and positions consistent with the known itineraries of the enzymes, this is not the case here.

Having shown that a trisaccharide inhibitor would bind successfully when the enzyme was inactive for normal hydrolysis, we then sought to study the binding of the trisaccharide **3**, which we postulated would be non‐hydrolysable by virtue of containing a central carbacycle, to both WT and, as a control, D125 N enzymes. Compound **3** indeed bound to D125 N in an identical manner to **2**, Figure 2B. Crucially, in marked contrast to **2**, on catalytically active WT enzyme the bespoke carbacycle‐containing “trisaccharide” **3** covalently bound to the WT enzyme at full occupancy, Figure 2C, confirming that non‐hydrolysable inhibitors for complex *endo*‐active enzymes can be designed successfully.

Compounds **2** and **3** both make identical interactions to *Bc*GH76 (see Figure 3 for that of **3** and the Supporting Information for **2**). As with reported complexes involving competitive inhibitors, interactions in the −3 and −2 subsites are mainly via hydrogen bonds with Trp172, Asn181, Asp228, Arg229, Asp239, and Tyr243. As described above, the −1 sugar is in ^4^C_1_ chair conformation with the sugar flipped out of the active centre and making none of the interactions seen for the Michaelis complex (5AGD) or the previously reported *man*‐isofagomine complex (PDB 4D4D).

**Figure 3:**
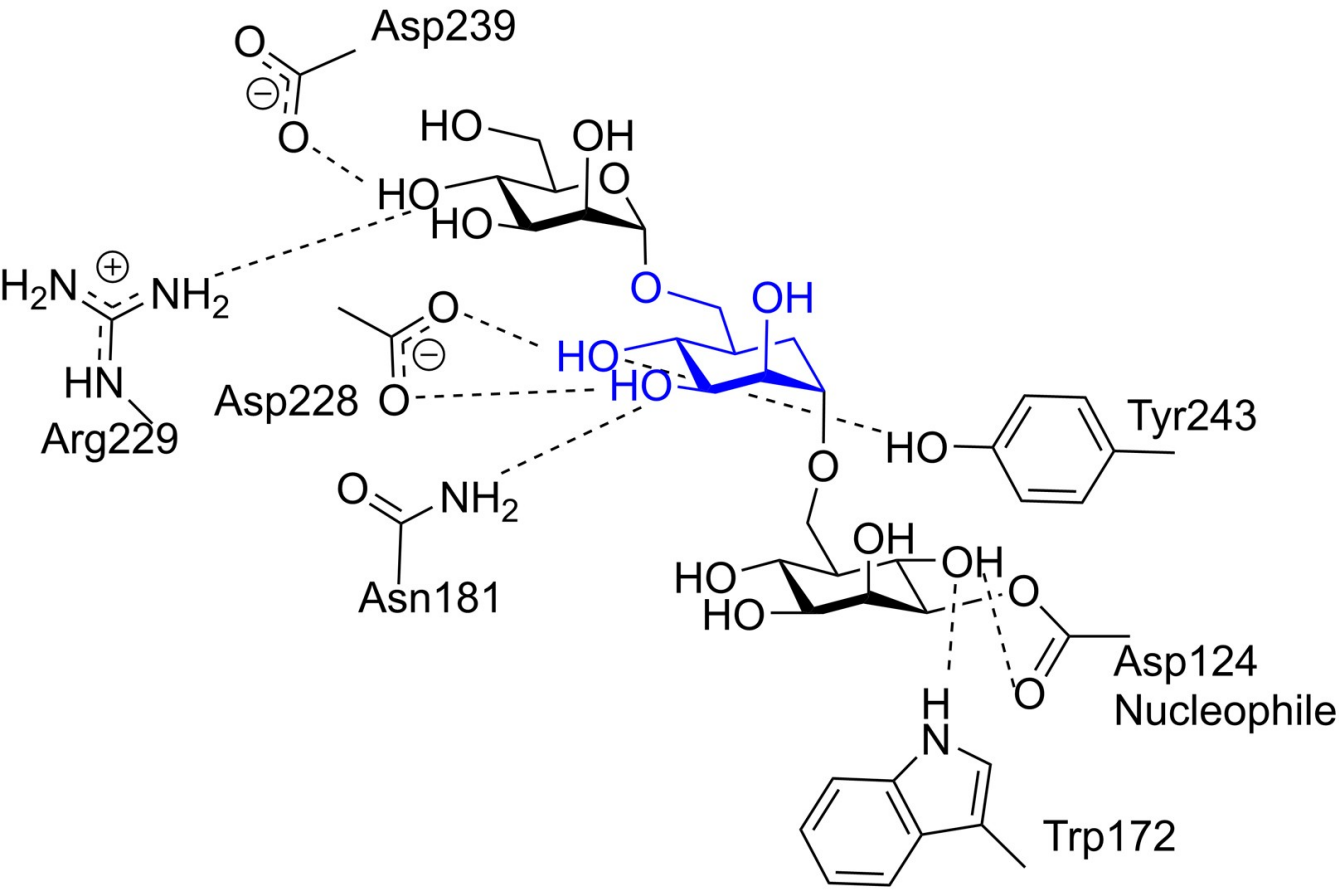
Interactions of the carbacycle (blue) within the 6‐epi‐*manno*‐cyclophellitol “trisaccharide” **3** with WT *Bc*GH76.

Although crystal structures are clearly indicative that the carbacycle containing inhibitor **3** reacted, as expected, with the wild‐type enzyme, and that **2** only reacts well with the D125 N variant, we sought to verify these structural observations through mass spectrometry in solution. We therefore analysed the molecular weights and relative weight distribution of *Bc*GH76 proteins incubated with probes **1**–**3**. To this end, two non‐variant WT and mutant construct versions (WT‐*Bc*GH76, *Bc*GH76‐D125 N) were used, as well as the two R341Q variants used for the above structural studies (“WT” *Bc*GH76‐R341Q and the double mutant, *Bc*GH76‐D125 N/R341Q) with each of the three probes at a probe‐protein concentration ratio of 10 : 1 in 25 mM HEPES buffer at pH 7 with 100 mM NaCl for 16 to 22 h at 293 K. The results are depicted in Table 2 with the numbers indicating the relative amount of protein that has reacted with the respective probe (total protein count 100 %).

**Table 2:** Percentage of reacted mannanases with the respective probes after 16 or 22 h of incubation. Both wild‐type and non‐catalytic mutant (R341Q) are fully transformed into the covalent enzyme‐inhibitor adduct with stabilised trisaccharide **3**, but not with inhibitors **1** and **2**.

Protein	16 h incubation			22 h incubation		
	**1**	**2**	**3**	**1**	**2**	**3**
Wild Type	25	30	100	30	30	100
D125 N	0	75	40	0	85	44
R341Q	25	17	100	30	17	100
D125 N/R341Q	0	75	40	0	85	45

These data confirm results from the crystallisation studies that carba‐trisaccharide **3** is the most effective and ‘clean’ inhibitor of the set. Both the wild‐type enzyme (*Bc*GH76) and non‐catalytic mutant (*Bc*GH76‐R341Q) reacted to form the enzyme‐probe adduct with 100 % efficiency. Both disaccharide **1** and trisaccharide **2** react much less efficiently, with maximally 30 % conversion to the enzyme‐probe adduct for both these enzymes. These results strongly support our initial hypothesis that, where disaccharide inhibitors are too small to optimally occupy multiple substrate binding sites, non‐stabilized trisaccharide inhibitors may fall prey to enzymatic processing to yield non‐inhibitory products.

From the experiments incubating the two catalytic acid/base mutants, *Bc*GH76‐D125 N and *Bc*GH76‐D125 N/R341Q, disaccharide probe **1** proved unreactive likely reflecting impaired binding to the variant. Probes **2** and **3**, likely because of their enhanced active site affinity, do react to yield covalent adducts, data supporting the co‐crystal structure obtained from the complex of **3** and *Bc*GH76‐D125 N. The difference in the efficiency of opening the epoxide in probes **2** and **3** by the catalytic nucleophile (D124) in the mutant enzymes, as opposed to the wild‐type, is likely caused by the absence of the catalytic acid‐base. We have previously found that mechanism‐based retaining exoglycosidase inhibitors may still react with mutant enzymes with the catalytic acid‐base substituted, even while such enzymes are catalytically inactive and ascribe this phenomenon to the enhanced intrinsic reactivity of an epoxide (or related electrophile) compared to an interglycosidic acetal linkage. This reasoning may in fact also explain the at a first glance counterintuitive result that inhibitor **2** reacts more efficiently with the acid‐base variants than **3** ‐ the opposite result from the experiments with the two wild‐type (with respect to the active site) enzymes. Arguably, the active site mutants are not able to hydrolyse the glycosidic linkage connecting the *manno*‐disaccharide in **2** with the *manno*‐cyclophellitol. In this scenario, which supports our design, nucleophilic opening of the epoxide remains as the only option for the mutant enzymes, whereas the wild‐type ones have an alternative pathway in processing the endoglycosidic linkage.

In summary, we have described the rational design of an effective endomannosidase trisaccharide inhibitor composed of two carbasugars: one bearing an epoxide at the reducing end that acts as the electrophile for covalent, irreversible inhibition and one taking up position of an internal mannosides to protect the construct from endomannosidase mediated hydrolysis. For this purpose, we developed a concise and effective route of synthesis that moreover is flexible for adaptation towards analogous tri‐and oligosaccharidic structures. Variation of substitution pattern and configuration of electrophile **6** and nucleophile **7** in Scheme 1 should allow for the construction of inhibitors targeting other retaining endoglycosidase families, whereas endowing either the reducing end sugar analogue (by replacing the epoxide oxygen with a substituted aziridine nitrogen) or the non‐reducing end sugar with a reporter entity (biotin, fluorophore) will open up activity‐based endoglycosidase profiling studies. Our results thus prove our working hypothesis to bear out: effective mechanism‐based endoglycosidase inhibitors can be designed taking into account stabilization of internal glycosidic linkages through the employment of carbacyclic entities. These results underscore literature findings on competitive endoglycosidase inhibitors and our design and synthetic methodologies may also find application in the design of such entities. Our own research focuses on the design of inhibitors and activity‐based probes for a range of endoglycosidase families and chemistries and designs, as reported here, will be implemented in these studies.

## Conflict of interest

The authors declare no conflict of interest.

## Supporting information

As a service to our authors and readers, this journal provides supporting information supplied by the authors. Such materials are peer reviewed and may be re‐organized for online delivery, but are not copy‐edited or typeset. Technical support issues arising from supporting information (other than missing files) should be addressed to the authors.

Supplementary
