# Supplementary material for: Development of Non‐Hydrolysable Oligosaccharide Activity‐Based Inactivators for Endoglycanases: A Case Study on α‐1,6 Mannanases
Source: Chemistry. 2021 May 21;27(37):9519–23. doi: 10.1002/chem.202101255 (PMC8362039; doi:10.1002/chem.202101255)
Supplement: Supplementary file 1 — Supplementary [file CHEM-27-9519-s001.pdf]

Chemistry–A European Journal

Supporting Information

Development of Non-Hydrolysable Oligosaccharide Activity-Based Inactivators for Endoglycanases: A Case Study on α-1,6 Mannanases

Sybrin P. Schröder[+], Wendy A. Offen[+], Alexandra Males, Yi Jin, Casper de Boer, Jacopo Enotarpi, Laura Marino, Gijsbert A. van der Marel, Bogdan I. Florea, Jeroen D. C. Codée, Herman S. Overkleeft,* and Gideon J. Davies*

Contents

1. Protein Purification, Crystallization, Data Collection and Refinement 2
2. Mass spectrometry analysis 5
3. Synthetic procedures 6
   General methods 6
   Synthesis of inhibitors **1** and **2** 7
   Synthesis of inhibitor **3** 10
4. NMR spectra 14
5. References 32

1. Protein Purification, Crystallization, Data Collection and Refinement

The genes encoding wildtype *Bc*GH76 (surface mutant R341Q) and acid/base mutant D125N thereof were overexpressed and the proteins purified as in reference [1]. Solutions of the wildtype enzyme (at 40 mg/ml in 25 mM HEPES pH 7.0, 100 mM NaCl, 1 mM dithiothreitol and 20 mg/ml in 10 mM HEPES, 39 mM NaCl, 61 mM Bis Tris pH 6.5) were used to grow crystals for soaking with trisaccharide cyclophellitol (**2**) and the carba-trisaccharide cyclophellitol (**3**) respectively, and of the D125N variant (at 20 mg/ml in 4 mM HEPES pH 7.0, 17 mM NaCl, 100 mM Bis Tris pH 6.5), to grow crystals for soaking with both ligands. Sitting drops were set up in ratios of 0.25-0.5:0.1:0.4 for the protein:seed:reservoir solutions, over 20 % (w/v) polyethylene glycol (PEG) 3350, 0.1 M ammonium nitrate pH 6.5 for the WT crystals soaked with **2**, and 22 % (w/v) PEG 3350, 0.1 M ammonium nitrate (not pHed) for the other crystals. The seed stock was produced by vortexing crystals in reservoir solution in the presence of a polystyrene bead, using crystals obtained after several similar rounds of seeding, from protein crystallized at 30 – 40 mg/ml over 24-26% (w/v) PEG 3350, 0.1 M ammonium nitrate, pH 6.5.

The wildtype crystals were soaked with 10 mM **2** for 30 min, or 6.7 mM **3** for 2 days, and the D125N crystals were soaked with either 1 mM **2** for 10 days, or 10 mM **3** for 5 days, before fishing by step transfer through reservoir solutions supplemented with 20 % (w/v) PEG 550 monomethyl ether (for WT-**2**) or 20 % (w/v) ethylene glycol (for WT-**3** and the D125N crystals) as a cryoprotectant (in 5% increments up to 20%) and freezing in liquid nitrogen.

Data were collected on beamline I04 at the Diamond Light Source, and were processed using DIALS for the WT-**2** and both ligand **33** datasets and XDS for the D125N-**2** data,[2,3] and reduced and analysed in the CCP4i2 pipeline using POINTLESS, AIMLESS and CTRUNCATE.[4] The data for wildtype BcGH76 with **2** were in space group $P2_{1}$„ with unit cell dimensions 43.5, 65.4, 49.4 Å, 90 ˚, 101.9, 90˚, and 1 molecule per asymmetric unit, and for WT- **3** were in space group *P*1 with unit cell dimensions 47.4, 51.1, 66.2 Å, 93.6, 92.4, 98.2˚, and 2 molecules per asymmetric unit. The datasets for the D125N mutant complexes were in space group $P2_1$, with unit cell dimensions 43.9, 66.4, 98.9 Å, 90˚, 100.1, 90˚, and 2 molecules per asymmetric unit for the ligand **2** soak, and 43.8, 66.1, 49.4 Å, 90, 100.2, 90˚, with 1 molecule per asymmetric unit, for the ligand **3** soak. The structures were solved by molecular replacement with MOLREP using protein chain A of PDB entry 4D4A as the model.[5] The structures were refined using cycles of maximum-likelihood refinement with REFMAC interspersed with manual rebuilding in COOT.[6,7]

	*Bc*GH76[a]-**2**	*Bc*GH76- D125N-**2**	*Bc*GH76-**3**	*Bc*GH76- D125N-**3**
Data collection				
space group	*P21*	*P21*	*P1*	*P21*
a, *b*, *c* (Å)	43.5, 65.4, 49.4	43.9, 66.4, 98.9	47.4, 51.1, 66.2	43.8, 66.1, 49.4
α, β, γ (deg)	90.0, 101.9, 90.0	90.0, 100.1, 90.0	93.6, 92.4, 98.2	90.0, 100.2, 90.0
resolution (Å)	48.43-1.40 (1.42-1.40)[b]	54.87-1.40 (1.42-1.40)	50.44-1.35 (1.37-1.35)	66.12-1.47 (1.50-1.47)
R_{sym} or R_{merge}	0.092(0.511)	0.044 (1.023)	0.035 (0.504)	0.186 (0.994)
R_{pim}	0.054(0.374)	0.029 (0.629)	0.026 (0.398)	0.130 (0.631)
$CC_{1/2}$	0.993(0.742)	0.998 (0.633)	0.997 (0.638)	0.950 (0.676)
$I/\sigma I$	6.4(1.6)	6.7 (0.9)	14.1 (1.7)	3.1 (1.1)
completeness (%)	99.4(90.2)	99.4 (98.6)	95.1 (88.5)	99.8 (99.7)
redundancy	3.8(2.6)	4.2 (4.2)	3.5 (3.0)	4.0 (3.4)
Refinement				
no. reflections	53025	103667	121581	44764
R_{work}/R_{free}	0.15/0.17	0.15/0.21	0.13/0.18	0.16/0.22
no. atoms				
protein	2701	5339	5456	2685
ligand/ion	46	100	76	42
water	295	439	584	178
B-factors (Å^2)				
protein	17.4	20.9	17.8	15.2
ligand/ion	17.8	21.2	19.7	13.7
water	28.9	30.6	29.6	23.7
r.m.s. deviations				
bond lengths (Å)	0.012	0.013	0.014	0.012
bond angles (deg)	1.831	1.772	1.867	1.722
Ramachandran plot residues				
in most favorable regions (%)	99.4	99.4	99.1	99.1
in allowed regions (%)	0.6	0.6	0.6	0.9
PDB code	7NL5	6ZBW	6ZBX	6ZBM

[a] *Bc*GH76 refers to R341Q crystallization variant catalytic domain of *Bc*GH76 for all structures shown.
[b] Number in parentheses is value for highest resolution shell.

WT-*Bc*GH76-2 Structure

A structure of *Bc*GH76 WT with trisaccharide cyclophellitol **2** exhibited partial density in the -1 subsite. The active site was initially modelled with -3/-2 subsite mannoses fully occupied, and with the -1 site partially occupied with a covalently bound form of the epoxide, based on the omit f_o-f_c density observed after the protein and water molecules had been refined (and Asp124 omitted), and before any ligand had been added (Supplemental Figure 2A). The F_o-F_c electron density calculated after adding the ligand and Asp124 revealed further difference density for an unreacted epoxide, which was also able to be modelled at an occupancy of 0.4 (see Supplemental Figure S1B). Therefore, the active site was modelled with 2 molecules of trisaccharide **2**, one covalently bound, and the other unreacted, each at an occupancy of 0.4, and with an additional mannobiose in -3/-2 at an occupancy of 0.2.

A B

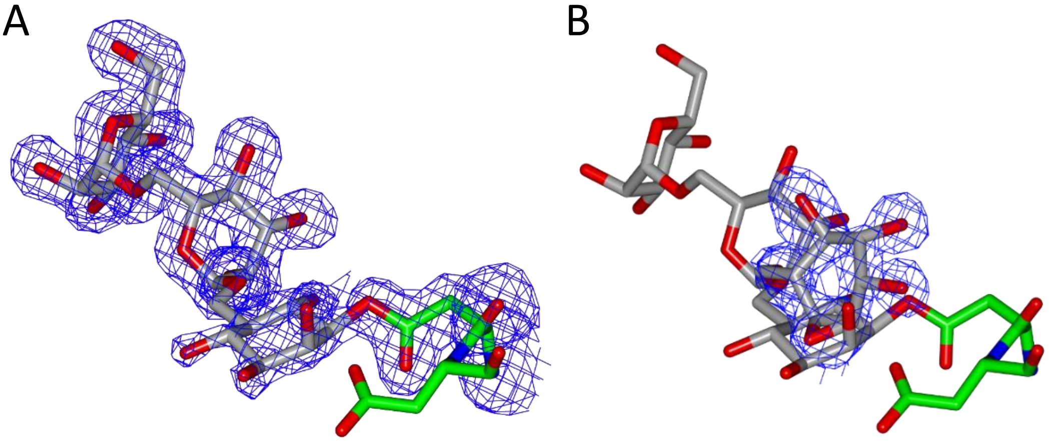

Supplemental Figure S1. **A.** Electron density map for the structure of WT-*Bc*GH76-**2.** Active site residues and partially modelled covalently bound trisaccharide 2 are shown, with C atoms in green and grey respectively. The electron density mesh is a REFMAC maximum-likelihood/σ A -weighted Fo-Fc omit map, calculated without the ligand and catalytic nucleophile Asp124, contoured at 2 r.m.s.d., 0.293 e/Å^3. **B.** The f_o-f_c difference density calculated after mannobiose modelled at full occupancy in -3 and -2 subsites, and covalently bound epoxide at occupancy 0.4 in the -1 subsite, revealing electron density for unreacted epoxide (contour level 2.5 r.m.s.d., 0.333 e/Å^3). Figure made using CCP4mg.[8]

Interestingly, the unreacted trisaccharide lies in a similar position to the -3 to -1 subsite sugars of α-1,6-mannopentaose (in 5AGD.PDB), whilst the reacted sugar has been flipped approximately 90 degrees, and is oriented in a non-canonical conformation, shown in overlay Supplemental Figure S2A. The epoxide interacts with similar side-chains to the Michaelis complex mannose, involving the 3'OH and Trp128, the 4'OH and Tyr243, and from the 2'OH to Asn125 or Asp125 (in 5AGD.PDB and WT-*Bc*GH76-**2** respectively) (see Supplemental Figure S2B). The covalently bound *manno*-cyclophellitol occupies a flipped position forming a hydrogen bond between the newly formed hydroxyl group and Trp172. The 2'OH also forms a hydrogen bond to Asp125, whilst the 3' and 4'OH are oriented in a different direction, with the latter exhibiting a hydrogen bond to Asp294. Whilst the unreacted epoxide lies roughly parallel to the side-chain of Trp73, the covalent sugar forms an aromatic interaction with the side-chain of Phe122 on the other side of the -1 pocket.

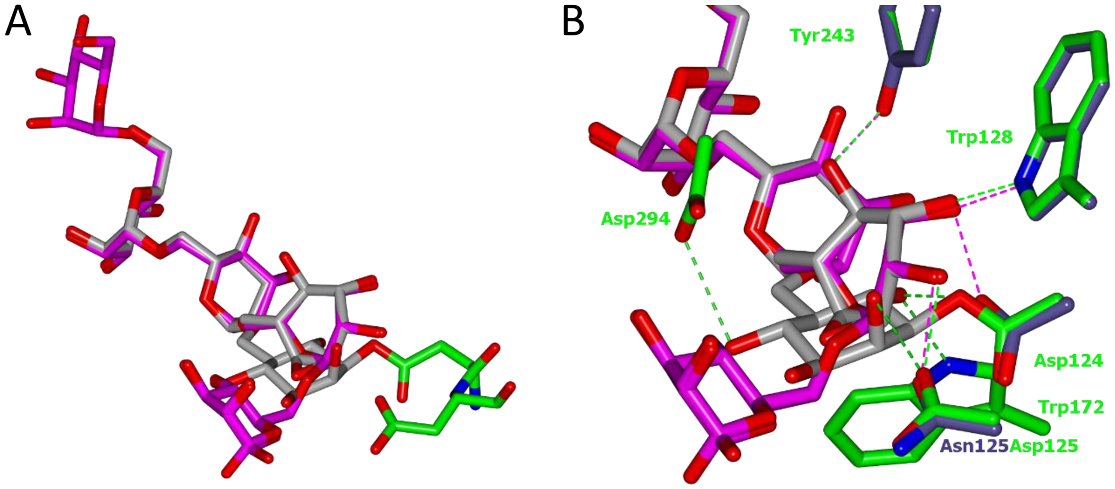

Supplemental Figure S2. **A.** Ligands in overlay of the WT-*Bc*GH76-**2** structure (ligand in grey, catalytic nucleophile and acid base side-chains in green) with the structure with α-1,6-mannopentaose (5AGD.PDB) (magenta). **B.** Hydrogen bonding interactions between the minus 1 subsite sugars and neighboring side-chains (hydrogen bonds shown as dashed lines in green for both covalent and unreacted ligands of WT-BcGH76-**2**, and in magenta for 5AGD.PDB, and with side chains in green and dark slate blue respectively).

2. Mass spectrometry analysis

Proteins were diluted to 10 µM, probes were added as 10-fold excess (100 µM end concentration) in 50 µl reaction buffer (25 mM HEPES pH 7, 100 mM NaCl) and reacted for 16 and 22 hr at 293 K with mild shaking. Samples were buffer exchanged to 10 mM ammonium acetate using P6 columns containing acrylamide-based beads of 6 kDa cut-off (Bio-Rad) and kept at 4 °C. For LC-MS analysis, 1 µl sample (10 pmole protein) was injected on a nanoAcquity UPLC system and sprayed under mild electro spray ionization (ESI) into a Synapt G2Si time of flight mass spectrometer (Waters), followed by 3 blanc injections to remove carry over effects. For the reversed phase analysis, the stationary phase was an Acquity UPLC M-Class 300 µm x 50 mm column, packed with BEH C4 material of 1.7 µm diameter and 300 Å pore size particles (Waters). The mobile phase A, 0.1% formic acid in ultrapure water and mobile phase B, 0.1% formic acid in acetonitrile were of ULC/MS grade quality from Biosolve (Valkenswaard, the Netherlands) or Sigma Aldrich. Protein analysis was done with a linear gradient of 10-90% B in 25 min followed by 1 min at 90% B and 4 min of equilibration in 99% A.

The Synapt G2Si mass spectrometer (Waters) was operated with Masslynx software in positive resolution mode with the following settings: source temperature 80 °C, capillary voltage 3.0 kV, nano flow gas 0.5 Bar, purge gas 100 L/h, cone gas 0 L/h, sampling cone 40 V, source offset 80 V, lockspray voltage 3.5 kV, lock mass mixture Leu Enk (556.2771) and GluFib (785.84265). Protein charge state envelopes were acquired in a mass range of 400-3400 m/z with scan time of 3 seconds, lock mass was sampled every 30 seconds and the MaxEnt1 software was used for data deconvolution. The percentage conversion was calculated using the height of the deconvoluted protein peaks. For example, in main article Table 1 at 16 hr incubation the wild type protein reacted with compound **1** showed just one protein species having the mass of the wild-type protein suggesting that no covalent adduct between the protein and the compound was present. Possible ionic or van der Waals' interaction products might have been lost due to the denaturing conditions (0.1% formic acid and acetonitrile gradient) during the ESI ionization. With compound **2** we observed a population having the mass of the wild-type protein and a second population with 30% abundance with a mass corresponding to the wild-type - compound **2** adduct. The wild-type protein reacted with compound 3 resulted in 100% conversion of the wild-type protein mass to the wild-type-compound **3** adduct mass. We are confident that the observed protein-compound adducts are of covalent nature for several reasons: the denaturing conditions (acid, ACN) used for the LC separation, the presence of multiple charged states of the protein (see Supplemental Figure S3) ranging from 20 to 52 charges that support a denatured protein model,[9] and the absence of an adduct for compound **1** with the acid/base protein mutant that only differs from the wild-type by one amino acid, while the wild-type converts by 25 to 30% with this compound. Because the protein is measured in an unfolded, denatured state, ionic or van der Waals' interactions between protein and the saccharides will be lost and will not detected by the mass spectrometer.

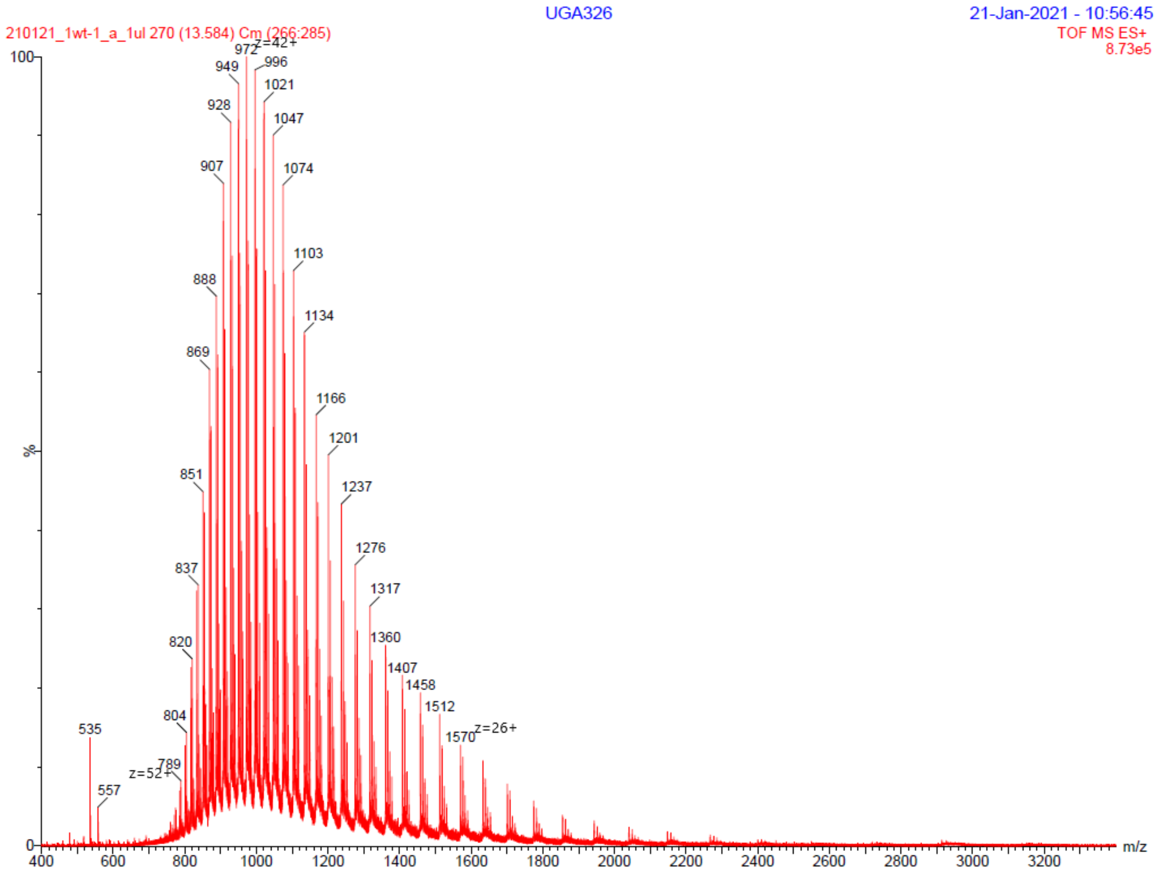

Supplemental Figure S3. Electrospray ionisation (ESI) measurement of wild type GH76 protein under denaturing conditions using LC-MS analysis. The charge states of the protein range from z = 26+ to z = 52+ with the peak at z = 42+ charges supporting an unfolded, denatured state of the proteins under these conditions.

3. Synthetic procedures

General methods

Chemicals were purchased from Acros, Sigma Aldrich, Biosolve, VWR, Fluka, Merck and Fisher Scientific and used as received unless stated otherwise. Tetrahydrofuran (THF), *N*,*N*-dimethylformamide (DMF) and toluene were stored over molecular sieves before use. Traces of water from reagents were removed by co-evaporation with toluene in reactions that required anhydrous conditions. All reactions were performed under a nitrogen atmosphere unless stated otherwise. TLC analysis was conducted using Merck aluminum sheets (Silica gel 60 F_{254}) with detection by UV absorption (254 nm), by spraying with a solution of $(NH_4)_6Mo_7O_{24}\cdot 4H_2O$ (25 g/L) and $(NH_4)_4Ce(SO_4)_4\cdot 2H_2O$ (10 g/L) in 10% sulfuric acid or a solution of $KMnO_4$ (20 g/L) and K_2CO_3 (10 g/L) in water, followed by charring at ~150 ˚C. Column chromatography was performed using Screening Device b.v. silica gel (particle size of 40 – 63 µm, pore diameter of 60 Å) with the indicated eluents. For reversed-phase HPLC purifications an Agilent Technologies 1200 series instrument equipped with a semi-preparative column (Gemini C18, 250 x 10 mm, 5 µm particle size, Phenomenex) was used. LC/MS analysis was performed on a Surveyor HPLC system (Thermo Finnigan) equipped with a C_{18} column (Gemini, 4.6 mm x 50 mm, 5 µm particle size, Phenomenex), coupled to a LCQ Adventage Max (Thermo Finnigan) ion-trap spectrometer (ESI^+). The applied buffers were H_2O, MeCN and 1% aqueous TFA. ^1H NMR and ^{13}C NMR spectra were recorded on a Brüker AV-300 (300 and 75 MHz respectively), a Brüker AV-400 (400 and 101 MHz respectively) or a Brüker AV-500 (500 and 126 MHz respectively) spectrometer in the given solvent. Chemical shifts are given in ppm (δ) relative to the residual solvent peak or tetramethylsilane (0 ppm) as internal standard. Coupling constants are given in Hz. High-resolution mass spectrometry (HRMS) analysis was performed with a LTQ Orbitrap mass spectrometer (Thermo Finnigan), equipped with an electrospray ion source in positive mode (source voltage 3.5 kV, sheath gas flow 10 mL/min, capillary temperature 250 °C) with resolution R = 60000 at m/z 400 (mass range m/z = 150 – 2000) and dioctyl phthalate (m/z = 391.28428) as a “lock mass”. The high-resolution mass spectrometer was calibrated prior to measurements with a calibration mixture (Thermo Finnigan).

Synthesis of inhibitors 1 and 2

Starting from diol **S1**,[10] the secondary alcohol was protected by a three-step procedure involving protection of the primary alcohol with TBS, benzylation of the secondary alcohol and desilylation to afford **7** (Supplemental Scheme 1). The olefin could be stereoselectively epoxidized by choice of reagent; using *m*-CPBA β-epoxide **S2** was selectively obtained, whereas α-epoxide **S3** was obtained by treatment with *in situ* generated (trifluoromethyl)methyldioxirane. Epoxide **S3** was subsequently coupled with trichloroimidate donor **10** to afford pseudo-disaccharide **S4**.[11] Zemplén deacetylation afforded **S5**, which was then debenzylated using Pearlman's catalyst to afford α-1,6-*mannobiose-epi*-cyclophellitol **1**. Similarly, epoxide **S3** was coupled to disaccharide donor **S6**,[12] however in this case the product was highly contaminated with homo-coupled donor byproducts which were inseparable with silica or size-exclusion chromatography. Deacetylation of this mixture did furnish **S7** in pure state, which gave α-1,6-*mannotriose-epi*-cyclophellitol **2** after debenzylation.

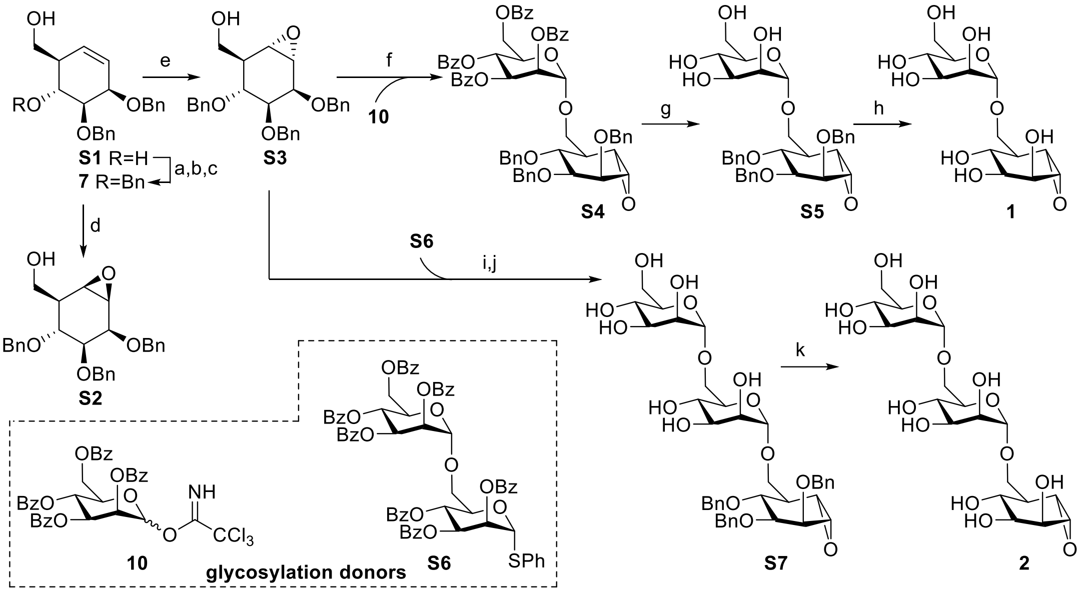

Supplemental Scheme 1. Synthesis of α-1,6-*mannotriose-epi*-cyclophellitols 1 and 2. Reagents and conditions: (a) TBSCl, imidazole, DMF; (b) BnBr, NaH, TBAI, DMF; (c) TBAF, THF, 89% over three steps; (d) *m*-CPBA, DCM , 0 °C, 84%; (e) Oxone, CH_3COCF_3, $NaHCO_3$, EDTA, H_2O, MeCN, 0 °C, 89%; (f) TMSOTf, DCM, -40 °C, 65%; (g) NaOMe, MeOH, DCM, 81%; (h) $Pd(OH)_2/C$, H_2, dioxane, MeOH, H_2O, 2h, quant; (i) NIS, TMSOTf, DCM, -40 °C; (j) NaOMe, MeOH, DCM, 14% over two steps; (k) $Pd(OH)_2/C$, H_2, dioxane, MeOH, H_2O, 2 h, quant.

Compound 7

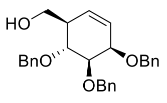

Diol **S1** (400 mg, 1.18 mmol)[10] was dissolved in dry DMF (6 mL), imidazole (200 mg, 2.94 mmol) was added and the mixture was cooled to -15 °C (ice/EtOH). Then, TBSCl (186 mg, 1.23 mmol) was added and the mixture was stirred for 2 h while the cooling bath was allowed to warm to rt. The mixture was quenched and diluted with H_2O (120 mL) and extracted with Et_2O (3 x 50 mL). The combined organic layers were washed with H_2O (100 mL) and brine, dried over $MgSO_4$, filtrated and concentrated. The crude product was co-evaporated with toluene, dissolved in dry DMF (6 mL) and cooled to 0 °C. Then, TBAI (43 mg, 0.12 mmol), BnBr (280 µL, 2.35 mmol) and NaH (60 wt%, 89 mg, 2.23 mmol) were added and the mixture was stirred overnight at rt. The reaction was quenched and diluted with H_2O (60 mL) at 0 °C, and extracted with Et_2O (3 x 30 mL). The combined organic layers were washed with H_2O (30 mL) and brine, dried over $MgSO_4$, filtrated and concentrated. The crude product was co-evaporated with toluene, dissolved in THF (6 mL) and TBAF (1M in THF, 3.5 mL, 3.5 mmol) was added. After 2 h, the mixture was concentrated, and flash purification by silica column chromatography (pentane/EtOAc, 3:1 → 2:1) afforded the title compound as an oil (451 mg, 89%). ^1H NMR (400 MHz, $CDCl_3$) δ 7.42 – 7.22 (m, 15H), 5.85 (ddd, J = 9.9, 4.6, 2.7 Hz, 1H), 5.66 (dd, J = 10.0, 2.5 Hz, 1H), 4.96 (d, J = 11.2 Hz, 1H), 4.80 – 4.62 (m, 5H), 4.13 (t, J = 4.0 Hz, 1H), 3.97 (dd, J = 8.9, 7.3 Hz, 1H), 3.76 – 3.63 (m, 3H), 2.46 – 2.36 (m, 1H), 2.09 (brs, OH) ppm. ^{13}C NMR (101 MHz, $CDCl_3$) δ 138.9, 138.6, 138.5, 130.5, 128.6, 128.5, 128.5, 128.3, 128.0, 127.9, 127.8, 127.7, 126.9, 80.7, 77.0, 74.5, 72.7, 72.0, 71.8, 64.5, 46.5 ppm. HRMS (ESI) m/z: $[M+Na]^+$ calc for $C_{28}H_{30}O_4$ 453.2036, found 453.2049.

Compound S2

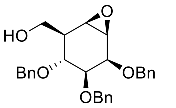

Cyclohexene **7** (451 mg, 1.05 mmol) was dissolved in DCM (10 mL) and cooled to 0 °C on a large ice-bath. Then, mCPBA (<77% wt, 470 mg, 2.1 mmol) was added and the mixture was stirred overnight while the cooling bath was slowly allowed to reach rt. The mixture was diluted with DCM (100 mL) and washed with a mixture of sat. aq. $NaHCO_3$ and aq. 10% $Na_2S_2O_3$ (1:1 v/v, 3 x 50 mL) and brine, dried over $MgSO_4$, filtrated and concentrated. Flash purification by silica column chromatography (pentane/EtOAc, 2:1 → 1:1) afforded the title compound as a white solid (393 mg, 84%). Additionally, α-epoxide **S3** was obtained as an oil (36 mg, 8%). ^{1}H NMR (400 MHz, $CDCl_3$) δ 7.48 – 7.23 (m, 15H), 4.88 (d, *J* = 11.0 Hz, 1H), 4.83 (d, *J* = 12.4 Hz, 1H), 4.72 (d, *J* = 12.4 Hz, 1H), 4.66 – 4.58 (m, 2H), 4.52 (d, *J* = 11.0 Hz, 1H), 4.03 (t, *J* = 4.6 Hz, 1H), 3.94 (dd, *J* = 10.7, 5.2 Hz, 1H), 3.88 (dd, *J* = 10.7, 6.2 Hz, 1H), 3.75 (t, *J* = 8.5 Hz, 1H), 3.45 (dd, *J* = 9.1, 4.9 Hz, 1H), 3.32 (dd, *J* = 3.7, 2.5 Hz, 1H), 3.20 (t, *J* = 4.1 Hz, 1H), 2.58 – 2.29 (brs, OH), 2.14 (ddt, *J* = 8.5, 5.4, 3.1 Hz, 1H) ppm. ^{13}C NMR (101 MHz, $CDCl_3$) δ 138.3, 138.2, 138.1, 128.5, 128.5, 128.4, 128.2, 128.2, 128.0, 127.9, 127.8, 80.0, 74.7, 74.2, 72.6, 71.5, 70.5, 63.0, 54.9, 51.0, 44.3 ppm. HRMS (ESI) m/z: $[M+Na]^+$ calc for $C_{28}H_{30}O_5$ 469.1985, found 469.1990.

Compound S3

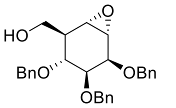

Cyclohexene **7** (326 mg, 0.76 mmol) was dissolved in a mixture of MeCN (7.6 mL) and aq. EDTA (0.4 mM, 3.8 mL) and cooled to 0 °C. 1,1,1-trifluoroacetone (1.0 mL, 11.4 mmol) was added via a pre-cooled needle and subsequently a mixture of oxone (2.33 g, 3.79 mmol) and $NaHCO_3$ (445 mg, 5.3 mmol) was added in 6 portions over 1 h. After stirring an additional hour, the mixture was diluted with H_2O (100 mL) and extracted with EtOAc (3 x 50 mL). The combined organic fractions were washed with brine, dried over $MgSO_4$, filtrated and concentrated. Flash purification by silica column chromatography (pentane/EtOAc, 4:1 → 2:1) afforded the title compound as an oil (302 mg, 89%). ^{1}H NMR (400 MHz, $CDCl_3$) δ 7.39 – 16 (m, 15H), 4.90 (dd, J = 18.0, 11.7 Hz, 2H), 4.73 – 4.53 (m, 4H), 4.22 (t, J = 2.6 Hz, 1H), 3.81 – 3.75 (m, 1H), 3.75 – 3.66 (m, 2H), 3.61 (dd, J = 10.8, 5.9 Hz, 1H), 3.19 (t, J = 3.1 Hz, 1H), 3.11 (d, J = 3.7 Hz, 1H), 2.27 (brs, OH), 2.19 (dt, J = 7.8, 5.7 Hz, 1H) ppm. ^{13}C NMR (101 MHz, $CDCl_3$) δ 138.5, 138.4, 138.3, 128.5, 128.5, 128.4, 128.2, 127.9, 127.9, 127.8, 127.7, 127.7, 80.2, 74.8, 74.7, 74.0, 73.5, 73.1, 62.6, 54.3, 54.2, 44.3 ppm. HRMS (ESI) m/z: $[M+Na]^+$ calc for $C_{28}H_{30}O_5$ 469.1985, found 469.1996.

Compound S4

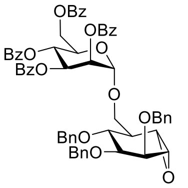

Acceptor **S3** (45 mg, 0.1 mmol) and trichloroimidate donor **10** (89 mg, 0.12 mmol)[11] were combined in a flask, co-evaporated with toluene (3x) and dissolved in dry DCM (1 mL). The mixture was cooled to -40 °C and TMSOTf (5.4 µL, 30 µmol) was added. After stirring for 1 h, the reaction was quenched with Et_3N (50 µL) and warmed to rt. The mixture was diluted with H_2O (30 mL) and extracted with DCM (3 x 15 mL). The combined organic fractions were washed with brine, dried over $MgSO_4$, filtrated and concentrated. Flash purification by silica column chromatography (pentane/EtOAc, 5:1) afforded the title compound as an oil (67 mg, 65%). ^{1}H NMR (500 MHz, $CDCl_3$) δ 8.07 (ddd, J = 12.9, 8.3, 1.2 Hz, 4H), 7.92 (dd, J = 8.3, 1.2 Hz, 2H), 7.82 (dd, J = 8.3, 1.2 Hz, 2H), 7.62 – 7.57 (m, 1H), 7.56 – 7.51 (m, 1H), 7.50 – 7.46 (m, 1H), 7.45 – 7.22 (m, 26H), 6.13 (t, J = 10.0 Hz, 1H), 5.90 (dd, J = 10.2, 3.3 Hz, 1H), 5.73 (dd, J = 3.2, 1.8 Hz, 1H), 5.08 (d, J = 1.7 Hz, 1H), 4.97 (dd, J = 11.9, 4.1 Hz, 2H), 4.77 (d, J = 12.3 Hz, 1H), 4.72 (d, J = 11.7 Hz, 1H), 4.63 (dd, J = 21.5, 11.6 Hz, 3H), 4.44 – 4.36 (m, 2H), 4.28 (t, J = 2.6 Hz, 1H), 3.91 (dd, J = 9.9, 7.5 Hz, 1H), 3.85 – 3.80 (m, 1H), 3.77 (dd, J = 9.6, 3.0 Hz, 2H), 3.29 (t, J = 3.1 Hz, 1H), 3.27 (d, J = 3.5 Hz, 1H), 2.44 (td, J = 7.8, 3.9 Hz, 1H) ppm. ^{13}C NMR (125 MHz, $CDCl_3$) δ 166.2, 165.6, 165.5, 165.5, 138.7, 138.6, 133.6, 133.5, 133.3, 133.2, 130.0, 129.9, 129.9, 129.4, 129.2, 129.1, 128.7, 128.6, 128.6, 128.4, 128.1, 128.0, 127.9, 127.9, 127.8, 98.0, 80.4, 75.0, 74.6, 74.0, 73.3, 73.2, 70.5, 70.1, 69.4, 68.3, 66.9, 62.8, 54.5, 54.2, 42.4 ppm. HRMS (ESI) m/z: $[M+Na]^+$ calc for $C_{62}H_{56}O_{14}$ 1047.3562, found 1047.3627.

Compound S5

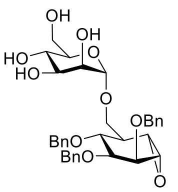

Compound **S4** (56 mg, 55 µmol) was dissolved in a mixture of MeOH (0.5 mL) and DCM (0.5 mL), NaOMe (5.4 M in MeOH, 5.1 µL, 27 µmol) was added and the mixture was stirred overnight at rt. The mixture was concentrated and flash purification by silica column chromatography (DCM/MeOH, 19:1) afforded the title compound as an oil (27 mg, 81%). ^{1}H NMR (500 MHz, $CDCl_3$ + MeOD) δ 7.41 – 7.24 (m, 15H), 4.89 (d, J = 11.5 Hz, 2H), 4.80 (m, under solvent peak, assigned by HSQC, 1H), 4.72 (d, J = 12.0 Hz, 1H), 4.63 (d, J = 2.8 Hz, 2H), 4.61 – 4.56 (m, 1H), 4.28 (t, J = 2.7 Hz, 1H), 3.88 – 3.81 (m, 2H), 3.81 – 3.72 (m, 4H), 3.69 (ddd, J = 9.5, 6.0, 3.1 Hz, 2H), 3.59 (dd, J = 9.8, 3.9 Hz, 1H), 3.54 (ddd, J = 9.5, 4.5, 2.7 Hz, 1H), 3.23 (t, J = 3.1 Hz, 1H), 3.16 (d, J = 3.6 Hz, 1H), 2.27 (td, J = 7.8, 3.9 Hz, 1H) ppm. ^{13}C NMR (125 MHz, $CDCl_3$ + MeOD) δ 137.9, 137.8, 137.8, 127.7, 127.6, 127.6, 127.3, 127.3, 127.1, 127.1, 126.9, 126.9, 99.8, 79.5, 74.2, 74.0, 73.1, 72.8, 72.5, 72.1, 70.8, 70.2, 66.5, 66.1, 60.8, 53.5 (2 C), 41.8 ppm. HRMS (ESI) m/z: $[M+Na]^+$ calc for $C_{34}H_{40}O_{10}$ 631.2514, found 631.2531.

Compound 1

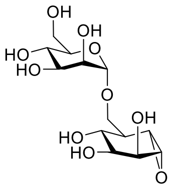

Compound **S5** (20 mg, 33 µmol) was dissolved in a mixture of dioxane/MeOH/H_2O (1:1:1, 1.5 mL) under N_2. Pd(OH)2/C (20 wt%, 12 mg, 16 µmol) was added and the mixture was purged with H_2 gas with a balloon. After stirring vigorously for 2 h, the mixture was filtrated over Celite and evaporated, affording the title compound as an oil (12 mg, quant.). ^{1}H NMR (400 MHz, D_2O) δ 4.89 (d, J = 1.6 Hz, 1H), 4.41 (t, J = 2.9 Hz, 1H), 3.97 – 3.90 (m, 2H), 3.89 – 3.78 (m, 2H), 3.76 – 3.68 (m, 2H), 3.65 – 3.57 (m, 3H), 3.51 (dd, J = 10.4, 3.5 Hz, 1H), 3.40 – 3.36 (m, 1H), 3.31 (d, J = 3.5 Hz, 1H), 2.14 (ddd, J = 9.4, 6.3, 3.3 Hz, 1H) ppm. ^{13}C NMR (101 MHz, D_2O) δ 99.7, 72.9, 70.5, 70.3, 70.0, 67.1, 66.7, 66.1, 65.6, 60.9, 55.8, 55.2, 42.3 ppm. HRMS (ESI) m/z: $[M+Na]^+$ calc for $C_{13}H_{22}O_{10}$ 361.1105, found 361.1118.

Compound S7

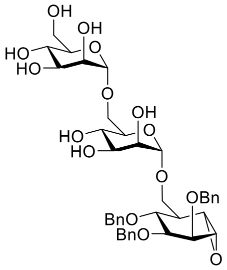

Acceptor **S3** (62 mg, 0.14 mmol) and thioglycoside donor **S6** (194 mg, 0.17 mmol)[12] were combined in a flask, co-evaporated with toluene (3x) and dissolved in dry DCM (1.4 mL). Molecular sieves (4 Å) were added and the mixture was stirred for 1 h at rt, and subsequently cooled to -40 °C. Then, NIS (36 mg, 0.17 mmol) and TMSOTf (7.5 µL, 42 µmol) were added. After stirring for 1 h, the reaction was quenched with Et_3N (50 µL) and warmed to rt. The mixture was diluted DCM (50 mL), washed with 10% aq. $Na_2S_2O_3$ (2 x 20 mL) and brine, dried over $MgSO_4$, filtrated and concentrated. Flash purification by silica column chromatography (pentane/EtOAc, 2:1) afforded the target trisaccharide which was highly contaminated with inseparable byproducts. The product was taken up in a mixture of MeOH (0.5 mL) and DCM (0.5 mL) and 5 drops of NaOMe (5.4 M in MeOH) were added. After stirring the reaction overnight, the reaction was neutralized by addition of Amberlite CG-50 (H^+), filtrated and concentrated. Flash purification by silica column chromatography (DCM/MeOH, 19:1 → 15:85) afforded the title compound as an oil (15 mg, 14%). ^{1}H NMR (400 MHz, MeOD) δ 7.44 – 7.18 (m, 15H), 4.87 (d, J = 2.4 Hz, 1H), 4.79 (d, J = 1.5 Hz, 1H), 4.78 – 4.70 (m, 2H), 4.70 – 4.55 (m, 4H), 4.30 (t, J = 2.8 Hz, 1H), 3.90 (dd, J = 11.2, 5.4 Hz, 1H), 3.88 – 3.79 (m, 4H), 3.78 (d, J = 8.5 Hz, 1H), 3.75 – 3.68 (m, 4H), 3.68 – 3.61 (m, 5H), 3.57 (dd, J = 9.9, 3.7 Hz, 1H), 3.24 (t, J = 3.1 Hz, 1H), 3.15 (d, J = 3.6 Hz, 1H), 2.22 (td, J = 7.6, 3.8 Hz, 1H) ppm. ^{13}C NMR (101 MHz, MeOD) δ 140.0, 139.9, 139.8, 129.5, 129.4, 129.4, 129.2, 129.1, 129.0, 128.8, 128.7, 128.7, 101.9, 101.4, 81.4, 76.0, 75.8, 74.7, 74.4, 74.3, 73.7, 73.5, 72.8, 72.6, 72.0, 72.0, 68.6, 68.3, 67.9, 67.0, 62.8, 55.4, 55.2, 43.9 ppm. HRMS (ESI) m/z: $[M+Na]^+$ calc for $C_{40}H_{50}O_{15}$ 793.3042, found 793.3082.

Compound 2

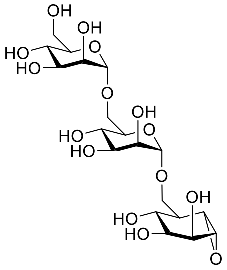

Compound **S7** (10 mg, 13 µmol) was dissolved in a mixture of dioxane/MeOH/H_2O (1:1:1, 1.0 mL) under N_2. $Pd(OH)_2/C$ (20 wt%, 10 mg, 14 µmol) was added and the mixture was purged with H_2 gas with a balloon. After stirring vigorously for 2 h, the mixture was filtrated over celite and evaporated, affording the title compound as an oil (6.9 mg, quant.). ^{1}H NMR (500 MHz, D_2O) δ 4.91 (s, 2H), 4.44 (t, J = 2.9 Hz, 1H), 4.02 – 3.91 (m, 4H), 3.89 (d, J = 10.8 Hz, 1H), 3.85 (t, J = 3.4 Hz, 1H), 3.83 (t, J = 3.1 Hz, 1H), 3.80 – 3.64 (m, 6H), 3.68 – 3.60 (m, 2H), 3.53 (dd, J = 10.4, 3.4 Hz, 1H), 3.41 (t, J = 3.0 Hz, 1H), 3.34 (d, J = 3.6 Hz, 1H), 2.17 (ddd, J = 9.2, 6.2, 3.2 Hz, 1H) ppm. ^{13}C NMR (125 MHz, D_2O) δ 99.9, 99.5, 72.8, 71.1, 70.9, 70.6, 70.4, 70.0, 67.2, 66.8, 66.6, 66.2, 65.7, 65.6, 61.0, 55.9, 55.3, 42.3 ppm. HRMS (ESI) m/z: $[M+Na]^+$ calc for $C_{19}H_{32}O_{15}$ 523.1633, found 523.1649.

Synthesis of inhibitor 3

Recently, Di Bussolo and coworkers described a mild copper(II)triflate mediated regioselective opening of a benzyl protected 1,2-deoxy-carbaglucose-1,2-epoxide by azidoethanol.[13] Analogously, epoxide 'donor' **6** was prepared with an orthogonal triisopropylsilyl (TIPS) group at C6 to enable elongation towards the pseudotrisaccharide at a later stage (Supplemental Scheme 2). For this, carba-glucal **4** was protected with a TIPS group to give **S8**.[14] Direct epoxidation using *m*-CPBA or methyl(trifluoromethyl)dioxirane generated *in situ* resulted in a 1:3 ratio of epoxide **6** and its epimer, respectively. Instead, removal of the benzyl protecting groups in **S8** under Birch conditions using lithium/naphthalene followed by stereoselective epoxidation directed by the liberated allylic alcohol gave compound **S9** in high yield, and following benzylation epoxide **6** was obtained. 'Donor' **6** was coupled with 'acceptor' **7** (see Supplemental Scheme 1) using a catalytic amount of $Cu(OTf)_2$ under slightly elevated temperatures,[13] affording **8**. Benzoylation followed by desilylation gave **9**, which was glycosylated with **10** (see Supplemental Scheme 1) giving pseudo-trisaccharide **11** in high yield. The stereoselective α-epoxidation method using methyl(trifluoromethyl)dioxirane, as was applied for the synthesis of **S3** (see Supplemental Scheme 1), required slightly modified reaction conditions when applied to **11** due to the poor solubility of **11** in the reaction medium. Nonetheless, epoxide **S10** was obtained in good yield as a single diastereoisomer. Debenzoylation gave **S11**, which was subjected to hydrogenolysis to afford stabilized α-1,6-mannotriose-epi-cyclophellitol **3**.

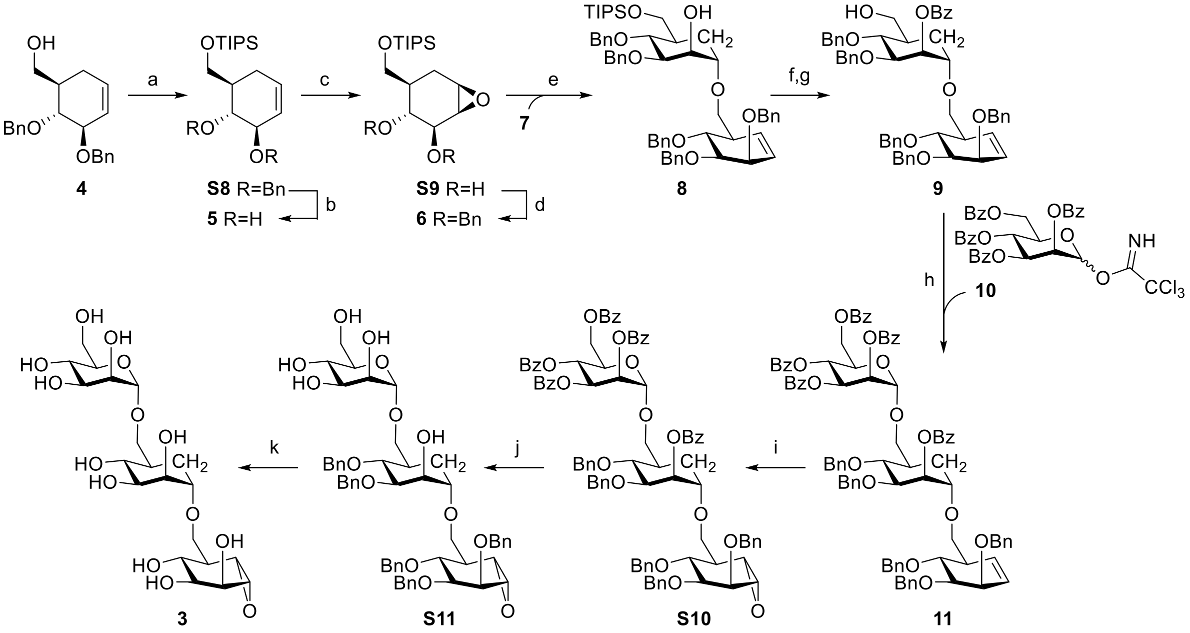

Supplemental Scheme 2. Synthesis of stabilized α-1,6-*mannotriose-epi*-cyclophellitol 3. Reagents and conditions: (a) TIPS-Cl, imidazole, DMF, quant.; (b) Li, naphthalene, THF, -78 to -20 °C; (c) *m*-CPBA, DCM, -20 °C to rt, 93% over two steps; (d) BnBr, NaH, TBAI, THF, 0 °C to rt, 75%; (e) acceptor **7** (2 eq.), $Cu(OTf)_2$, toluene, 40 °C, 66%; (f) BzCl, pyridine, *N*-methylimidazole, DCM; (g) TBAF, THF, 70% over two steps; (h) donor 10, TMSOTf, DCM, -30 °C, 1h, 96%; (i) 1,1,1-trifluoroacetone, oxone, $NaHCO_3$, EDTA, H_2O, MeCN, EtOAc, 0 °C, 75%; (j) NaOMe, MeOH, DCM, 83%; (k) $Pd(OH)_2/C$, H_2, MeOH, dioxane, H_2O, 85%.

Compound S8

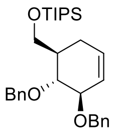

Carba-glucal **4** (3.7 g, 11.4 mmol)[14] was dissolved in DMF and cooled to 0 °C. Imidazole (1.16 g, 17.1 mmol) and TIPS-Cl (3.17 mL, 14.8 mmol) were subsequently added and the mixture was stirred overnight whilst the cooling bath was slowly warmed to rt. The reaction was quenched with MeOH (4 mL), diluted with H_2O (400 mL) and extracted with Et_2O (3 x 100 mL). The combined organic layers were washed with brine, dried over $MgSO_4$, filtered and concentrated. Flash purification by silica column chromatography (pentane:EtOAc, 100:0 → 100:1) afforded the title compound as an oil (5.53 g, quant.). ^1H NMR (400 MHz, $CDCl_3$) δ 7.46 – 7.15 (m, 10H), 5.78 (ddd, *J* = 8.9, 3.9, 2.0 Hz, 1H), 5.73 – 5.60 (m, 1H), 4.93 (d, *J* = 11.1 Hz, 1H), 4.77 – 4.62 (m, 3H), 4.20 (dq, *J* = 7.2, 2.8, 2.4 Hz, 1H), 3.91 (dd, *J* = 9.7, 5.6 Hz, 1H), 3.82 (dd, *J* = 9.7, 3.0 Hz, 1H), 3.67 (dd, *J* = 10.8, 7.4 Hz, 1H), 2.24 (dt, *J* = 8.3, 2.6 Hz, 2H), 2.07 – 1.86 (m, 1H), 1.16 – 0.98 (m, 21H) ppm. ^{13}C NMR (101 MHz, $CDCl_3$) δ 139.3, 138.8, 128.9, 128.5, 128.4, 128.0, 127.7, 127.6, 126.1, 81.7, 79.5, 74.5, 71.7, 63.6, 41.6, 28.7, 18.3, 12.2 ppm. HRMS (ESI) m/z: $[M+H]^+$ calc for $C_{30}H_{45}O_3Si$ 481.31325, found 481.31319.

Compound 5 and S9

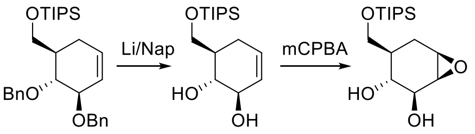

Naphthalene (4.0 g, 31.2 mmol) was dissolved in dry distilled THF (48 mL) in a round bottom flask equipped with a glass stirring bar. Lithium wire (655 mg, 93.6 mmol) was pre-rinsed with pentane and added in pieces to the naphthalene mixture. The mixture was sonicated for 5 minutes, resulting in a dark green color, and then the mixture was cooled to -78 °C. To this mixture was added a solution of compound **S8** (2.0 g, 4.2 mmol) in dry distilled THF (16 mL). The mixture was allowed to warm to -20 °C and stirred overnight. The reaction was quenched with 1M NH_4Cl (20 mL) and diluted with EtOAc (30 mL). The remaining chunks of lithium were manually removed with pincers and quenched with water. The reaction was poured into brine (100 mL) and extracted with EtOAc (4 x 40 mL). The combined organic layers were dried with $MgSO_4$, filtered and concentrated. Flash purification by silica column chromatography (pentane:EtOAc, 4:1 → 3:1) afforded the title compound as an oil (1.31 g, yield higher than quantitative) which was contaminated with unknown inseparable byproducts. ^{1}H NMR (400 MHz, $CDCl_3$) δ 5.85 – 5.44 (m, 2H), 4.41 (br s, 1H, OH), 4.20 (ddd, *J* = 7.6, 3.7, 1.8 Hz, 1H), 3.95 – 3.72 (m, 2H), 3.69 – 3.54 (m, 1H), 2.98 (br s, 1H, OH), 2.09 (dt, *J* = 17.6, 4.6 Hz, 1H), 1.97 (ddt, *J* = 10.9, 5.4, 3.4 Hz, 1H), 1.90 – 1.72 (m, 1H), 1.19 – 0.87 (m, 21H) ppm. ^{13}C NMR (101 MHz, $CDCl_3$) δ 128.6, 126.7, 77.8, 73.9, 67.4, 39.9, 27.8, 18.0, 11.8 ppm. HRMS (ESI) m/z: $[M+Na]^+$ calc for $C_{16}H_{32}O_3SiNa$ 323.20129, found 323.20123. This product was taken up in DCM (42 mL) and cooled to -20 °C (ice/salt). *m*-CPBA (<77 wt%, 1.86 g, 8.3 mmol) was added and the mixture was stirred overnight whilst the cooling bath was allowed to warm to 0 °C. The reaction was quenched with aq. 10% $Na_2S_2O_3$ (10 mL), diluted with DCM (60 mL) and washed with sat. aq. $NaHCO_3$ (2 x 40 mL) and brine. The combined organic layers were dried over $MgSO_4$, filtered and concentrated. Flash purification by silica column chromatography (pentane:EtOAc, 3:1 → 2:1) afforded the title compound as an oil (1.22 g, 93% over two steps). ^{1}H NMR (400 MHz, $CDCl_3$) δ 4.00 (br s, 1H, OH), 3.85 (d, *J* = 8.0 Hz, 1H), 3.81 – 3.55 (m, 3H), 3.45 – 3.29 (m, 2H), 3.28 (t, *J* = 4.4 Hz, 1H), 2.03 (dt, *J* = 14.7, 5.6 Hz, 1H), 1.84 – 1.52 (m, 2H), 1.06 (s, 21H) ppm. ^{13}C NMR (101 MHz, $CDCl_3$) δ 74.6, 73.4, 66.1, 56.7, 53.2, 40.4, 25.8, 18.0, 11.8 ppm. HRMS (ESI) m/z: $[M+Na]^+$ calc for $C_{16}H_{32}O_4Si$ 339.19621, found 339.19582.

Compound 6

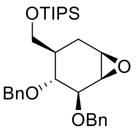

Compound **S9** (1.22 g, 3.85 mmol) was co-evaporated with toluene and dissolved in dry THF (19 mL). Then, BnBr (1.4 mL, 11.6 mmol) and TBAI (142 mg, 0.39 mmol) were added and the mixture was cooled to 0 °C. Sodium hydride (60 wt%, 316 mg, 7.9 mmol) was added and the mixture was stirred overnight whilst the cooling bath was allowed to warm to rt. The reaction was quenched with 1M NH_4Cl (5 mL) at 0 °C, diluted with H_2O (50 mL) and brine (50 mL) and extracted with EtOAc (3 x 50 mL). The combined organic layers were washed with brine, dried over $MgSO_4$, filtered and concentrated. Flash purification by silica column chromatography (pentane:Et_2O, 15:1) afforded the title compound as an oil (1.44 g, 75%). ^{1}H NMR (400 MHz, $CDCl_3$) δ 7.48 – 7.19 (m, 10H), 4.87 (dd, *J* = 10.9, 2.1 Hz, 1H), 4.82 (s, 2H), 4.61 (dd, *J* = 10.9, 2.1 Hz, 1H), 3.84 (dd, *J* = 8.1, 1.9 Hz, 1H), 3.76 – 3.66 (m, 2H), 3.62 (dd, *J* = 11.0, 8.2 Hz, 1H), 3.32 (dd, *J* = 3.9, 1.6 Hz, 1H), 3.27 (t, *J* = 4.3 Hz, 1H), 2.18 – 1.94 (m, 2H), 1.80 – 1.63 (m, 1H), 1.12 – 0.90 (m, 21H) ppm. ^{13}C NMR (101 MHz, $CDCl_3$) δ 138.9, 138.6, 128.5, 128.4, 128.1, 128.0, 127.8, 127.7, 82.0, 81.9, 77.7, 75.2, 72.6, 63.4, 55.4, 53.7, 42.1, 26.9, 18.2, 12.1 ppm. HRMS (ESI) m/z: $[M+H]^+$ calc for $C_{30}H_{45}O_4Si$ 497.30816, found 497.30812.

Compound 8

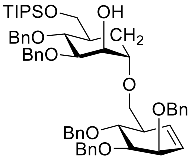

Epoxide 'donor' **6** (117 mg, 0.236 mmol) was combined with 'acceptor' **7** (203 mg, 0.471 mmol), co-evaporated with dry toluene (3x) and dissolved in dry toluene (471 µL) under argon. Then, $Cu(OTf)_2$ (4 mg, 12 µmol, 5 mol%) was added and the mixture was stirred for 16 h at 40 °C. The mixture was diluted with toluene (40 mL), washed with aq. sat. $NaHCO_3$ (20 mL) and brine. The organic layer was dried over $MgSO_4$, filtered and concentrated. Flash purification by silica column chromatography (pentane:EtOAc, 15:1 → 9:1) afforded the title compound as an oil (144 mg, 66%). ^{1}H NMR (400 MHz, $CDCl_3$) δ 7.39 – 7.21 (m, 25H), 5.73 (d, *J* = 2.9 Hz, 2H), 4.97 (d, *J* = 11.3 Hz, 1H), 4.88 (d, *J* = 10.9 Hz, 1H), 4.74 – 4.56 (m, 8H), 4.12 – 3.84 (m, 2H), 3.97 – 3.83 (m, 2H), 3.78 – 3.69 (m, 2H), 3.69 – 3.64 (m, 2H), 3.64 – 3.58 (m, 1H), 3.52 – 3.40 (m, 2H), 2.49 (br s, 1H, OH), 2.45 – 2.35 (m, 1H), 1.92 – 1.82 (m, 2H), 1.82 – 1.67 (m, 1H), 1.15 – 0.95 (m, 21H) ppm. ^{13}C NMR (101 MHz, $CDCl_3$) δ 139.2, 139.0, 138.9, 138.7, 138.4, 131.7, 128.5, 128.4, 128.4, 128.4, 128.3, 128.1, 128.0, 127.9, 127.8, 127.8, 127.7, 127.6, 127.6, 127.6, 127.5, 125.2, 82.6, 81.5, 77.5, 76.6, 75.4, 75.0, 74.7, 72.5, 72.4, 71.5, 71.5, 69.4, 69.4, 63.4, 45.0, 39.4, 26.6, 18.2, 12.1 ppm. HRMS (ESI) m/z: $[M+H]^+$ calc for $C_{58}H_{74}O_8Si$ 927.52257, found 927.52094.

Compound 9

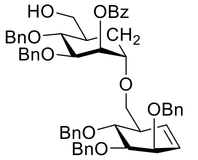

Compound **8** (143 mg, 0.154 mmol) was dissolved in dry DCM (1 mL), then pyridine (31 µL, 0.39 mmol) and benzoyl chloride (36 µL, 0.31 mmol) were added and the mixture was stirred overnight. Then, more pyridine (31 µL, 0.39 mmol) and benzoyl chloride (36 µL, 0.31 mmol) was added, as well as N-methylimidazole (31 µL, 0.39 mmol). After 6 h, the mixture was quenched with methanol (0.3 mL), diluted with DCM (40 mL), washed with 1M HCl (3 x 20 mL), sat. aq. $NaHCO_3$ (20 mL) and brine. The organic layer was dried over $MgSO_4$, filtered and concentrated. The crude was dissolved in dry THF (1.5 mL) and TBAF (1M in THF, 308 µL, 0.31 mmol) was added, and the mixture was stirred overnight. Then, more TBAF (1M in THF, 462 µL, 0.46 mmol) was added and the mixture was stirred overnight. The reaction was diluted with EtOAc (50 mL), washed with H_2O/brine (1:1, v/v, 3 x 20 mL) and brine. The organic layer was dried with $MgSO_4$, filtered and concentrated. Flash purification by silica column chromatography (pentane:EtOAc, 4:1) afforded the title compound as an oil (94 mg, 70% over two steps). ^1H NMR (400 MHz, $CDCl_3$) δ 8.20 – 8.04 (m, 2H), 7.74 – 7.19 (m, 28H), 5.90 – 5.65 (m, 3H), 5.07 (d, *J* = 11.4 Hz, 1H), 5.02 (d, *J* = 11.1 Hz, 1H), 4.88 – 4.66 (m, 7H), 4.62 (d, *J* = 11.4 Hz, 1H), 4.19 (t, *J* = 4.1 Hz, 1H), 4.08 – 3.96 (m, 2H), 3.86 (t, *J* = 9.8 Hz, 1H), 3.82 – 3.71 (m, 2H), 3.70 – 3.61 (m, 3H), 3.61 – 3.54 (m, 1H), 2.55 – 2.45 (m, 1H), 2.15 – 2.05 (m, 1H), 1.82 (d, *J* = 14.1 Hz, 1H), 1.78 – 1.64 (m, 1H) ppm. ^{13}C NMR (101 MHz, $CDCl_3$) δ 165.5, 139.0, 138.9, 138.7, 138.4, 138.2, 133.3, 131.5, 130.2, 129.8, 128.6, 128.6, 128.5, 128.4, 128.4, 128.4, 128.2, 128.1, 127.9, 127.9, 127.8, 127.7, 127.6, 127.6, 127.6, 125.5, 81.5, 80.2, 78.9, 75.1, 74.8, 74.8, 74.7, 72.4, 71.9, 71.5, 71.4, 69.9, 69.6, 65.1, 44.9, 38.7, 27.4 ppm. HRMS (ESI) m/z: $[M+Na]^+$ calc for $C_{56}H_{58}O_9$ 897.39730, found 897.39723.

Compound 11

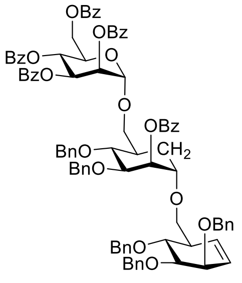

Acceptor **9** (93 mg, 0.11 mmol) and donor **10** (110 mg, 0.15 mmol)[11] were combined and co-evaporated with toluene (3x). The mixture was dissolved in dry DCM (1 mL), 3 Å molecular sieves were added and the mixture was stirred for 1 h at rt. Then, the mixture was cooled to -30 °C and TMSOTf (4 µL, 21 µmol) was added. The reaction was stirred for 1 h at -30 °C and then quenched with Et_3N (20 µL) and warmed to rt. The mixture was diluted with DCM (40 mL) and washed with brine. The organic layer was dried with $MgSO_4$, filtered and concentrated. Flash purification by silica column chromatography (pentane:EtOAc, 5:1) afforded the title compound as an oil (148 mg, 96%). ^1H NMR (400 MHz, $CDCl_3$) δ 8.21 (dd, *J* = 8.0, 1.4 Hz, 2H), 8.13 – 7.99 (m, 5H), 7.92 – 7.78 (m, 5H), 7.62 – 7.18 (m, 38H), 6.15 (t, *J* = 10.1 Hz, 1H), 5.96 – 5.84 (m, 2H), 5.84 – 5.69 (m, 3H), 5.19 (d, *J* = 11.8 Hz, 1H), 5.08 (d, *J* = 1.3 Hz, 1H), 5.02 (d, *J* = 11.4 Hz, 1H), 4.81 (d, *J* = 11.1 Hz, 1H), 4.78 – 4.62 (m, 7H), 4.56 (d, *J* = 11.2 Hz, 1H), 4.46 (dd, *J* = 12.2, 2.0 Hz, 1H), 4.31 (dd, *J* = 9.4, 3.3 Hz, 1H), 4.24 (dt, *J* = 10.1, 2.8 Hz, 1H), 4.20 – 4.10 (m, 2H), 4.10 – 4.03 (m, 2H), 4.02 – 3.92 (m, 1H), 3.75 – 3.63 (m, 3H), 3.63 – 3.48 (m, 1H), 3.38 (d, *J* = 8.0 Hz, 1H), 2.52 – 2.44 (m, 1H), 2.26 – 2.16 (m, 1H), 2.17 – 2.06 (m, 1H), 1.89 (d, *J* = 13.5 Hz, 1H) ppm. ^{13}C NMR (101 MHz, $CDCl_3$) δ 166.1, 165.8, 165.5, 165.4, 165.4, 139.1, 139.0, 138.9, 138.7, 138.1, 133.5, 133.5, 133.3, 133.2, 133.1, 131.6, 130.1, 130.0, 129.9, 129.9, 129.8, 129.8, 129.8, 129.3, 129.2, 128.9, 128.8, 128.7, 128.5, 128.4, 128.4, 128.4, 128.1, 127.9, 127.8, 127.7, 127.6, 127.6, 127.5, 127.0, 125.6, 98.4, 81.5, 80.4, 77.5, 75.1, 75.0, 74.8, 74.7, 72.4, 72.0, 71.4, 71.4, 70.5, 70.3, 69.7, 69.5, 69.0, 68.9, 66.5, 62.4, 45.0, 36.9, 28.2 ppm. HRMS (ESI) m/z: $[M+Na]^+$ calc for $C_{90}H_{84}O_{18}$ 1475.55499, found 1475.55682.

Compound S10

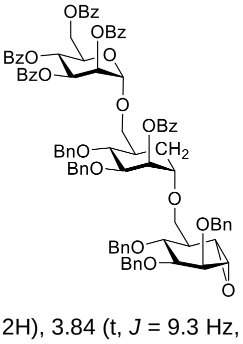

Compound **11** (73 mg, 50 µmol) was dissolved in EtOAc (1 mL), then aq. 0.4 mM EDTA (1 mL) was added and the mixture was cooled to 0 °C. A mixture of oxone (154 mg, 0.25 mmol) and $NaHCO_3$ (30 mg, 0.35 mmol) was added in 6 portions over 60 minutes. Additionally, 1,1,1-trifluoroacetone (6 x 45 µL, in total 3.0 mmol) was added in 6 portions. 15 minutes after the last addition, the mixture was diluted with EtOAc (50 mL), washed with aq. 10% $Na_2S_2O_3$ (3 x 20 mL) and brine. Flash purification by silica column chromatography (pentane:EtOAc, 5:1) afforded the title compound as an oil (55 mg, 75%). ^1H NMR (400 MHz, $CDCl_3$) δ 8.19 (d, *J* = 6.7 Hz, 2H), 8.06 (dd, *J* = 17.6, 7.4 Hz, 5H), 7.85 (t, *J* = 8.0 Hz, 5H), 7.63 – 7.14 (m, 38H), 6.14 (t, *J* = 10.1 Hz, 1H), 5.88 (dd, *J* = 10.2, 3.1 Hz, 1H), 5.83 (br s, 1H), 5.79 (br s, 1H), 5.17 (d, *J* = 11.8 Hz, 1H), 5.05 (s, 1H), 4.99 – 4.90 (m, 2H), 4.82 – 4.58 (m, 7H), 4.54 (d, *J* = 11.2 Hz, 1H), 4.44 (d, *J* = 10.7 Hz, 1H), 4.31 – 4.17 (m, 4H), 4.16 – 4.09 (m, 1H), 4.07 – 3.96 (m, 2H), 3.84 (t, *J* = 9.3 Hz, 1H), 3.73 (dd, *J* = 10.0, 2.6 Hz, 1H), 3.66 (dd, *J* = 8.9, 3.4 Hz, 1H), 3.63 – 3.55 (m, 2H), 3.30 (d, *J* = 8.8 Hz, 1H), 3.16 (t, *J* = 2.9 Hz, 1H), 3.04 (d, *J* = 3.4 Hz, 1H), 2.28 (d, *J* = 8.7 Hz, 1H), 2.23 – 1.99 (m, 2H), 1.82 (d, *J* = 13.2 Hz, 1H) ppm. ^{13}C NMR (101 MHz, $CDCl_3$) δ 166.2, 165.8, 165.5, 165.5, 139.1, 138.8, 138.7, 138.7, 138.0, 133.6, 133.5, 133.3, 133.2, 133.1, 130.1, 130.0, 130.0, 129.9, 129.8, 129.4, 129.3, 129.0, 128.9, 128.7, 128.6, 128.5, 128.5, 128.5, 128.3, 127.9, 127.9, 127.8, 127.8, 127.8, 127.7, 127.5, 127.0, 98.4, 80.7, 80.2, 75.2, 75.0, 74.9, 74.0, 73.6, 73.1, 72.1, 70.5, 70.3, 69.5, 69.1, 68.8, 68.7, 66.5, 62.4, 54.7, 54.4, 42.9, 37.0, 28.3 ppm. HRMS (ESI) m/z: $[M+H]^+$ calc for $C_{90}H_{84}O_{19}$ 1469.56796, found 1469.56795.

Compound S11

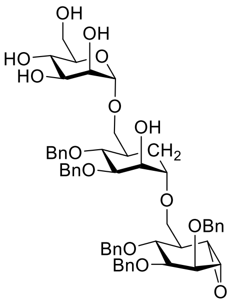

Compound **S10** (54 mg, 37 µmol) was dissolved in DCM (367 µL) and MeOH (367 µL) and NaOMe (5.4 M in MeOH, 5.1 µL, 28 µmol) was added. After stirring for 2 days, more NaOMe (10.2 µmol, 56 µmol) was added and after 6 h, the reaction was quenched with $Et_3N.HCl$ (13 mg). The mixture was evaporated and flash purification by silica column chromatography (DCM:MeOH, 19:1) afforded the title compound as an oil (29 mg, 83%). ^1H NMR (400 MHz, CD_3OD) δ 7.28 (s, 25H), 4.88 – 4.79 (m, 3H), 4.73 – 4.67 (m, 2H), 4.67 – 4.59 (m, 3H), 4.59 – 4.48 (m, 3H), 4.26 (t, *J* = 2.7 Hz, 1H), 4.11 (br s, 1H), 3.90 – 3.83 (m, 1H), 3.83 – 3.80 (m, 1H), 3.79 – 3.76 (m, 1H), 3.75 – 3.61 (m, 8H), 3.59 – 3.48 (m, 2H), 3.48 – 3.42 (m, 2H), 3.36 – 3.32 (m, 1H), 3.13 (t, *J* = 3.1 Hz, 1H), 2.93 (d, *J* = 3.6 Hz, 1H), 2.08 (dt, *J* = 8.6, 5.0 Hz, 1H), 2.04 – 1.92 (m, 1H), 1.77 – 1.71 (m, 2H) ppm. ^{13}C NMR (101 MHz, CD_3OD) δ 140.4, 140.1, 140.1, 140.0, 129.4, 129.4, 129.3, 129.3, 129.2, 129.0, 128.9, 128.7, 128.7, 128.7, 128.6, 128.4, 102.2, 82.5, 81.6, 78.8, 78.6, 75.9, 75.5, 75.3, 74.8, 74.7, 74.6, 73.7, 72.1, 69.6, 69.4, 69.0, 68.3, 62.6, 55.9, 55.2, 44.3, 38.6, 27.9 ppm. HRMS (ESI) m/z: $[M+Na]^+$ calc for $C_{55}H_{64}O_{14}$ 971.41883, found 971.42001.

Compound 3

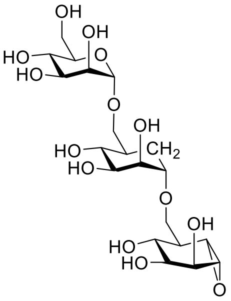

Compound **S11** (15 mg, 16 µmol) was dissolved in a mixture of MeOH/dioxane/H_2O (1:1:1, v/v/v, 1 mL) under nitrogen, and $Pd(OH)_2/C$ (20 wt%, 10 mg, 14 µmol) was added. The mixture was purged with H_2 gas and stirred vigorously for 6 h. Filtration over celite afforded the title compound as a white solid (6.7 mg, 85%). ^1H NMR (500 MHz, D_2O) δ 4.83 – 4.81 (m, 1H), 4.40 (t, *J* = 2.7 Hz, 1H), 4.08 (s, 1H), 3.93 (dd, *J* = 3.2, 1.6 Hz, 1H), 3.89 – 3.83 (m, 1H), 3.82 – 3.69 (m, 6H), 3.68 – 3.60 (m, 3H), 3.60 – 3.48 (m, 4H), 3.38 (t, *J* = 3.0 Hz, 1H), 3.29 (d, *J* = 3.6 Hz, 1H), 2.12 – 2.07 (m, 1H), 2.01 – 1.95 (m, 1H), 1.94 – 1.85 (m, 1H), 1.65 (t, *J* = 12.7 Hz, 1H) ppm. ^{13}C NMR (125 MHz, D_2O) δ 99.6, 77.3, 72.8, 72.7, 70.8, 70.6, 70.3, 70.1, 70.0, 67.9, 67.5, 67.2, 66.8, 65.9, 60.9, 55.9, 55.1, 42.7, 37.0, 25.9 ppm. HRMS (ESI) m/z: $[M+Na]^+$ calc for $C_{20}H_{34}O_{14}$ 521.18408, found 521.18500.

4. NMR spectra

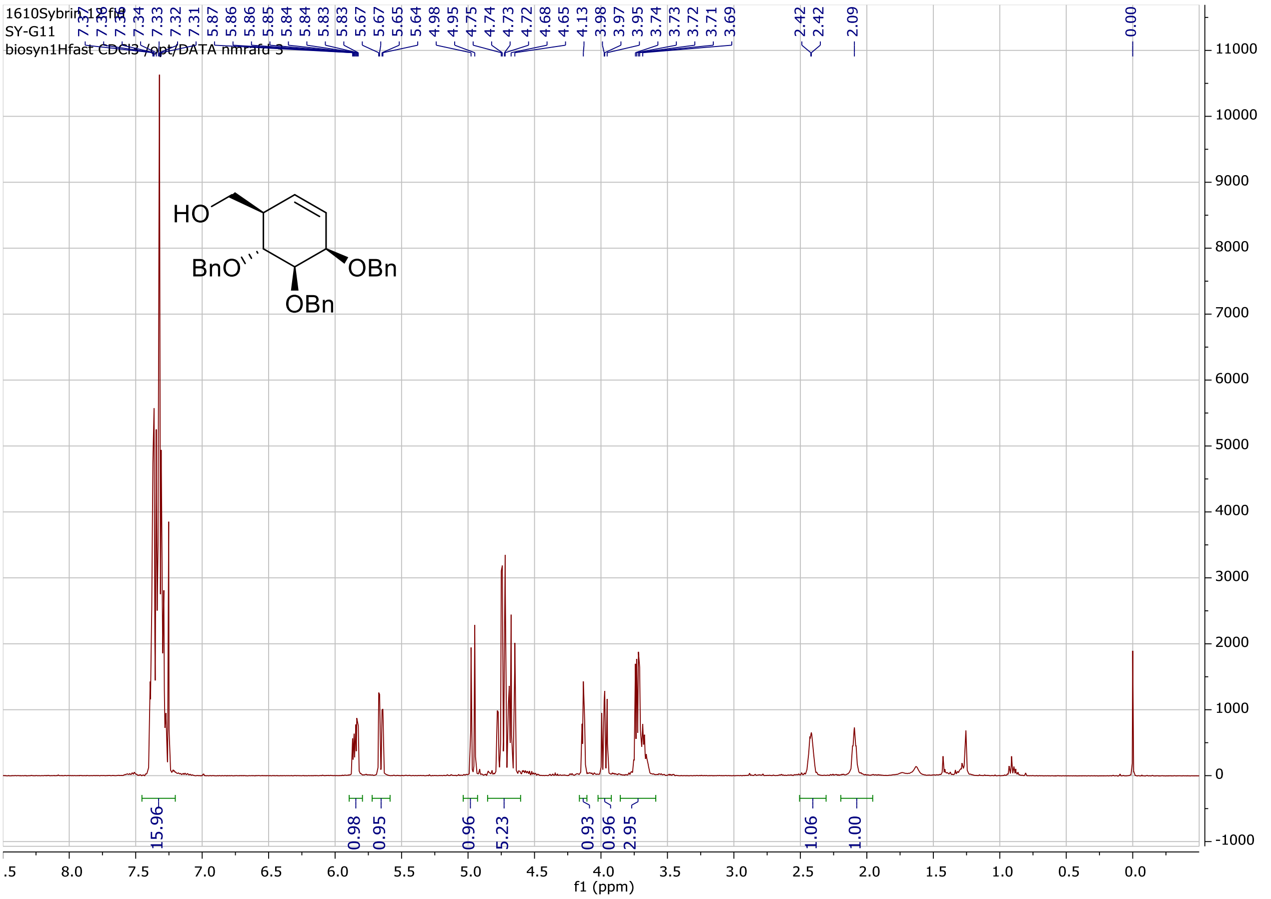

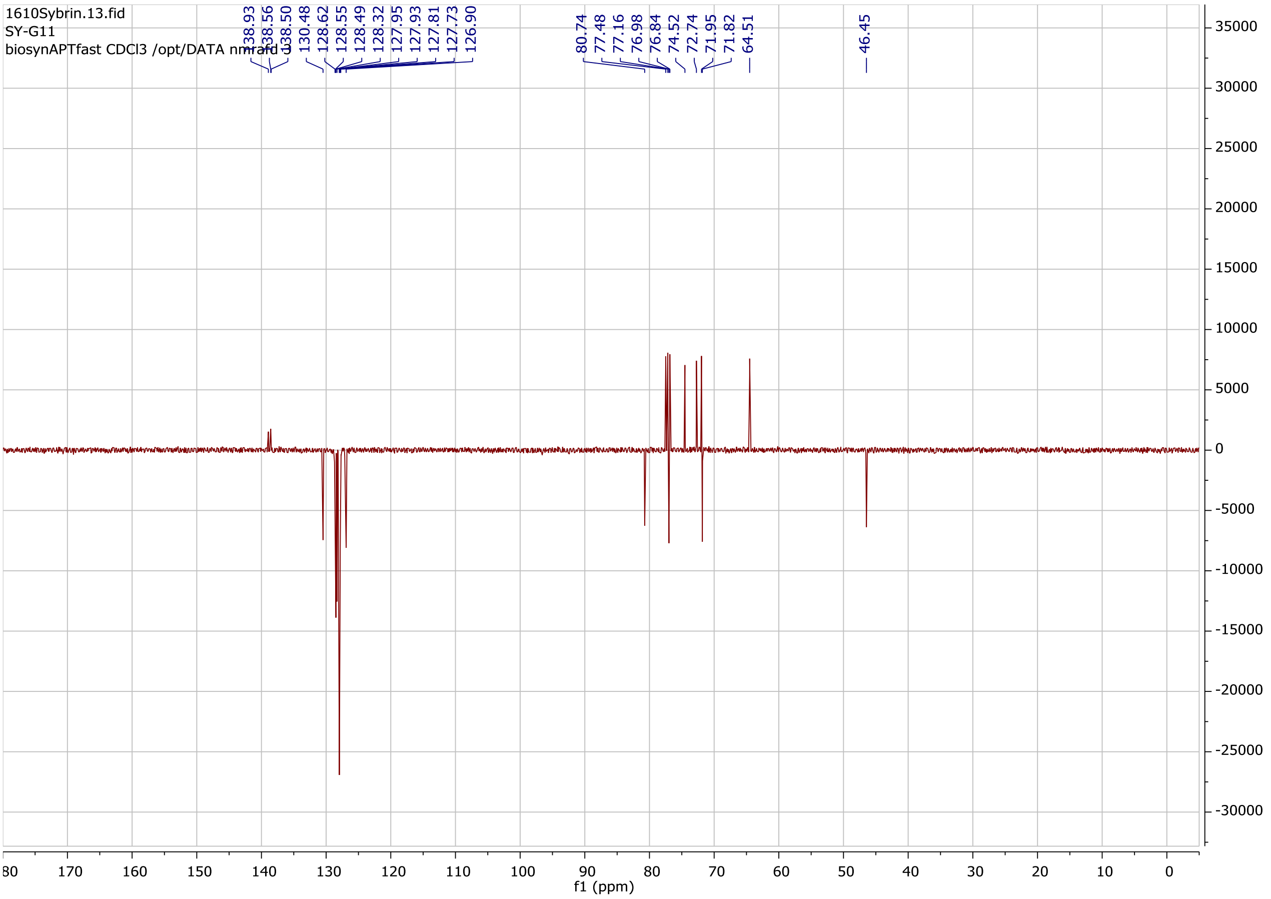

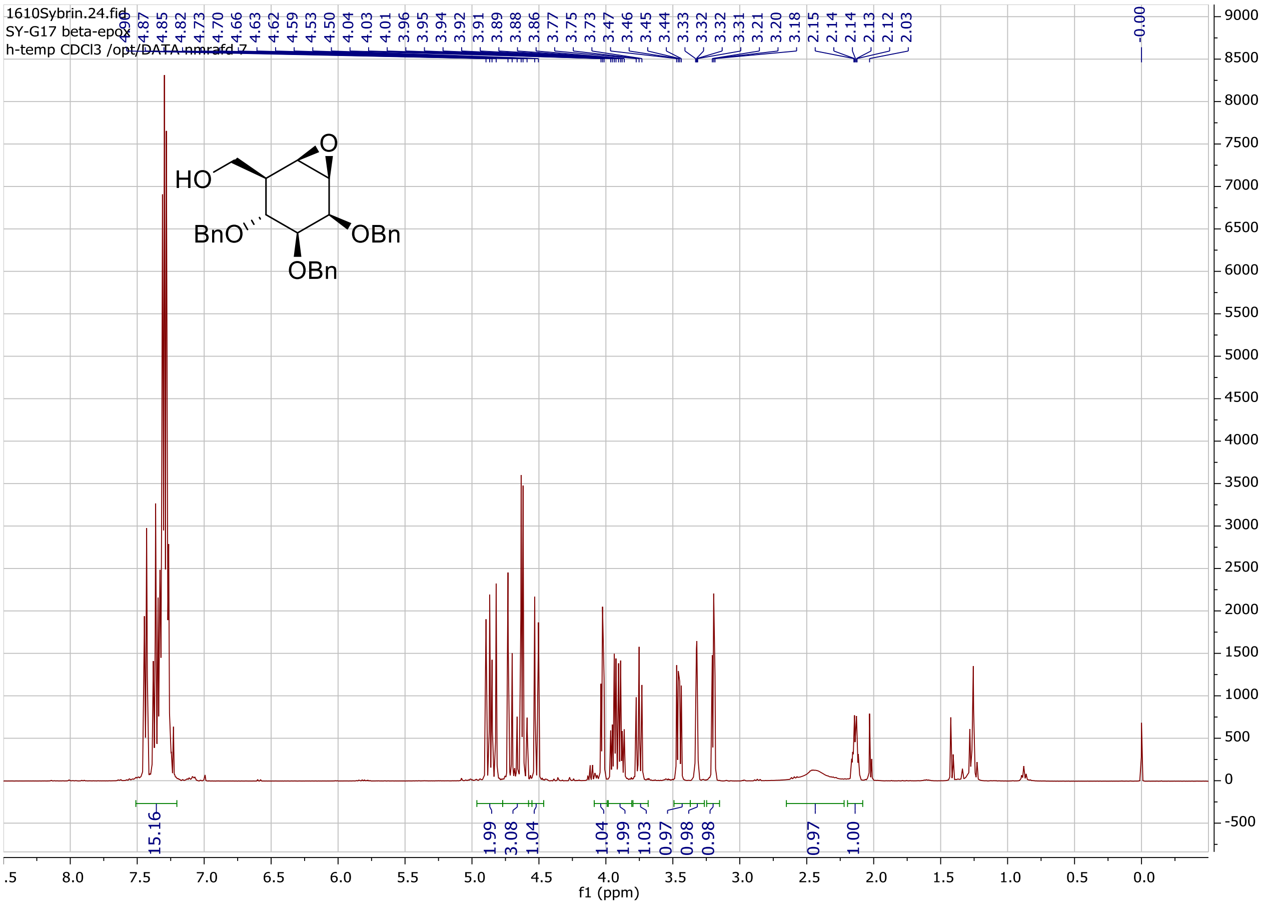
1610Sybrin.24.fid
SY-G17 beta-epox
h-temp CDCl3 /opt/DATA nmrafd 7
f1 (ppm)

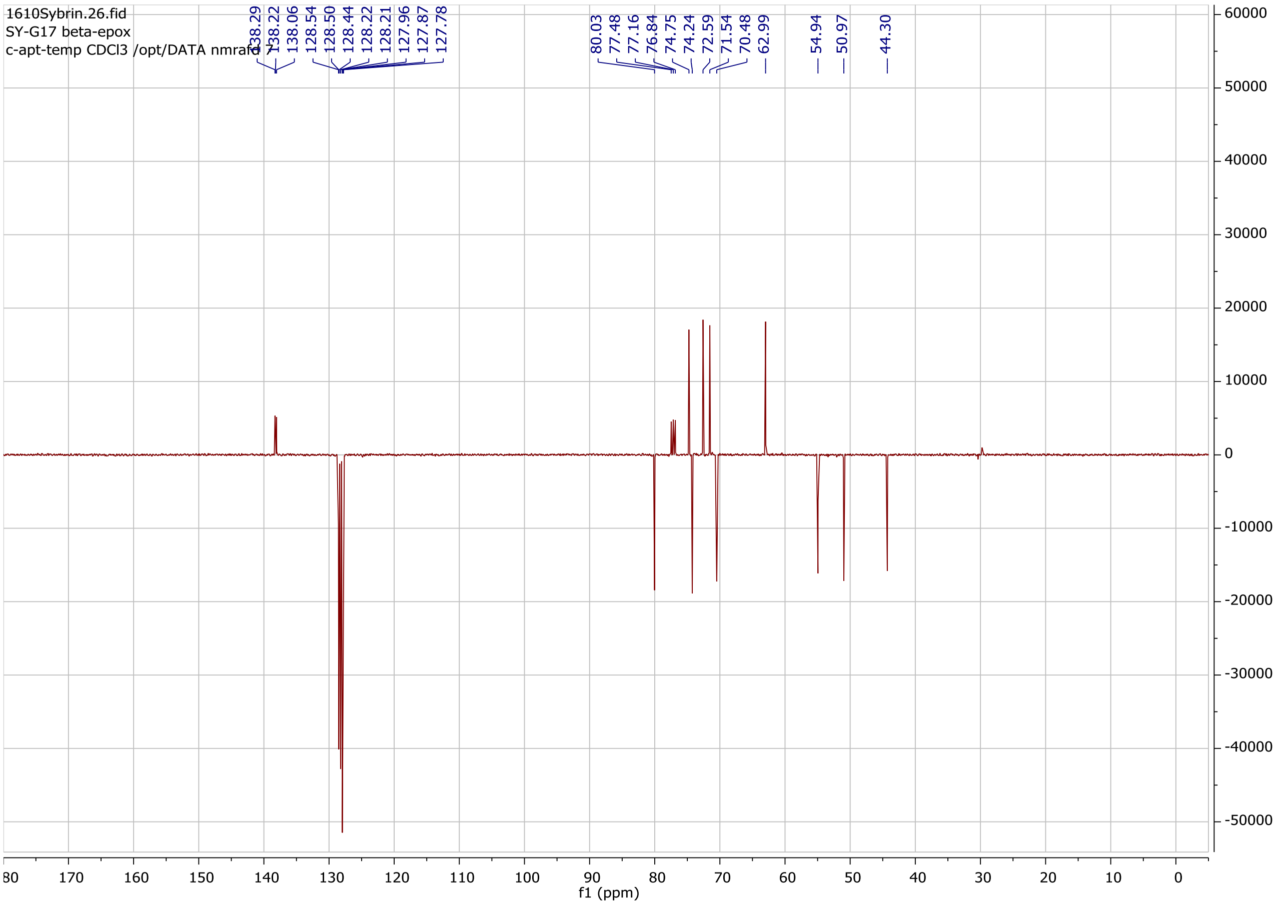
1610Sybrin.26.fid
SY-G17 beta-epox
c-apt-temp CDCl3 /opt/DATA nmrafd 7
f1 (ppm)

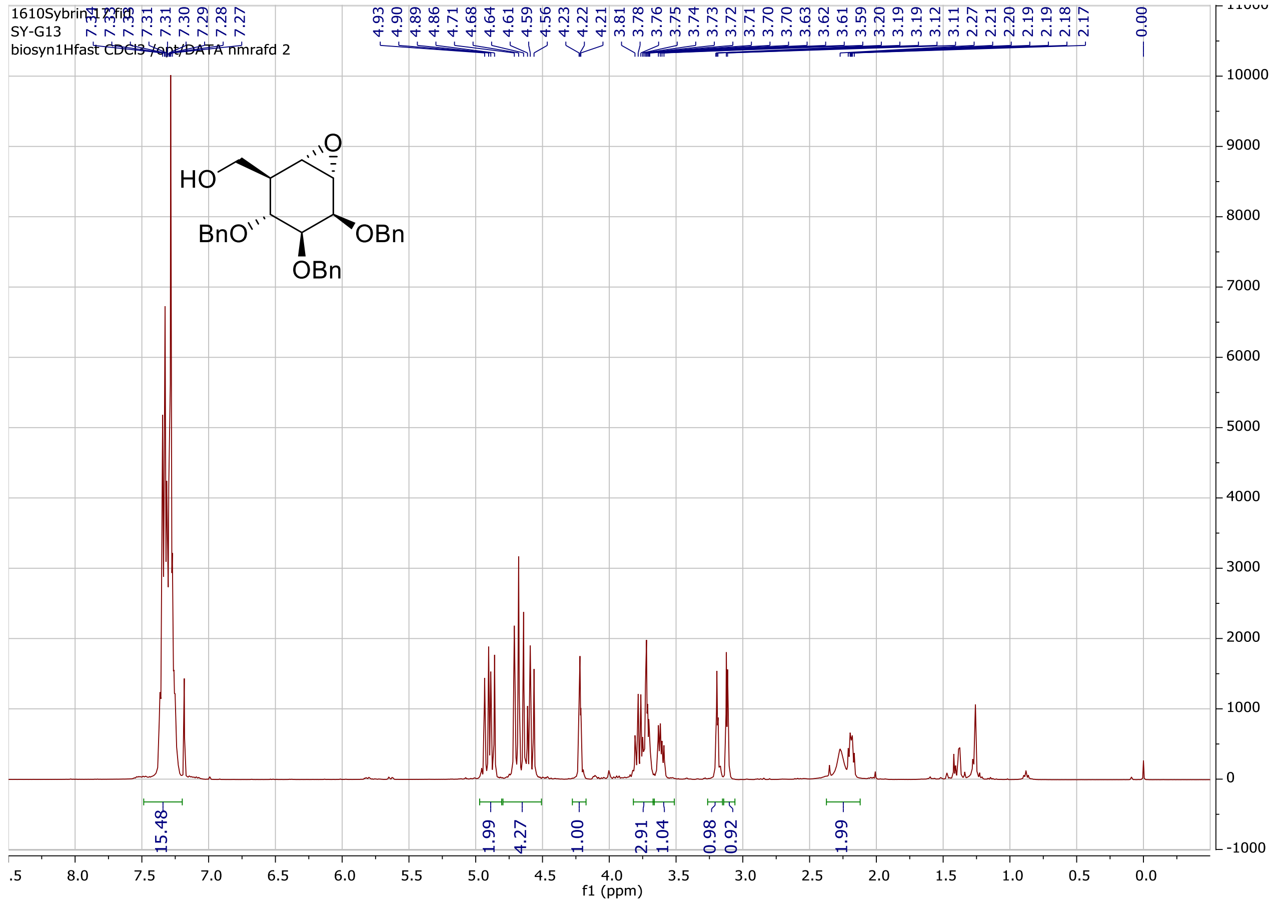
1610Sybrin.17.fid
SY-G13
biosyn1Hfast CDCl3 /opt/DATA nmrafd 2
HO
O
BnO
OBn
OBn
15.48
1.99
4.27
1.00
2.91
1.04
0.98
0.92
1.99
f1 (ppm)

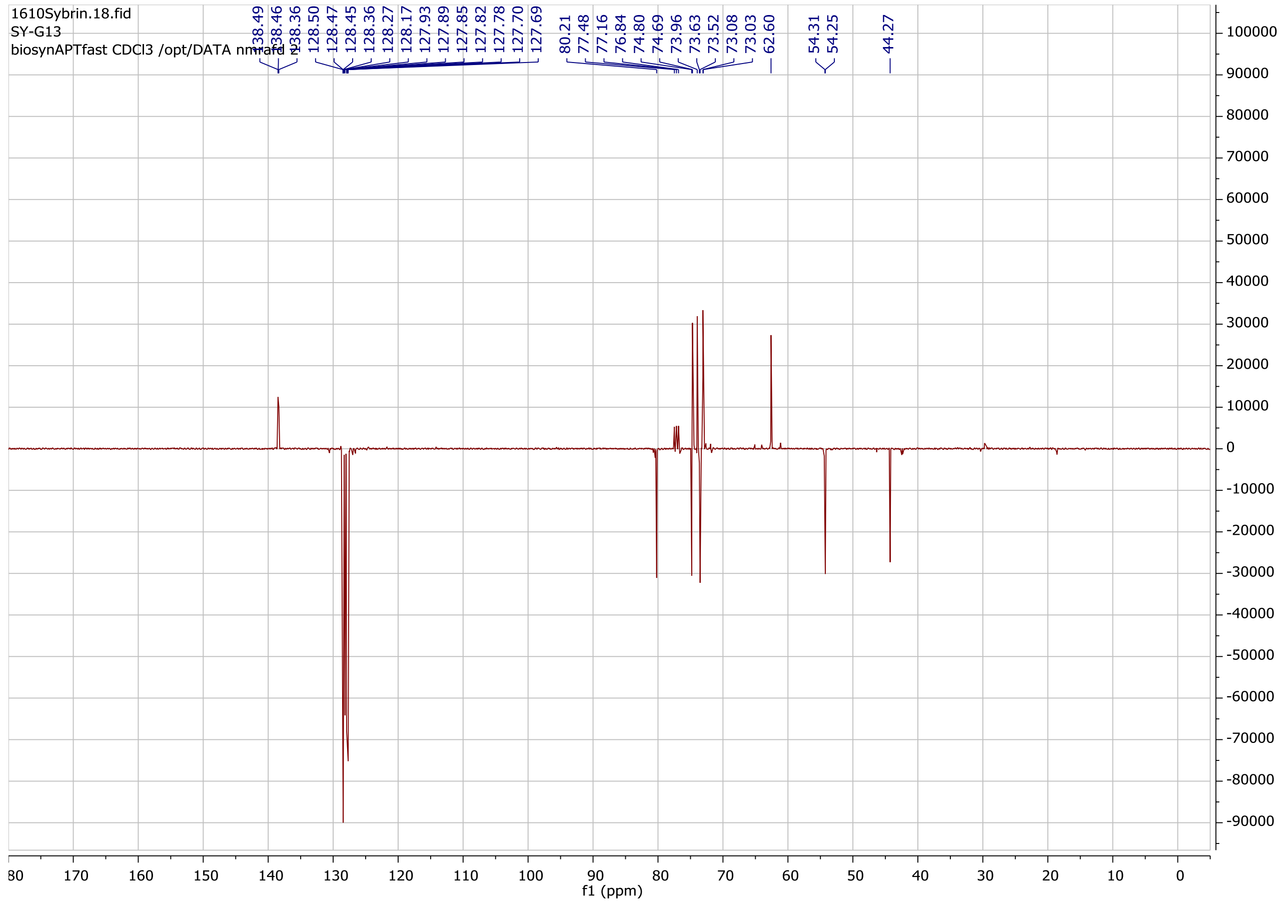
1610Sybrin.18.fid
SY-G13
biosynAPTfast CDCl3 /opt/DATA nmrafd 2
80.21
77.48
77.16
76.84
74.80
74.69
73.96
73.63
73.52
73.08
73.03
62.60
54.31
54.25
44.27
f1 (ppm)

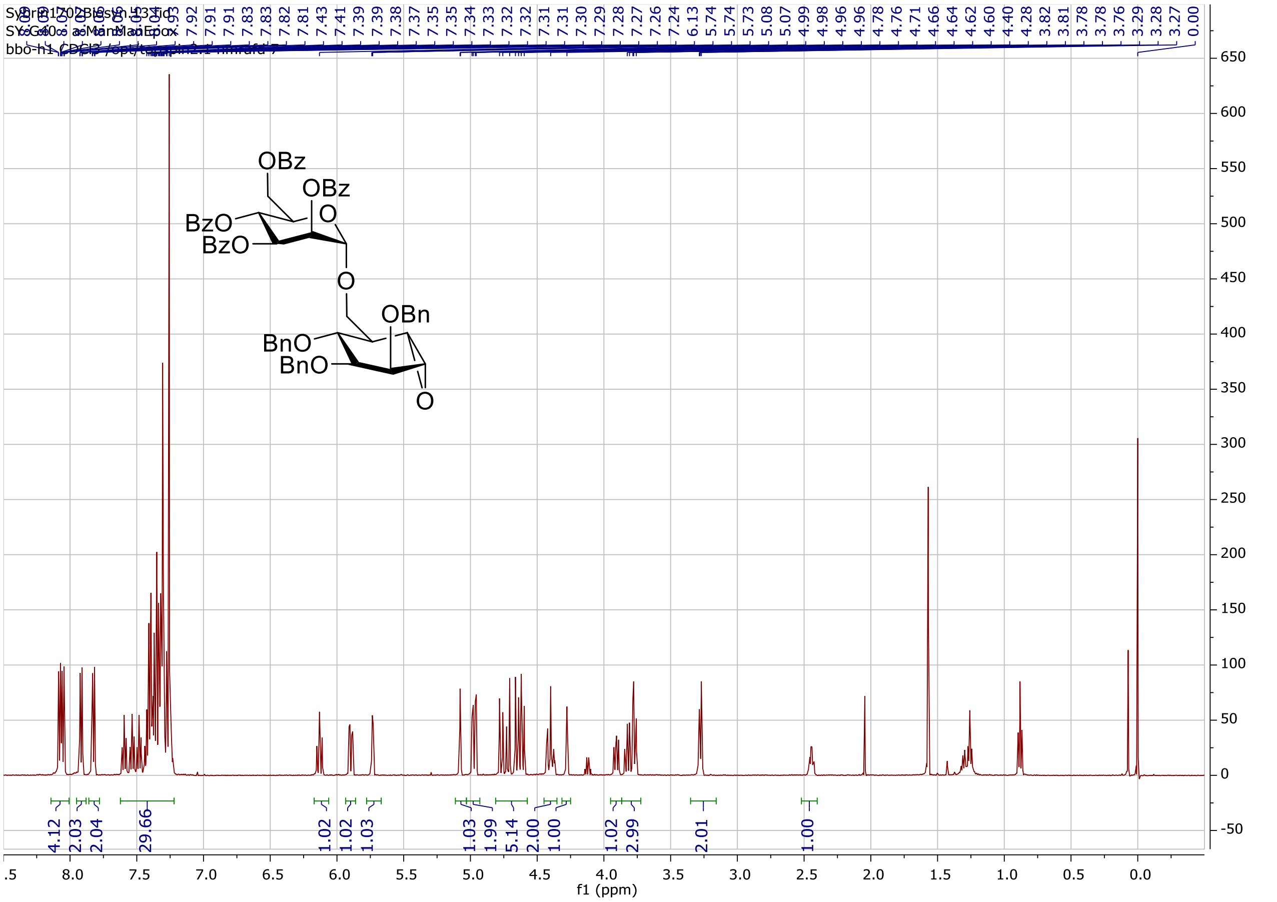
OBz
OBz
BzO
BzO
O
O
OBn
BnO
BnO
O
7.92 7.91 7.91 7.83 7.82 7.81 7.43 7.41 7.39 7.39 7.38 7.37 7.35 7.35 7.34 7.33 7.32 7.32 7.31 7.31 7.30 7.29 7.28 7.27 7.26 7.24 6.13 5.74 5.74 5.73 5.08 5.07 4.99 4.98 4.96 4.96 4.78 4.76 4.71 4.66 4.64 4.62 4.60 4.40 4.28 3.82 3.81 3.78 3.78 3.76 3.29 3.28 3.27 0.00
4.12 2.03 2.04 29.66 1.02 1.02 1.03 1.03 1.99 5.14 2.00 1.00 1.02 2.99 2.01 1.00
f1 (ppm)

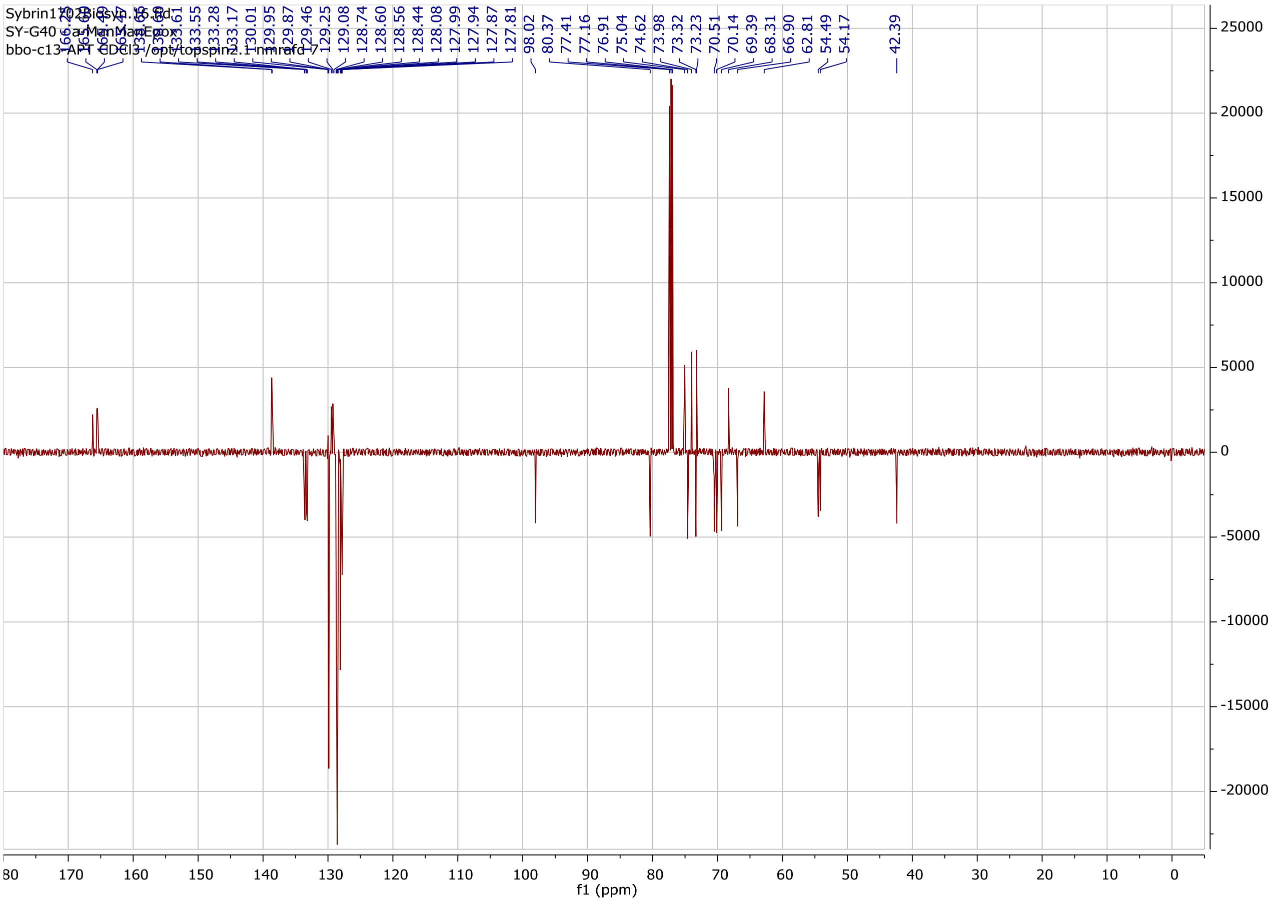
SY-G40 a-ManManEpox
133.55 133.28 133.17 130.01 129.95 129.87 129.46 129.25 129.08 128.74 128.60 128.56 128.44 128.08 127.99 127.94 127.87 127.81 98.02 80.37 77.41 77.16 76.91 75.04 74.62 73.98 73.32 73.23 70.51 70.14 69.39 68.31 66.90 62.81 54.49 54.17 42.39
f1 (ppm)

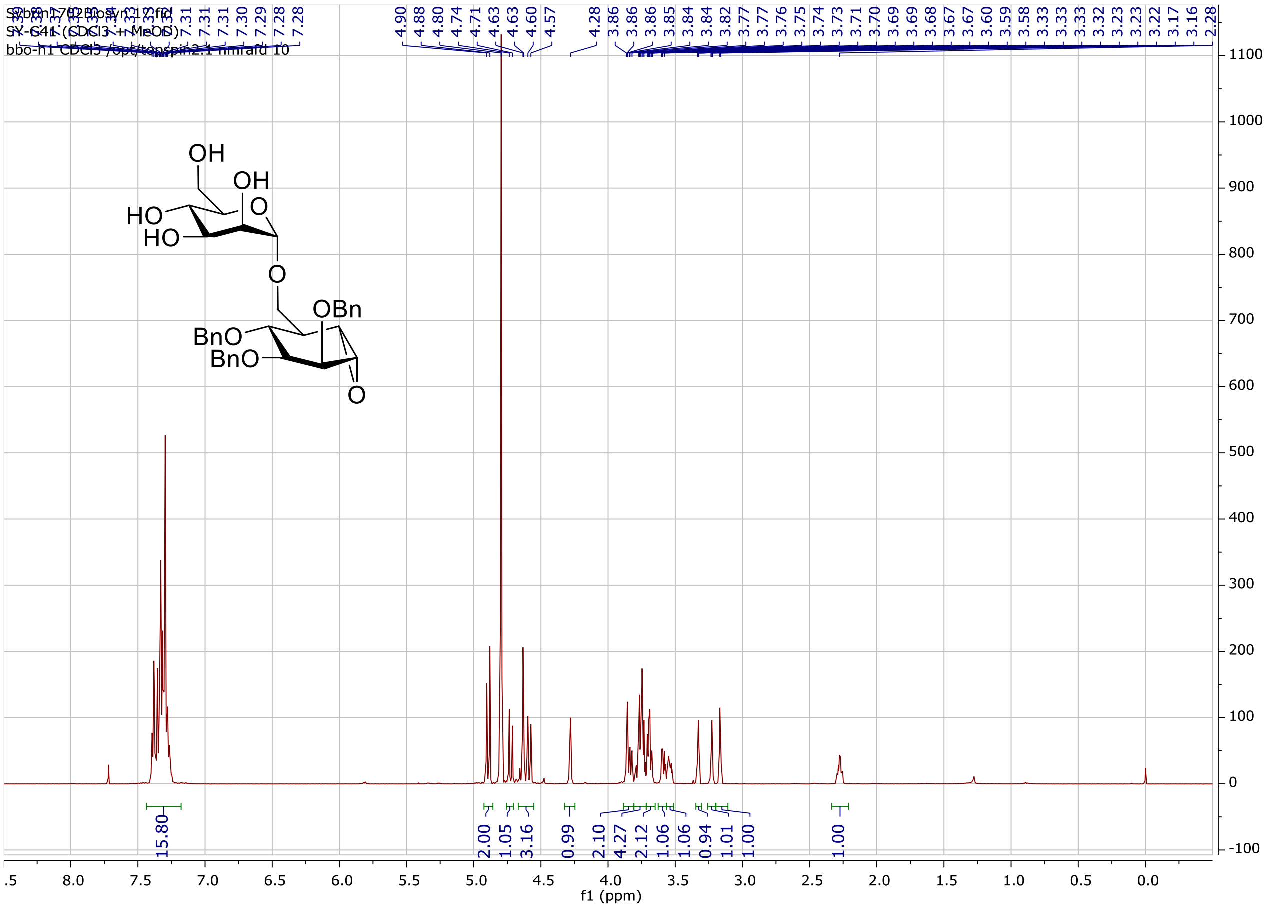
SY-G41 (CDCl3 + MeOD)
bbo-h1 CDCl3 /opt/topspin2.1 nmrafd 10
7.31
7.31
7.31
7.30
7.29
7.28
7.28
4.90
4.88
4.80
4.74
4.71
4.63
4.63
4.60
4.57
4.28
3.86
3.86
3.85
3.84
3.84
3.82
3.77
3.77
3.76
3.75
3.74
3.73
3.71
3.70
3.69
3.69
3.68
3.67
3.67
3.60
3.59
3.58
3.33
3.33
3.32
3.23
3.23
3.22
3.17
3.16
2.28
OH
OH
HO
HO
O
O
OBn
BnO
BnO
O
15.80
2.00
1.05
3.16
0.99
2.10
4.27
2.12
1.06
1.06
0.94
1.01
1.00
1.00
f1 (ppm)

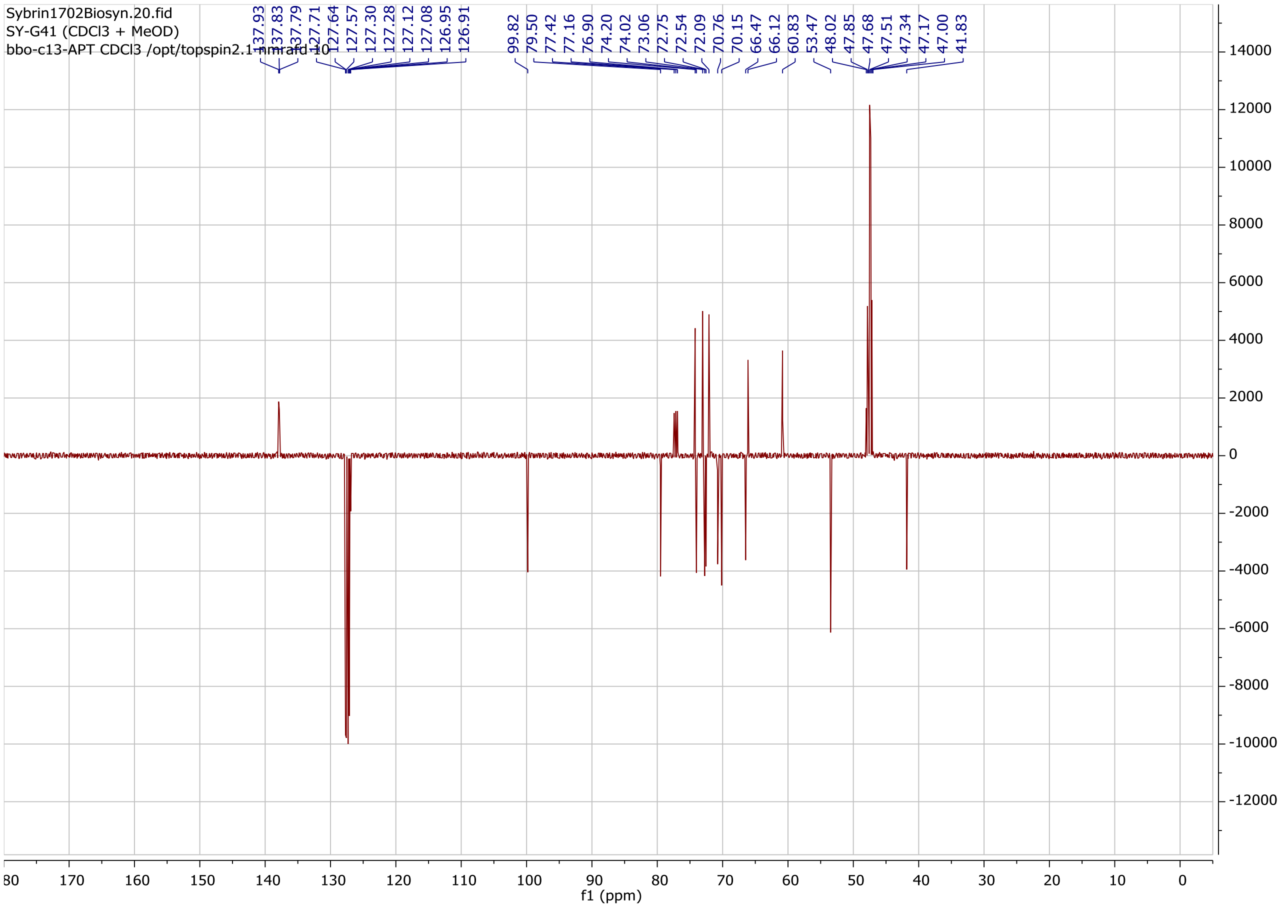
Sybrin1702Biosyn.20.fid
SY-G41 (CDCl3 + MeOD)
bbo-c13-APT CDCl3 /opt/topspin2.1 nmrafd 10
137.93
137.83
137.79
127.71
127.64
127.57
127.30
127.28
127.12
127.08
126.95
126.91
99.82
79.50
77.42
77.16
76.90
74.20
74.02
73.06
72.75
72.54
72.09
70.76
70.15
66.47
66.12
60.83
53.47
48.02
47.85
47.68
47.51
47.34
47.17
47.00
41.83
f1 (ppm)

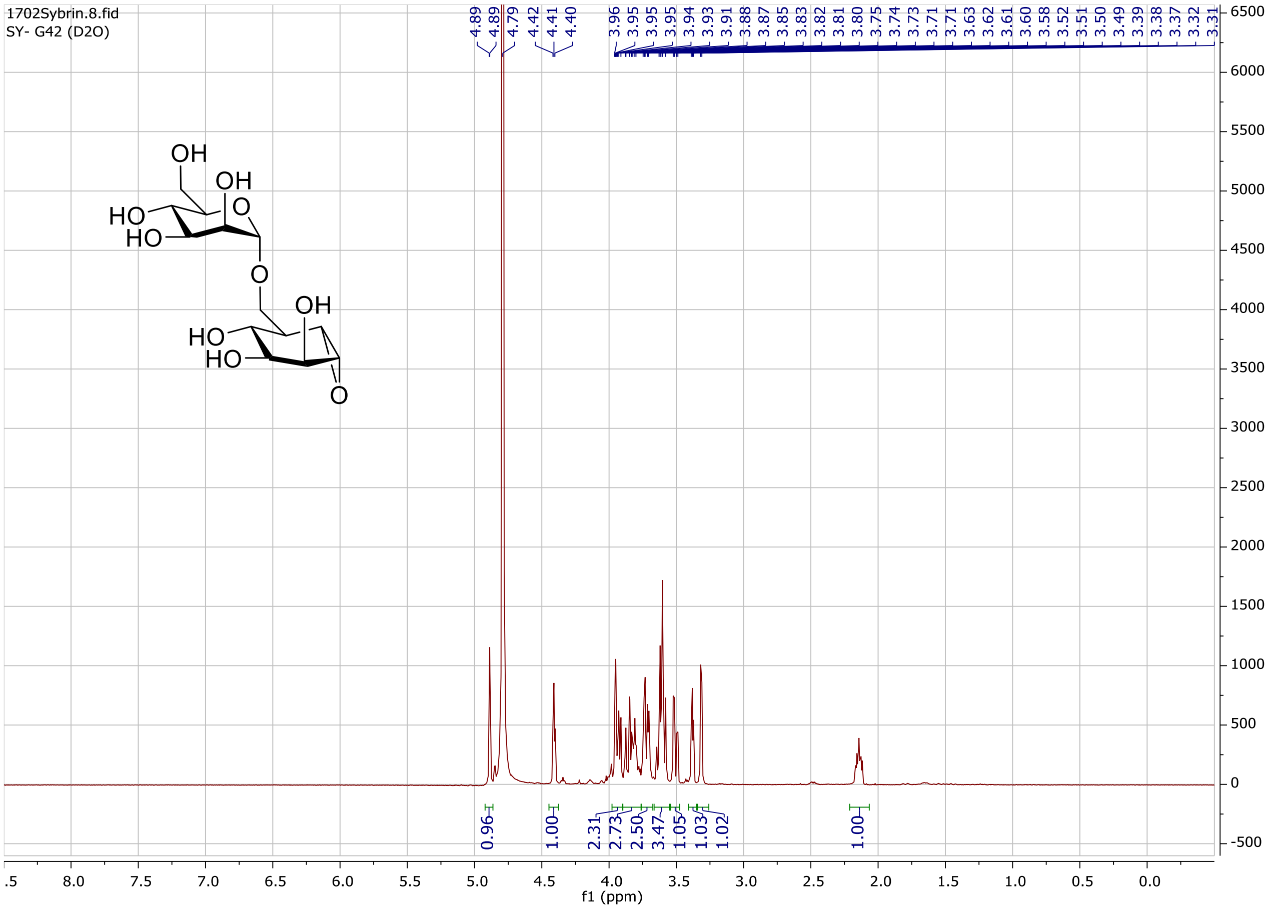
1702Sybrin.8.fid
SY- G42 (D2O)
f1 (ppm)

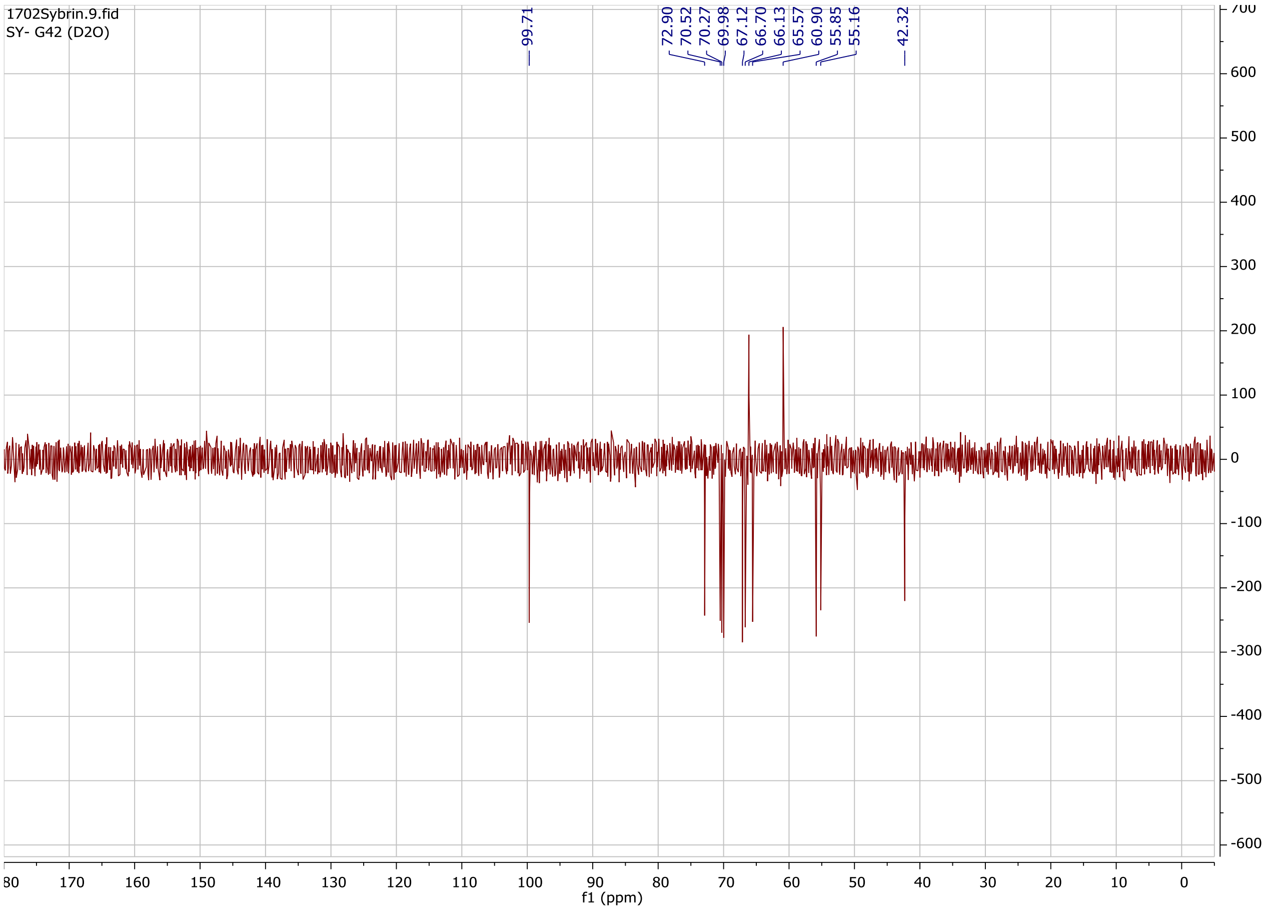
1702Sybrin.9.fid
SY- G42 (D2O)
f1 (ppm)

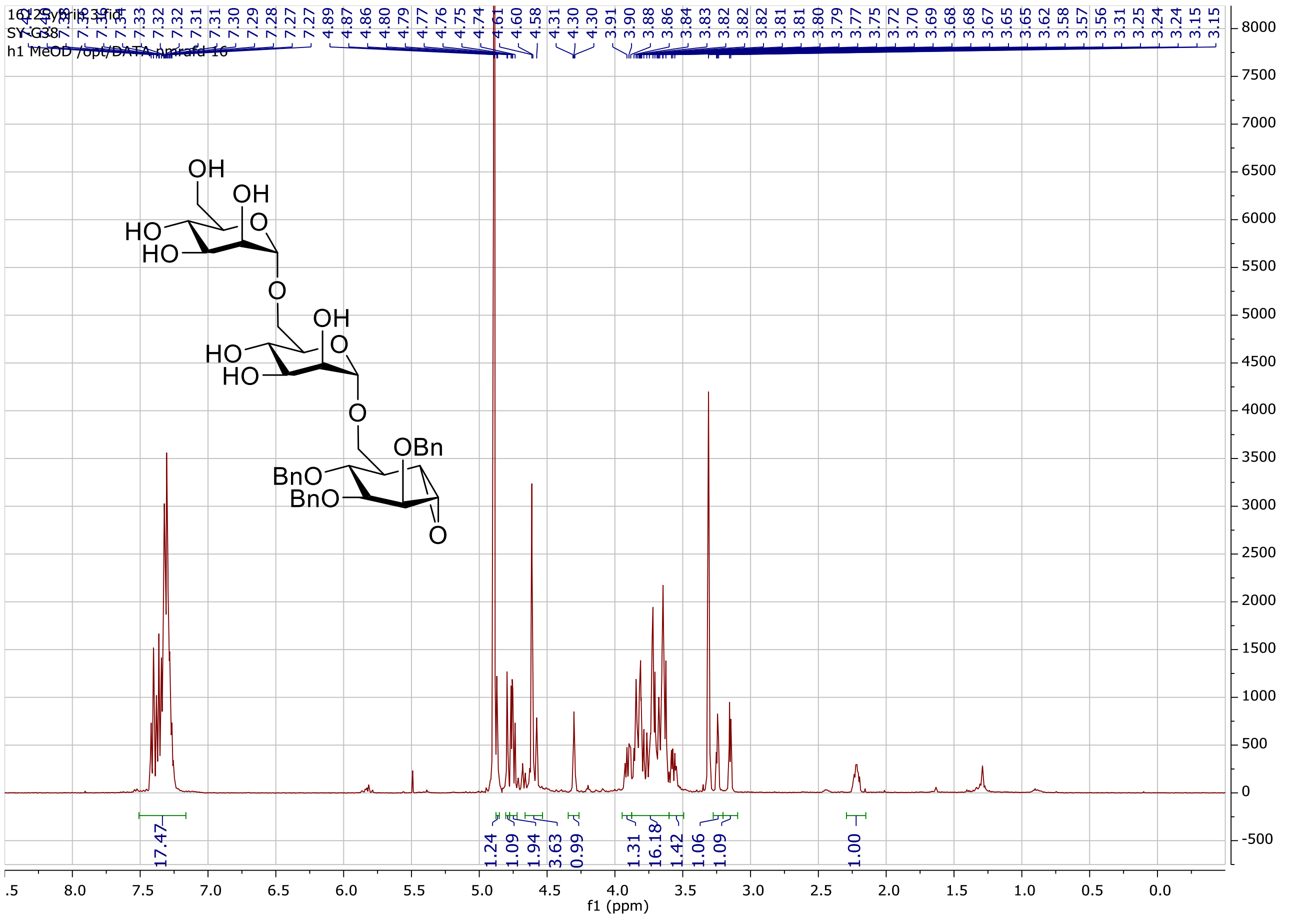
SY-G38
h1 MeOD /opt/DATA nmrafd-16
7.33 7.32 7.32 7.31 7.31 7.30 7.29 7.28 7.27 7.27 4.89 4.87 4.86 4.80 4.79 4.77 4.76 4.75 4.74 4.61 4.60 4.58 4.31 4.30 4.30 3.91 3.90 3.88 3.86 3.84 3.83 3.82 3.82 3.82 3.81 3.81 3.80 3.79 3.77 3.75 3.72 3.70 3.69 3.68 3.68 3.67 3.65 3.65 3.62 3.58 3.57 3.56 3.31 3.25 3.24 3.15 3.15
OH
OH
O
HO
HO
O
OH
O
HO
HO
O
OBn
BnO
BnO
O
17.47
1.24 1.09 1.94 3.63 0.99 1.31 16.18 1.42 1.06 1.09 1.00
f1 (ppm)
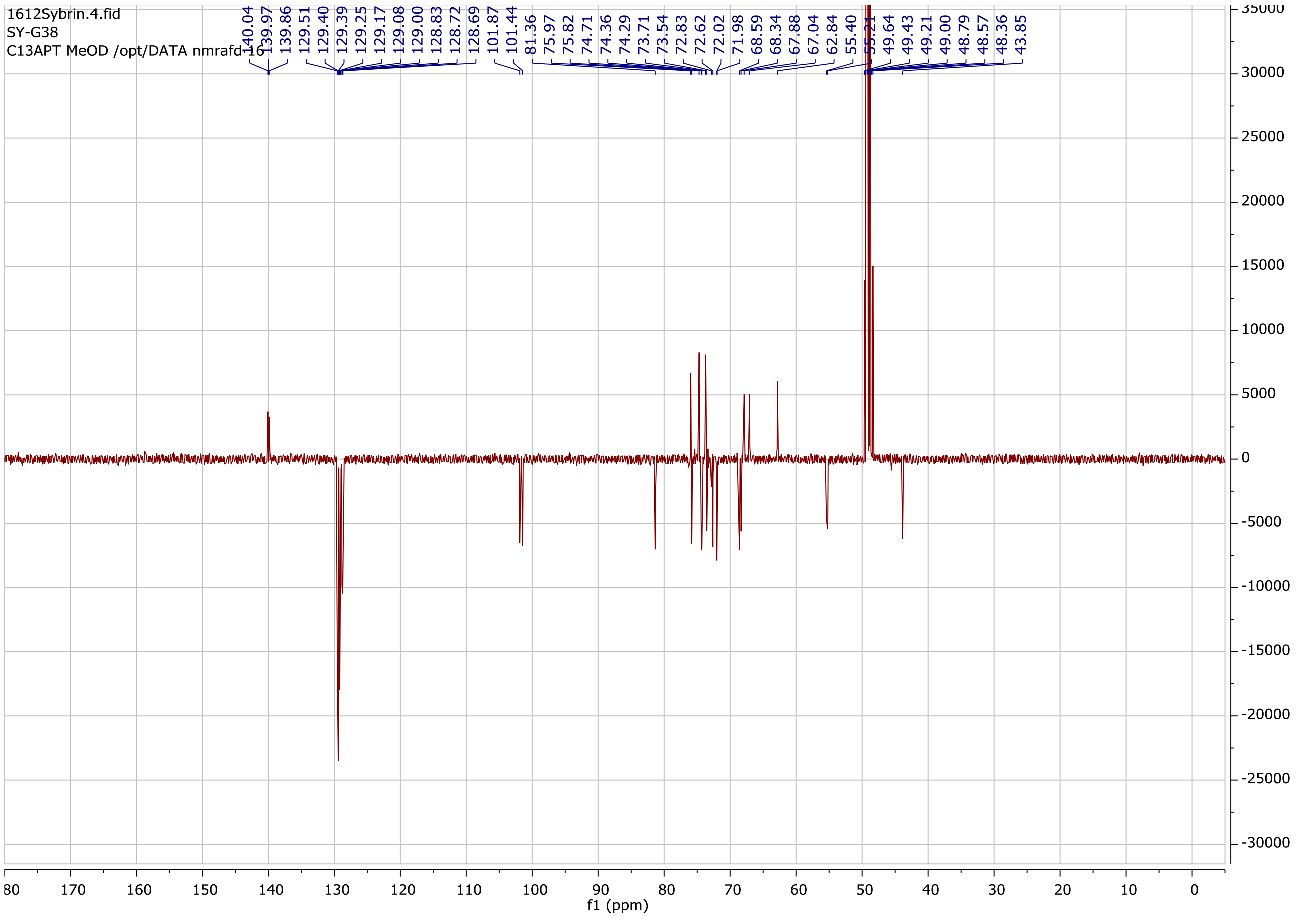
1612Sybrin.4.fid
SY-G38
C13APT MeOD /opt/DATA nmrafd-16
140.04 139.97 139.86 129.51 129.40 129.39 129.25 129.17 129.08 129.00 128.83 128.72 128.69 101.87 101.44 81.36 75.97 75.82 74.71 74.36 74.29 73.71 73.54 72.83 72.62 72.02 71.98 68.59 68.34 67.88 67.04 62.84 55.40 55.21 49.64 49.43 49.21 49.00 48.79 48.57 48.36 43.85
f1 (ppm)

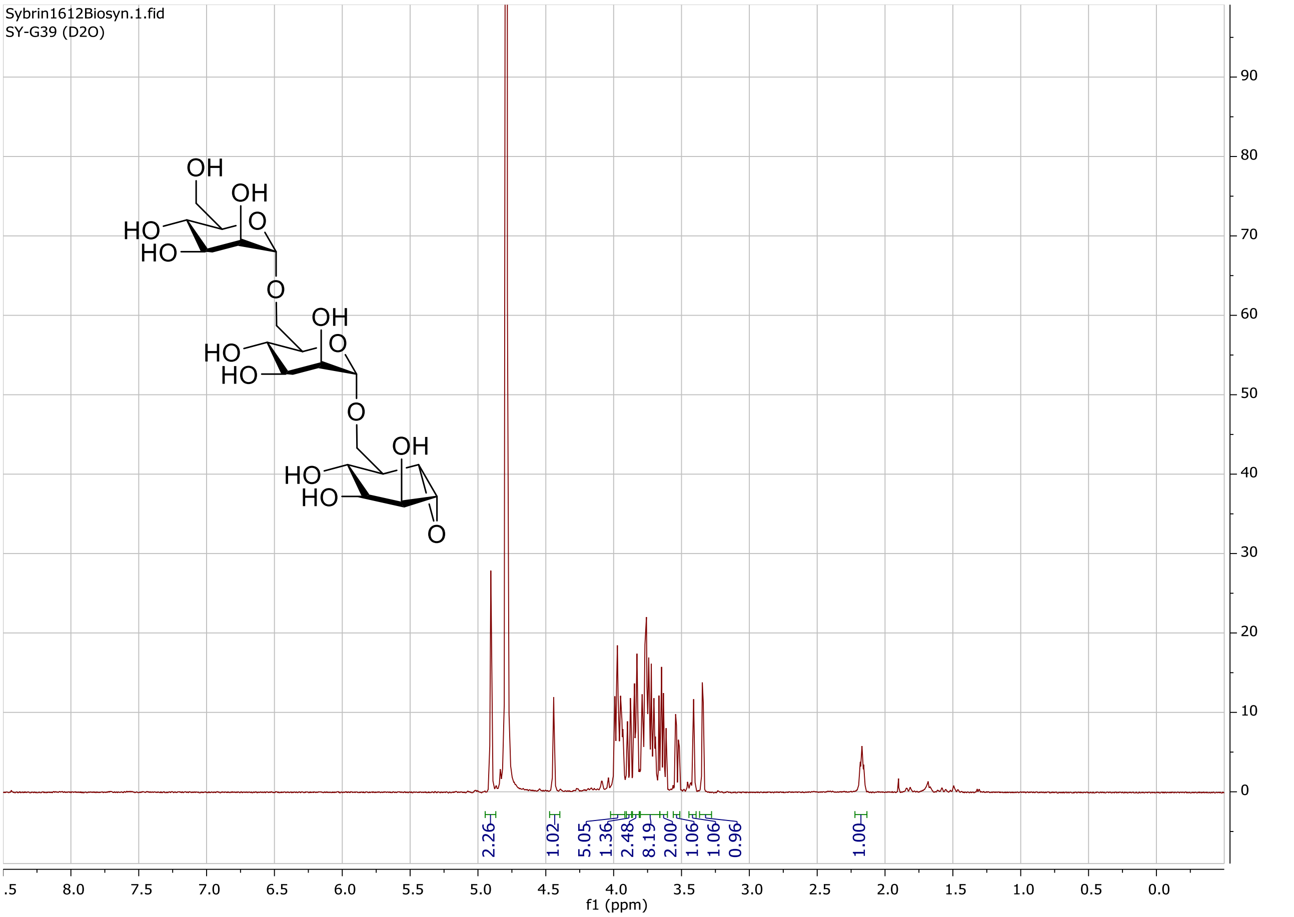
Sybrin1612Biosyn.1.fid
SY-G39 (D2O)
OH
OH
HO
O
HO
O
OH
HO
O
HO
O
OH
HO
HO
O
2.26
1.02
5.05
1.36
2.48
8.19
2.00
1.06
1.06
0.96
1.00
f1 (ppm)

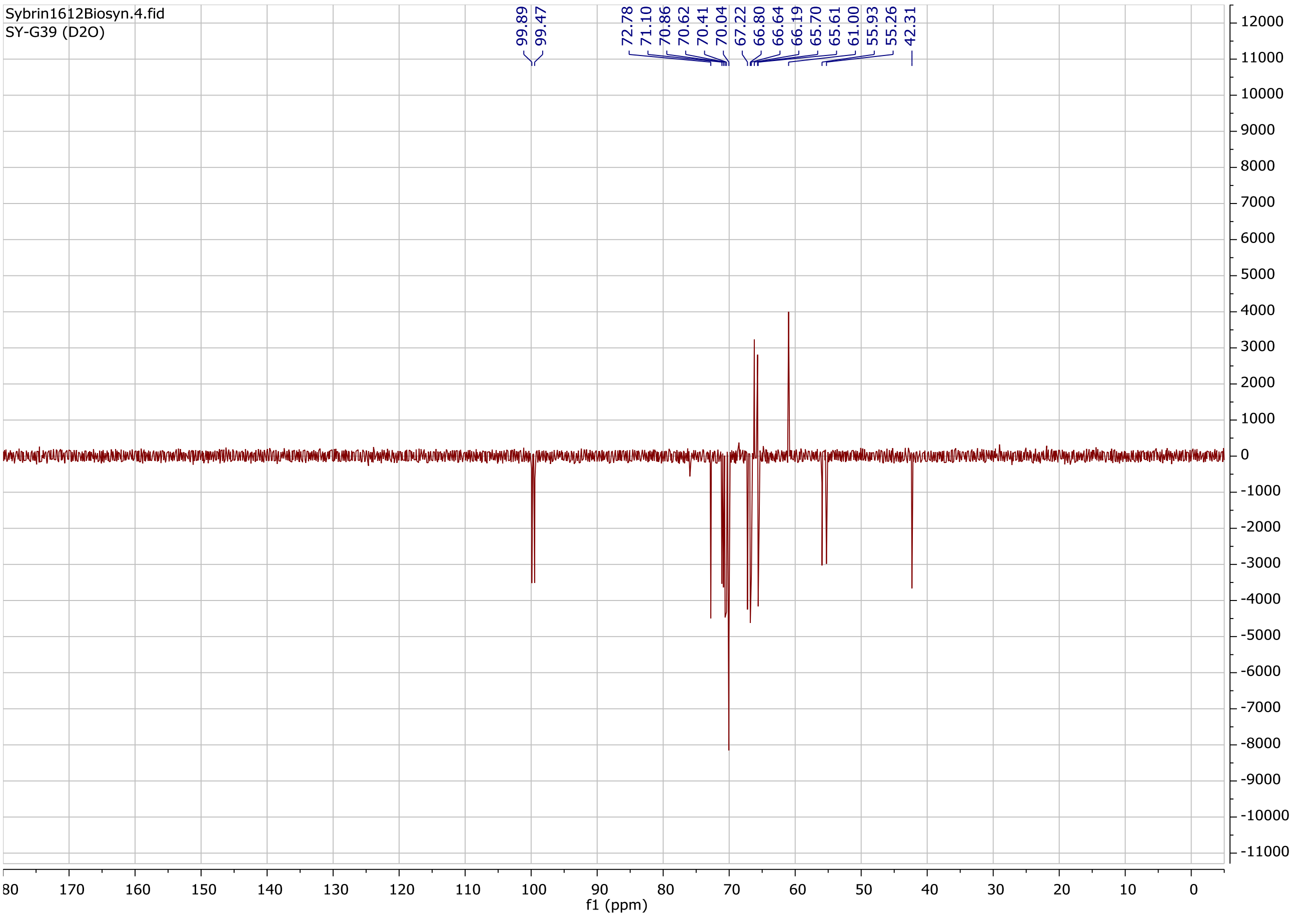
Sybrin1612Biosyn.4.fid
SY-G39 (D2O)
99.89
99.47
72.78
71.10
70.86
70.62
70.41
70.04
67.22
66.80
66.64
66.19
65.70
65.61
61.00
55.93
55.26
42.31
f1 (ppm)

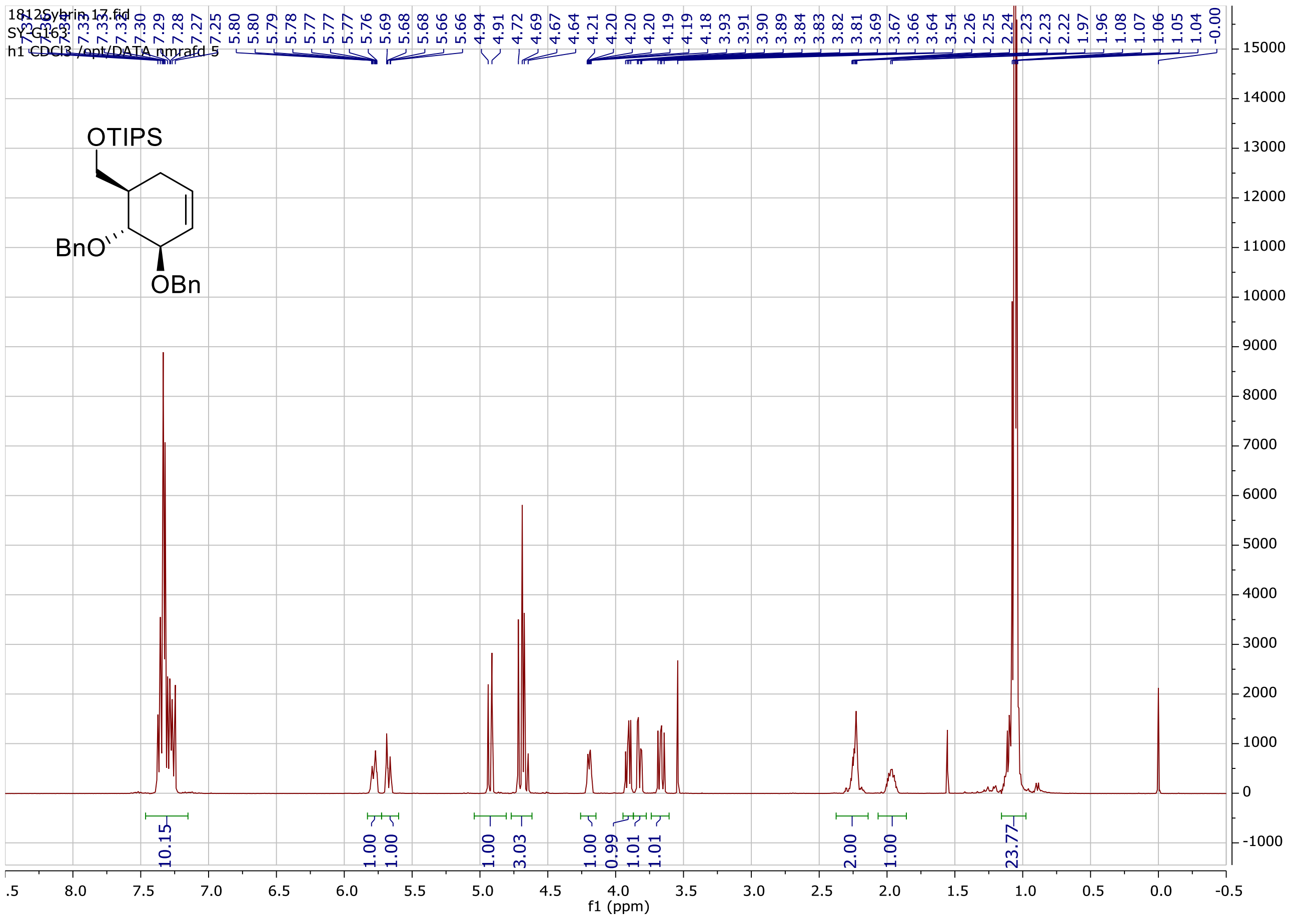
1812Sybrin.17.fid
SY-G163
h1 CDCl3 /opt/DATA nmrafd 5
OTIPS
BnO
OBn
f1 (ppm)

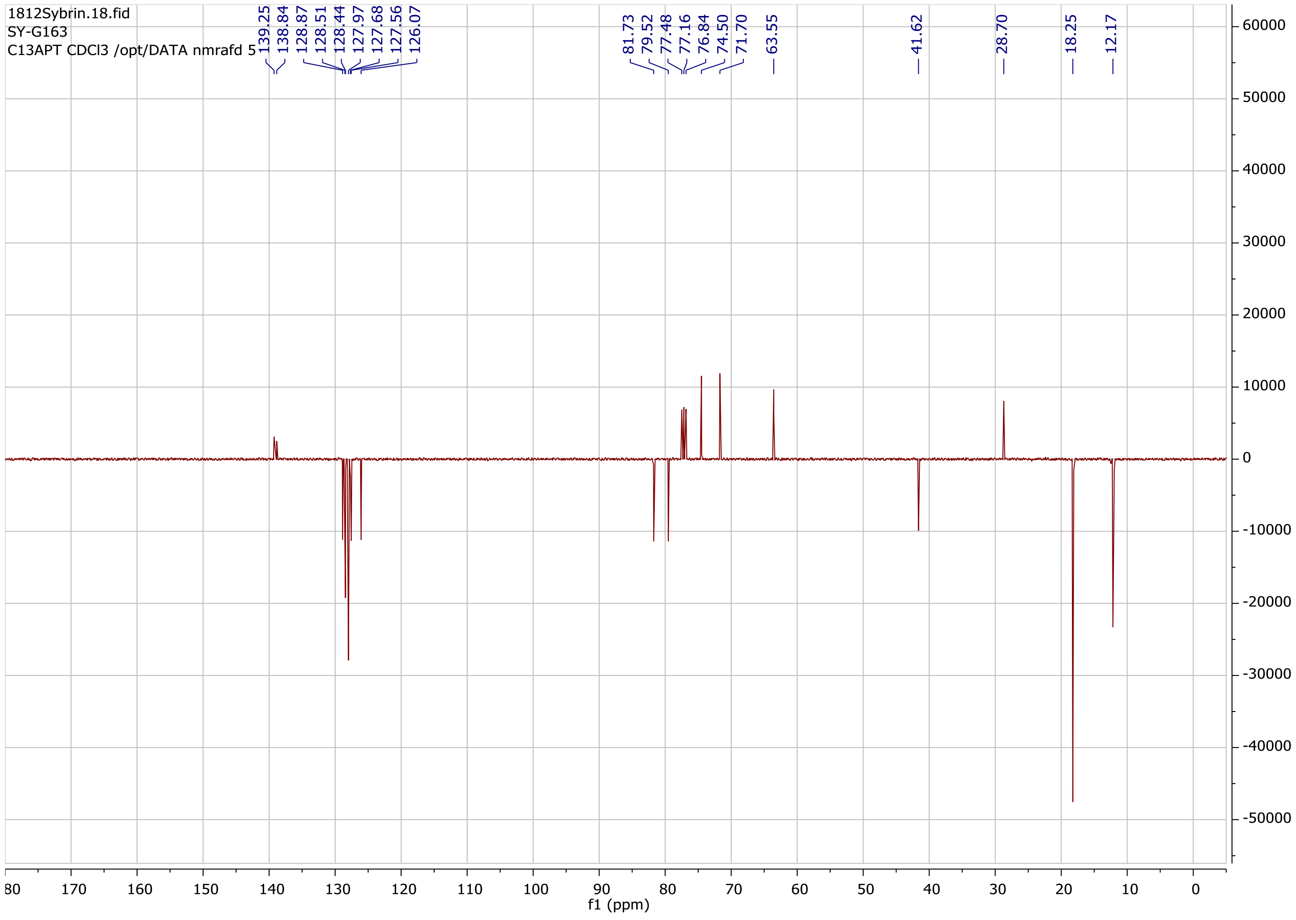
1812Sybrin.18.fid
SY-G163
C13APT CDCl3 /opt/DATA nmrafd 5
139.25
138.84
128.87
128.51
128.44
127.97
127.68
127.56
126.07
81.73
79.52
77.48
77.16
76.84
74.50
71.70
63.55
41.62
28.70
18.25
12.17
f1 (ppm)

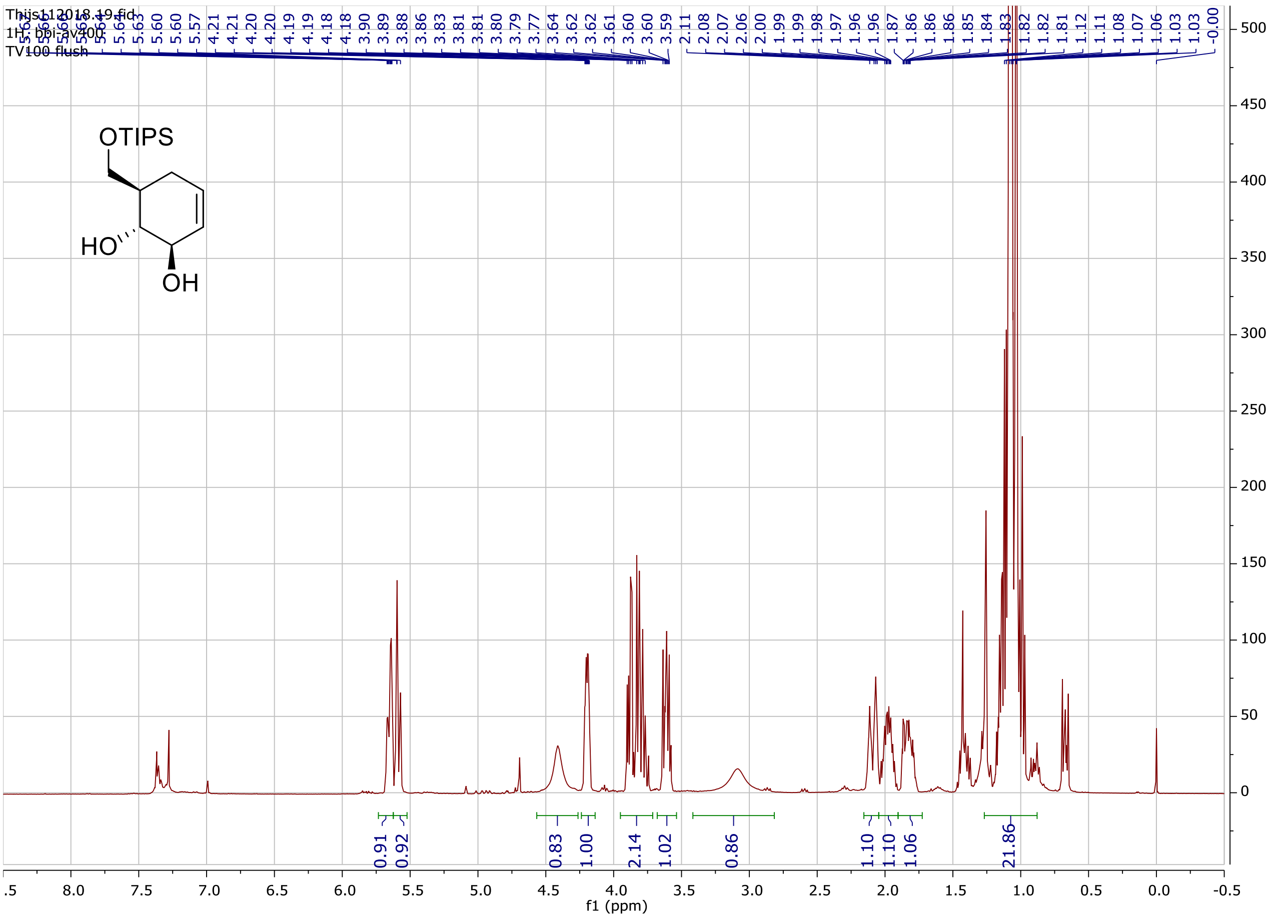
Thijs112018.19.fid
TV100 flush
OTIPS
HO
OH
0.91
0.92
0.83
1.00
2.14
1.02
0.86
1.10
1.10
1.06
21.86
f1 (ppm)

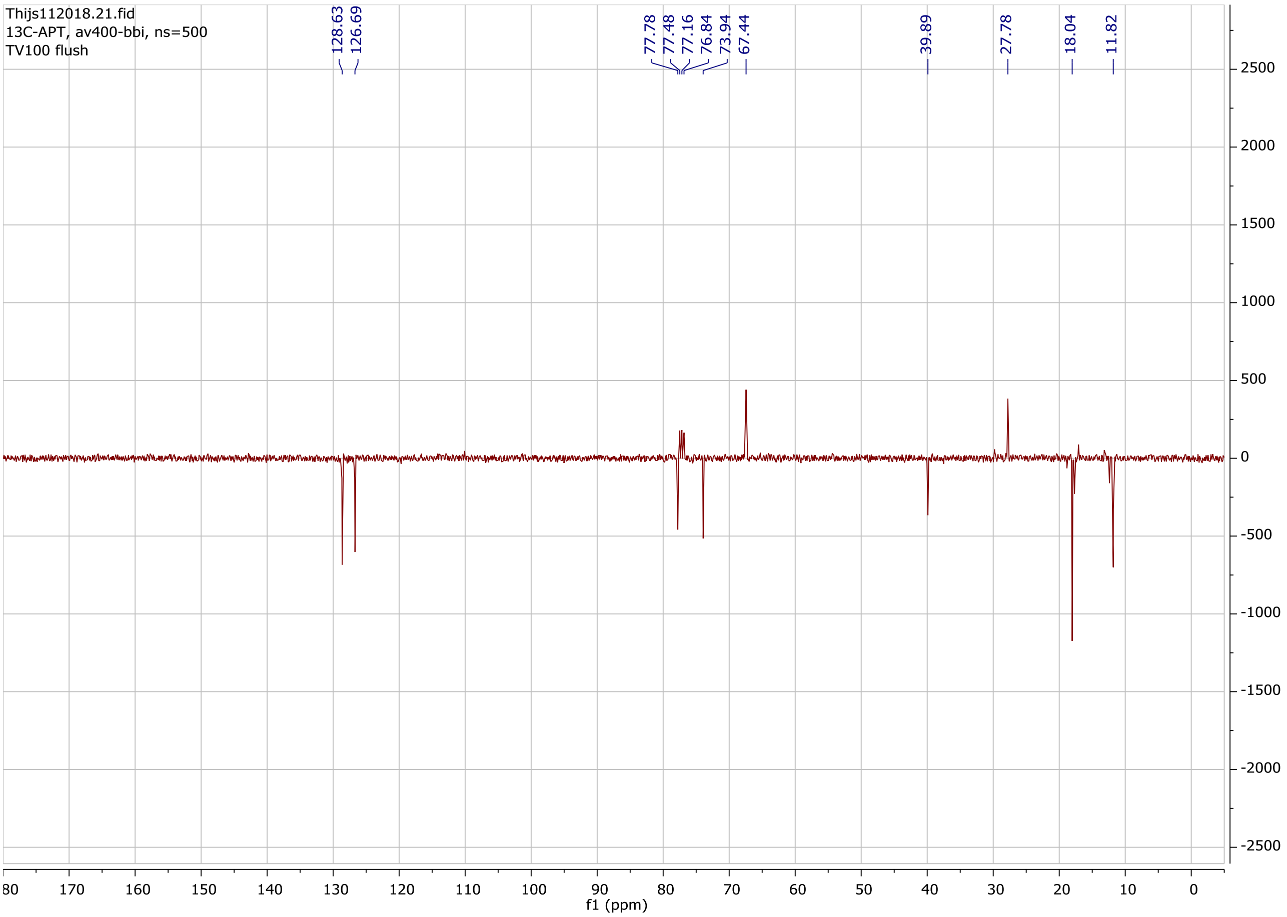
Thijs112018.21.fid
13C-APT, av400-bbi, ns=500
TV100 flush
128.63
126.69
77.78
77.48
77.16
76.84
73.94
67.44
39.89
27.78
18.04
11.82
f1 (ppm)

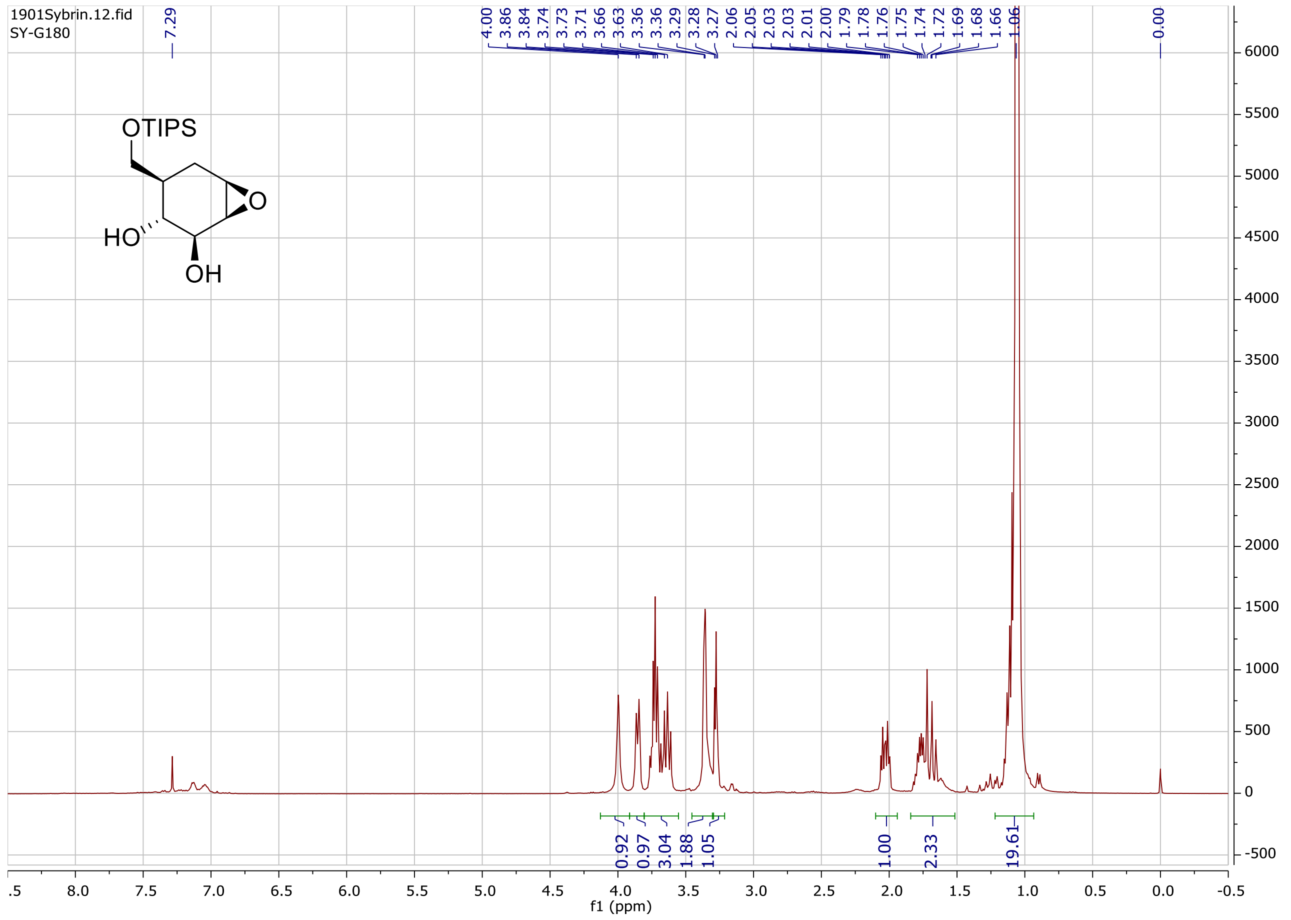
1901Sybrin.12.fid
SY-G180
OTIPS
HO
OH
O
7.29
4.00 3.86 3.84 3.74 3.73 3.71 3.66 3.63 3.36 3.36 3.29 3.28 3.27 2.06 2.05 2.03 2.03 2.01 2.00 1.79 1.78 1.76 1.75 1.74 1.72 1.69 1.68 1.66 1.06
0.00
0.92 0.97 3.04 1.88 1.05 1.00 2.33 19.61
f1 (ppm)

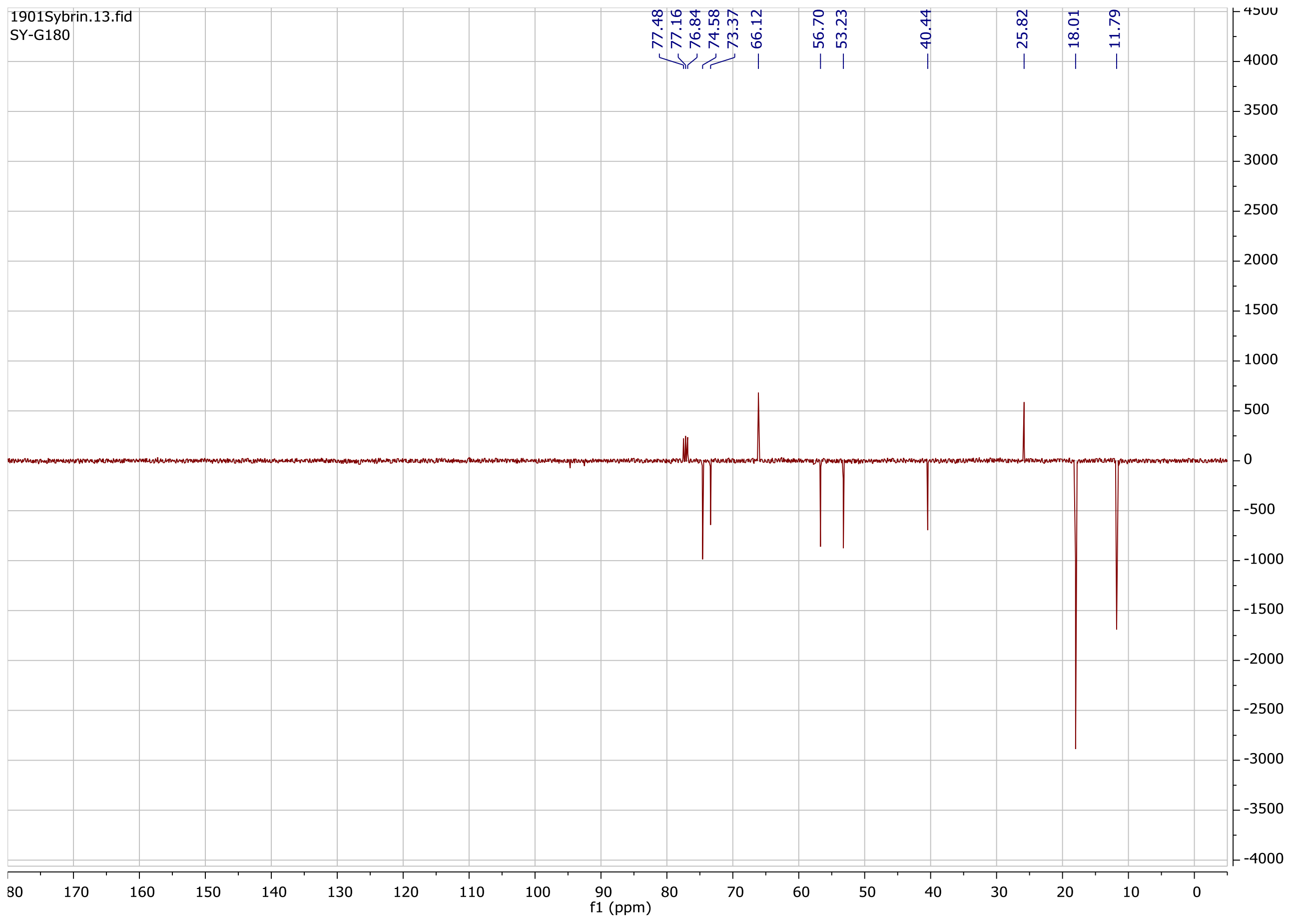
1901Sybrin.13.fid
SY-G180
77.48 77.16 76.84 74.58 73.37 66.12 56.70 53.23 40.44 25.82 18.01 11.79
f1 (ppm)

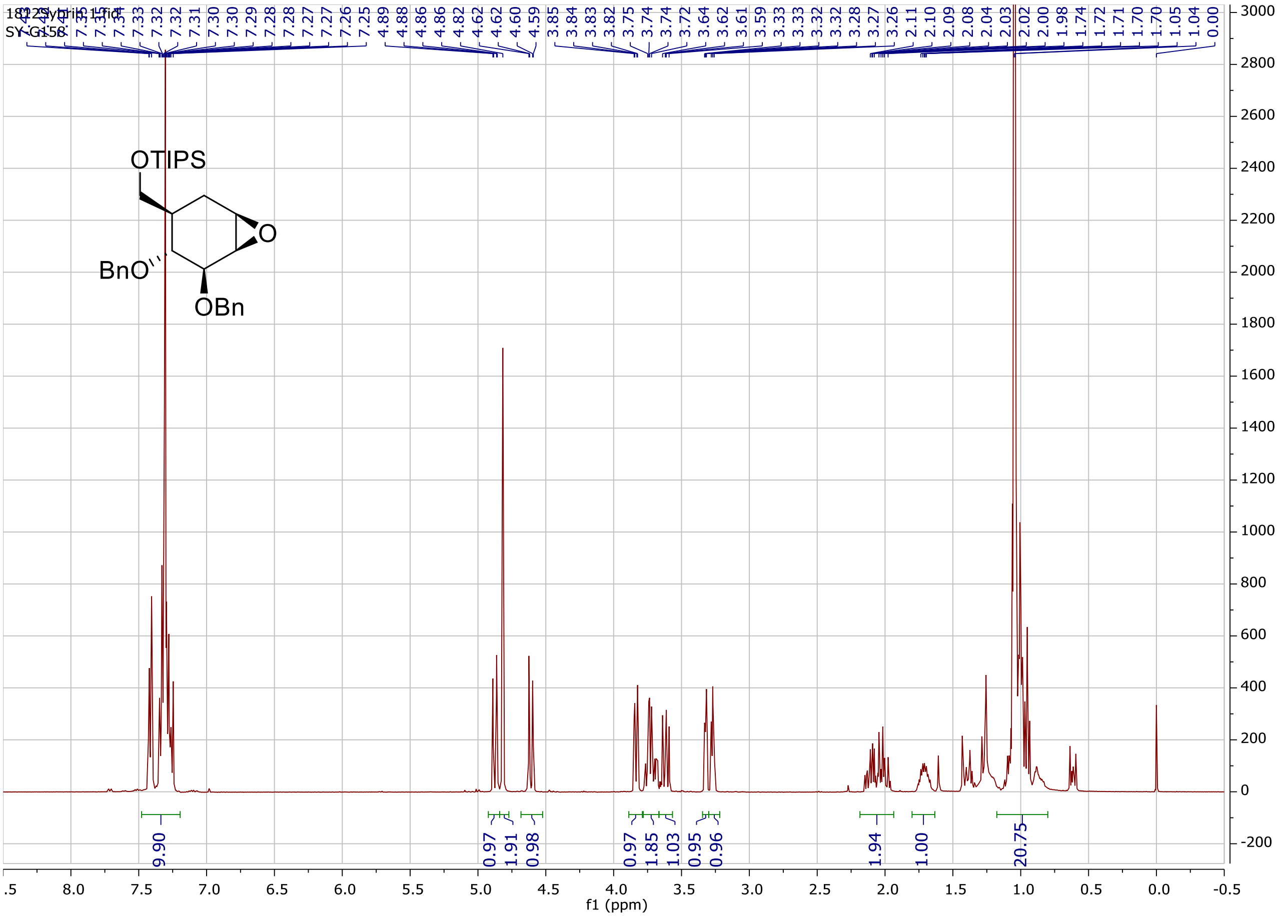
1812Sybrin.1.fid
SY-G158
OTIPS
BnO
OBn
O
f1 (ppm)

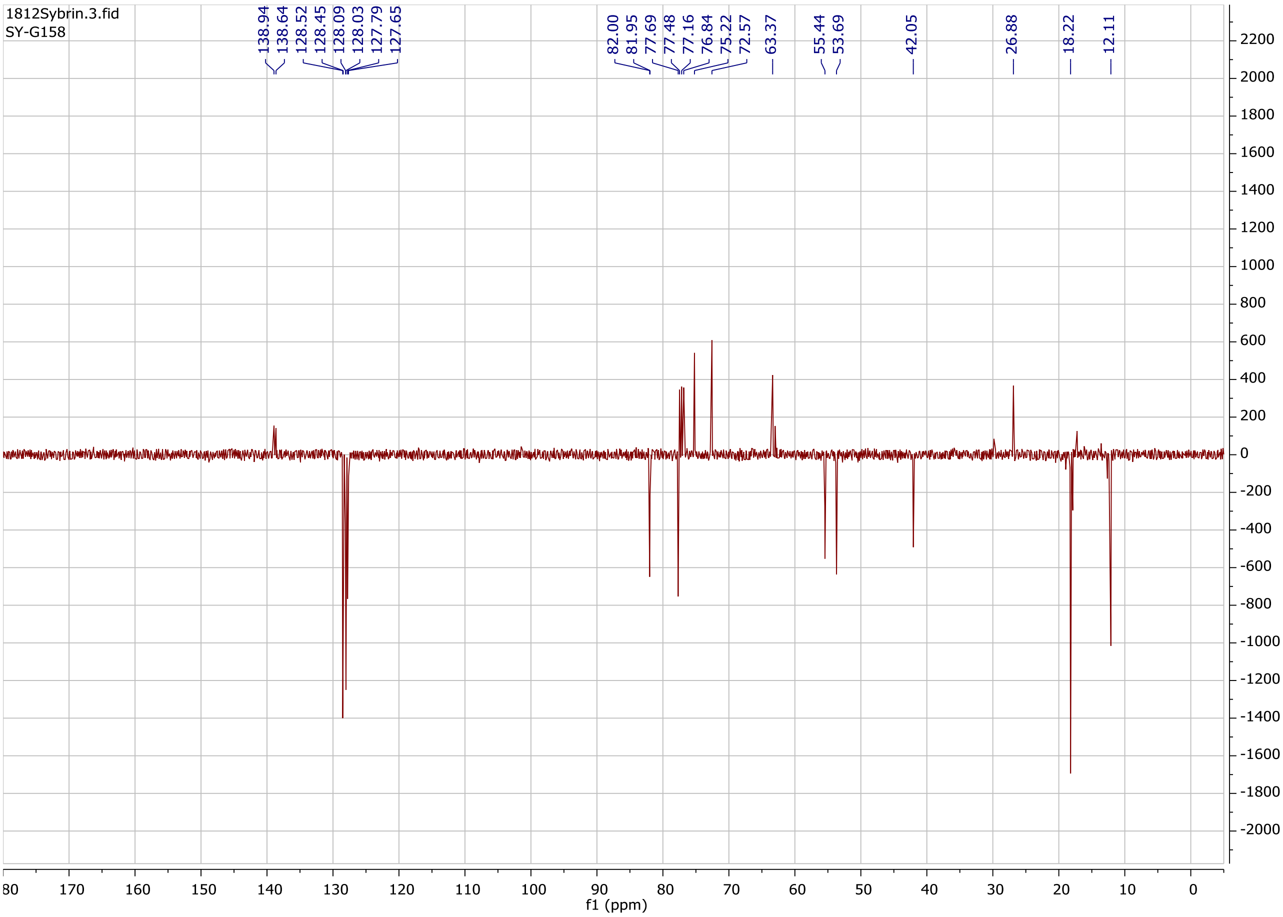
1812Sybrin.3.fid
SY-G158
138.94
138.64
128.52
128.45
128.09
128.03
127.79
127.65
82.00
81.95
77.69
77.48
77.16
76.84
75.22
72.57
63.37
55.44
53.69
42.05
26.88
18.22
12.11
f1 (ppm)

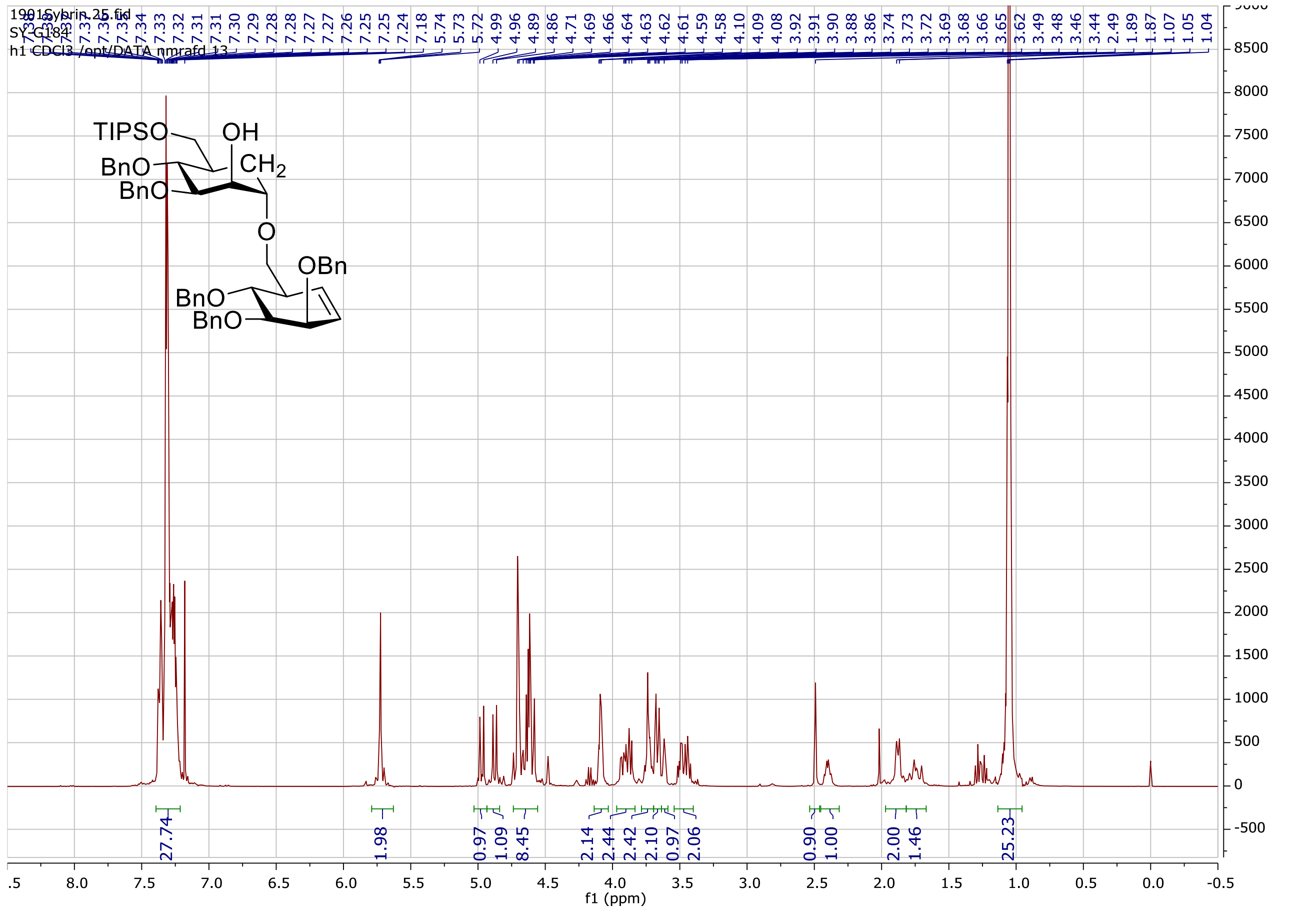

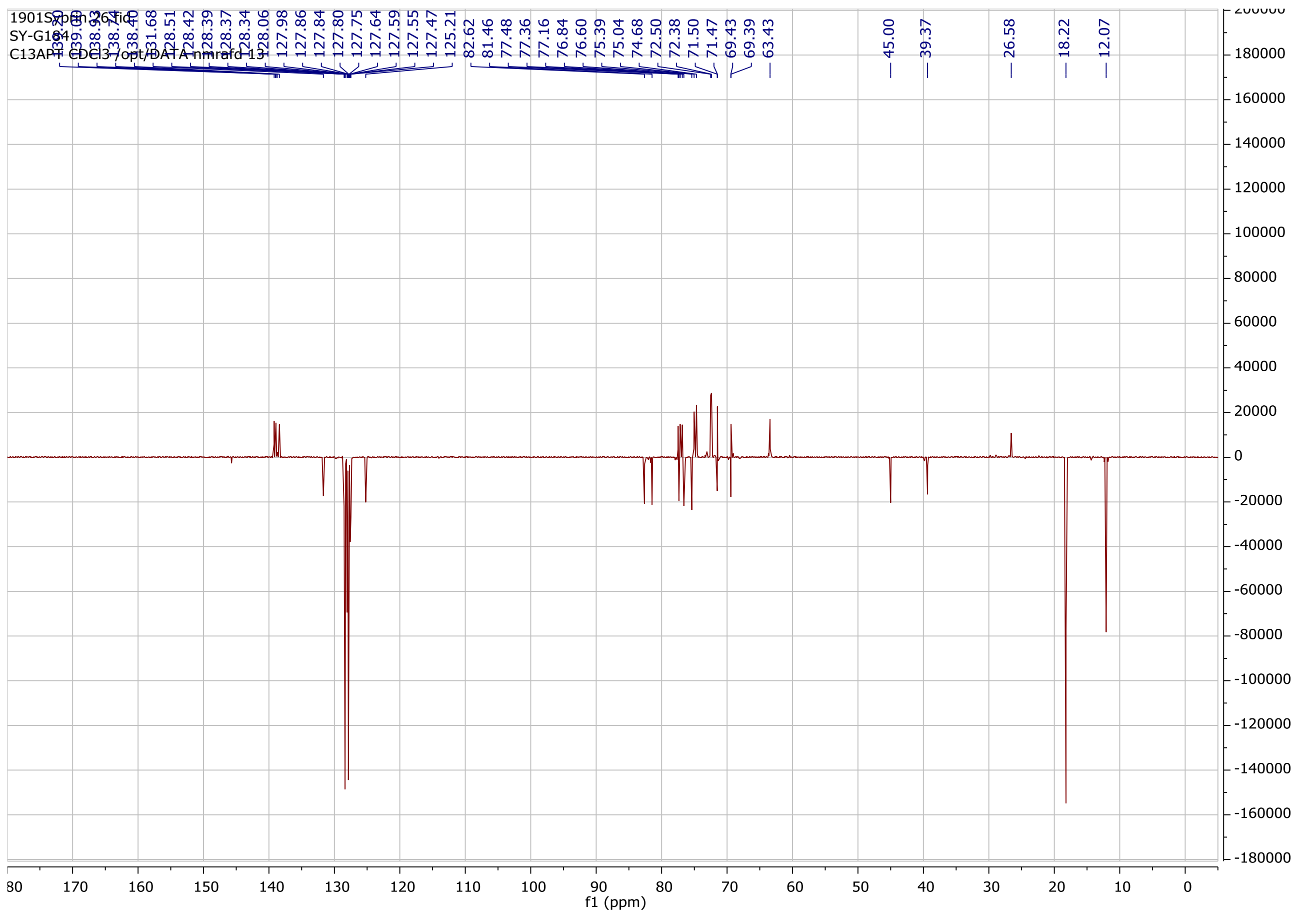

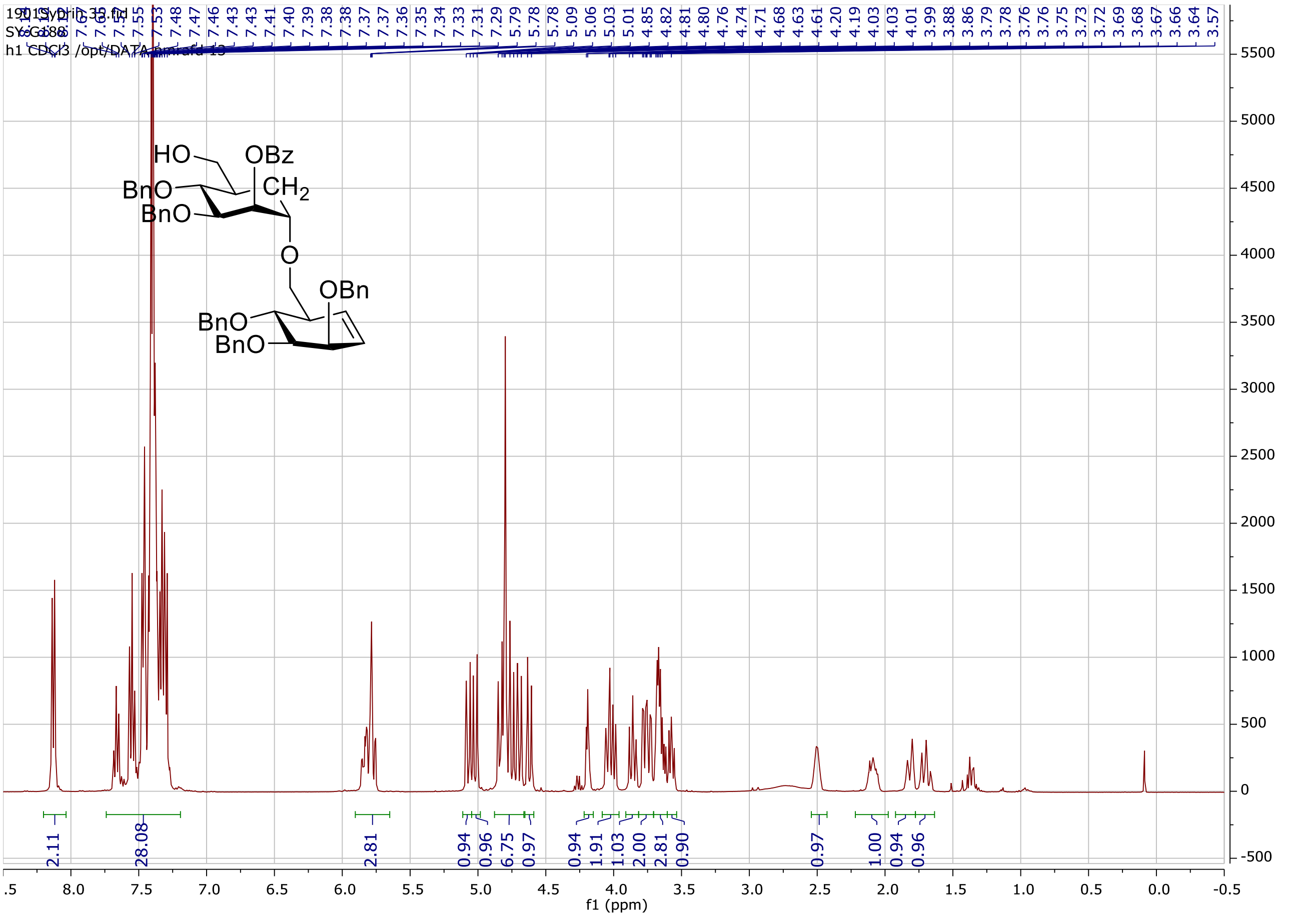
7.55 7.53 7.48 7.47 7.46 7.43 7.41 7.40 7.39 7.38 7.38 7.37 7.36 7.35 7.34 7.33 7.31 7.29 5.79 5.78 5.78 5.09 5.06 5.03 5.01 4.85 4.82 4.81 4.80 4.76 4.74 4.71 4.70 4.68 4.63 4.61 4.20 4.19 4.03 4.01 3.99 3.88 3.86 3.79 3.78 3.76 3.75 3.73 3.72 3.69 3.68 3.67 3.66 3.64 3.57
HO
OBz
BnO
BnO
CH2
O
OBn
BnO
BnO
2.11 28.08 2.81 0.94 0.96 6.75 0.97 0.94 1.91 1.03 2.00 2.81 0.90 0.97 1.00 0.94 0.96
f1 (ppm)

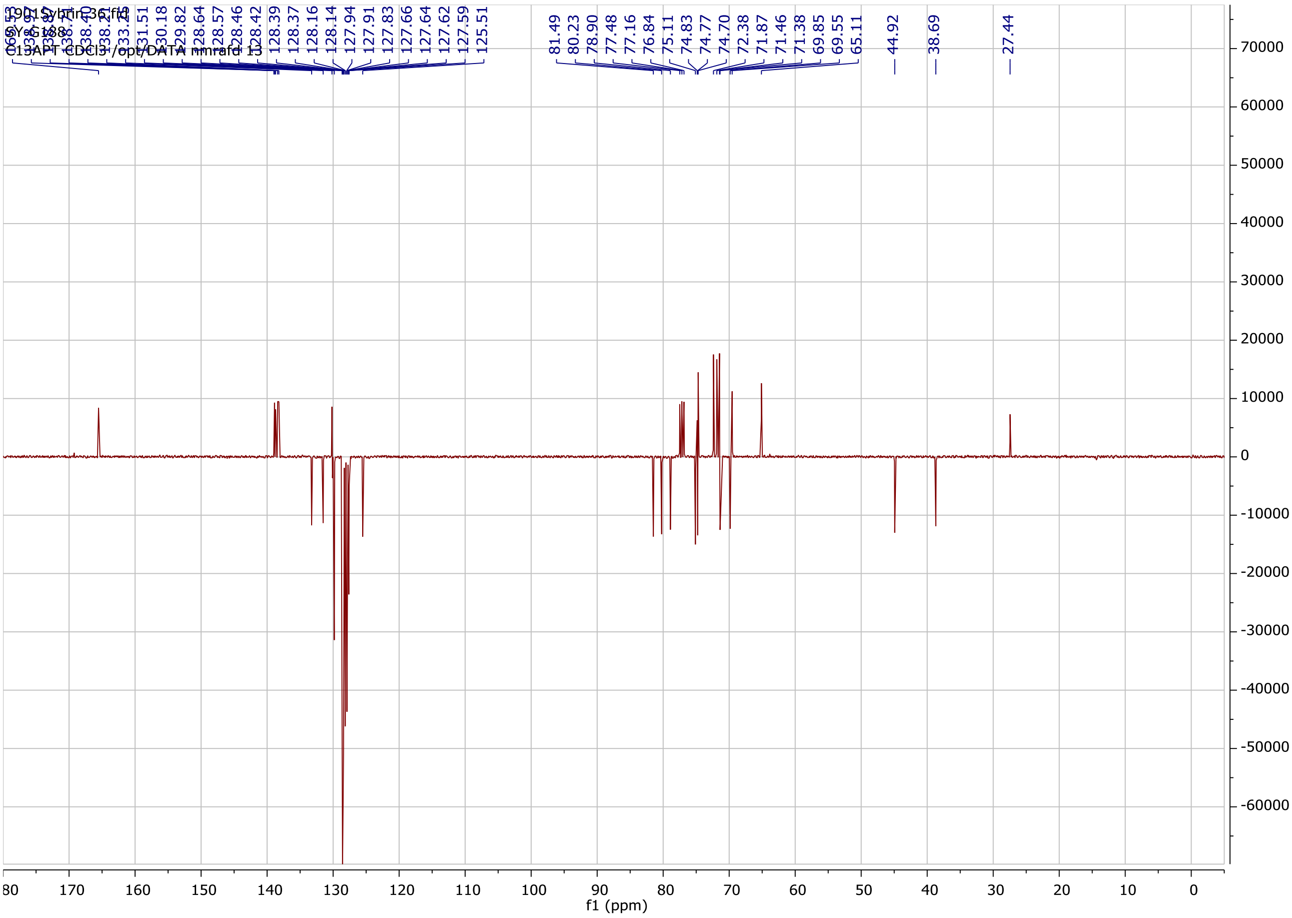
133.25 131.51 130.18 129.82 128.64 128.57 128.46 128.42 128.39 128.37 128.16 128.14 127.94 127.91 127.83 127.66 127.64 127.62 127.59 125.51
81.49 80.23 78.90 77.48 77.16 76.84 75.11 74.83 74.77 74.70 72.38 71.87 71.46 71.38 69.85 69.55 65.11
44.92 38.69 27.44
f1 (ppm)

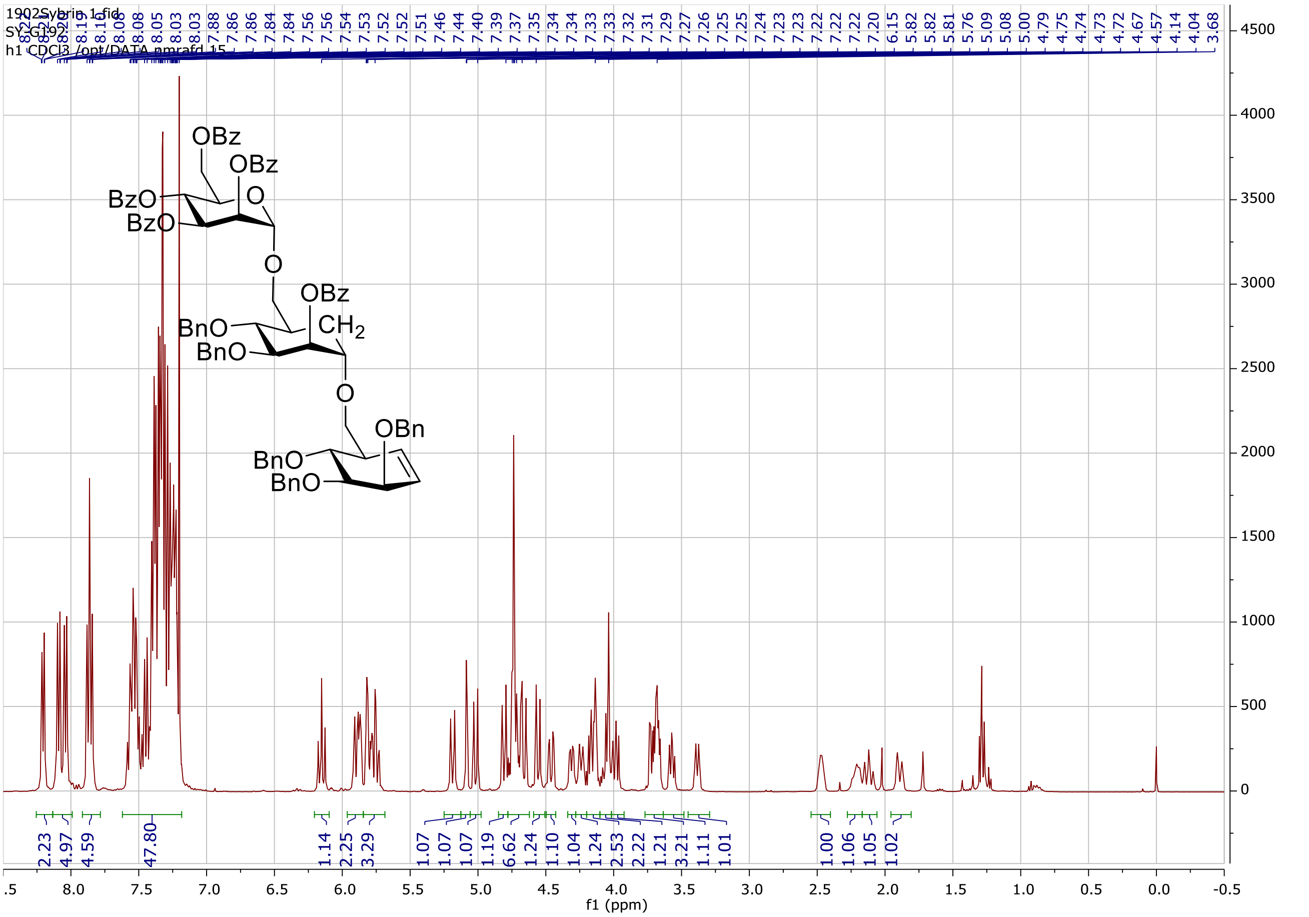

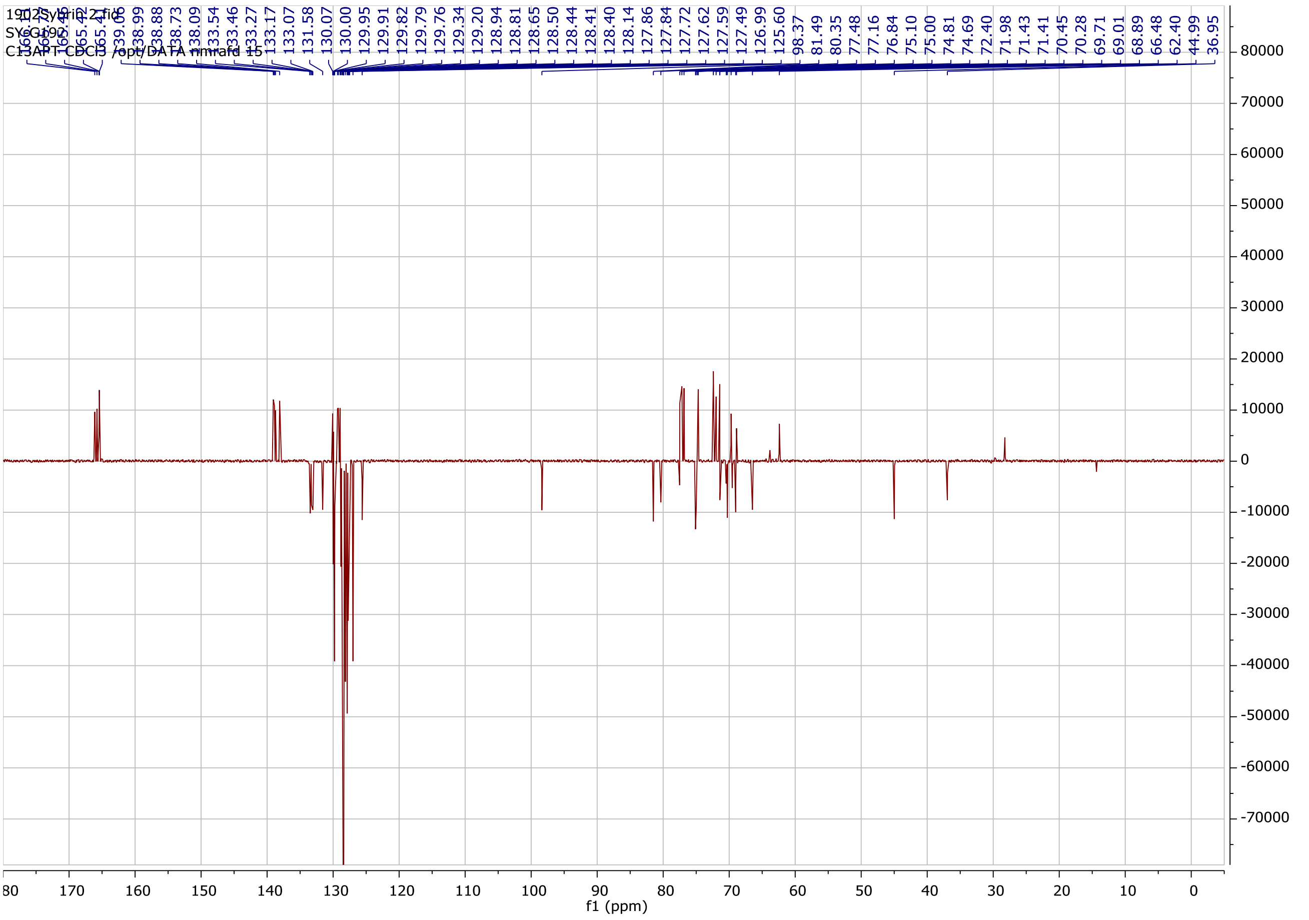

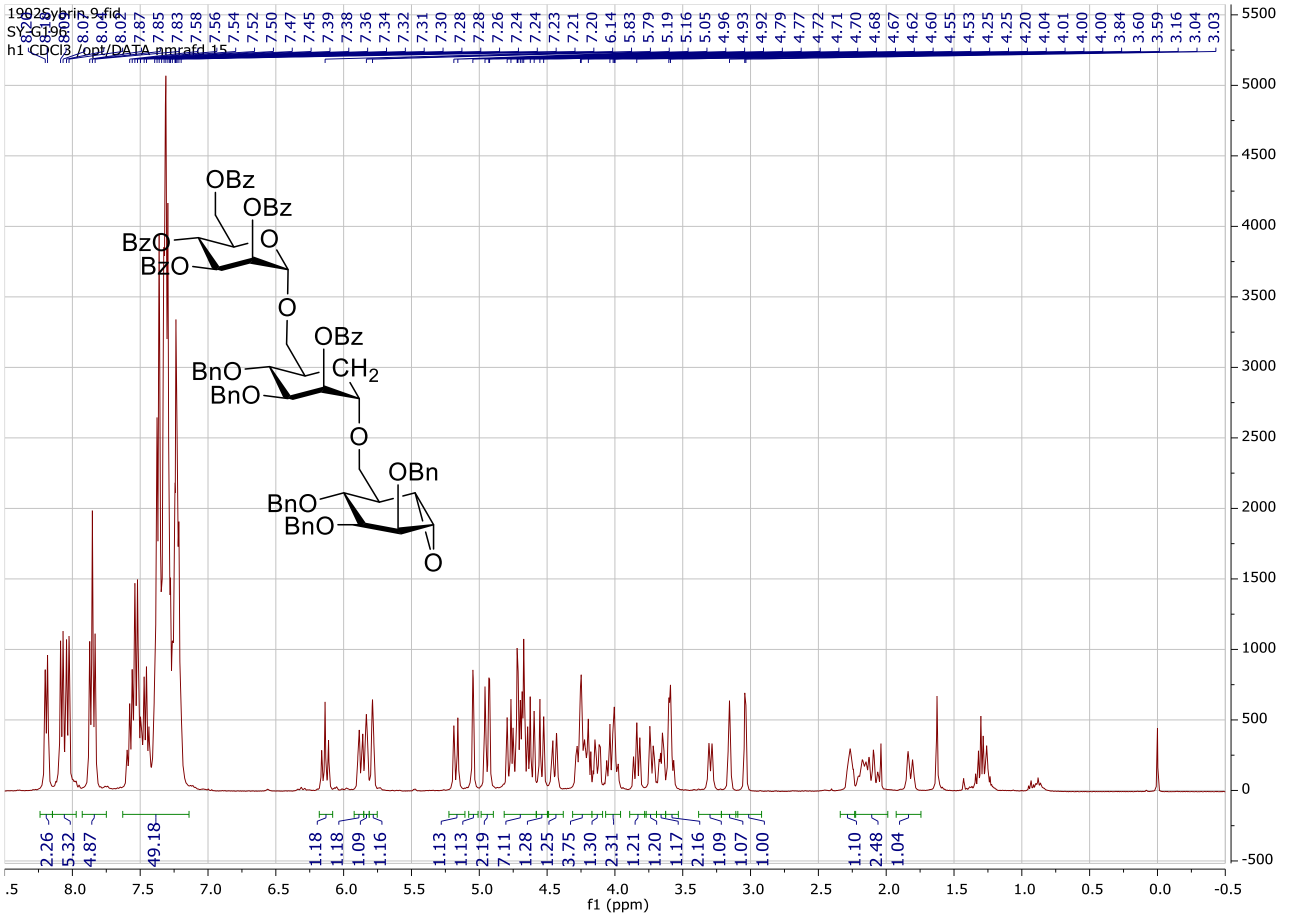
1902Sybrin.9.fid
SY-G196
h1 CDCl3 /opt/DATA nmrafd 15
8.04
8.02
7.87
7.85
7.83
7.58
7.56
7.54
7.52
7.50
7.47
7.45
7.39
7.38
7.36
7.34
7.32
7.31
7.30
7.28
7.26
7.24
7.24
7.23
7.21
7.20
6.14
5.83
5.79
5.19
5.16
5.05
4.96
4.93
4.92
4.79
4.77
4.72
4.71
4.70
4.68
4.67
4.62
4.60
4.55
4.53
4.25
4.25
4.20
4.04
4.01
4.00
4.00
3.84
3.60
3.59
3.16
3.04
3.03
OBz
OBz
BzO
BzO
O
O
OBz
BnO
BnO
CH2
O
OBn
BnO
BnO
O
2.26
5.32
4.87
49.18
1.18
1.18
1.09
1.16
1.13
1.13
2.19
7.11
1.28
1.25
3.75
1.30
2.31
1.21
1.20
1.17
2.16
1.09
1.07
1.00
1.10
2.48
1.04
f1 (ppm)

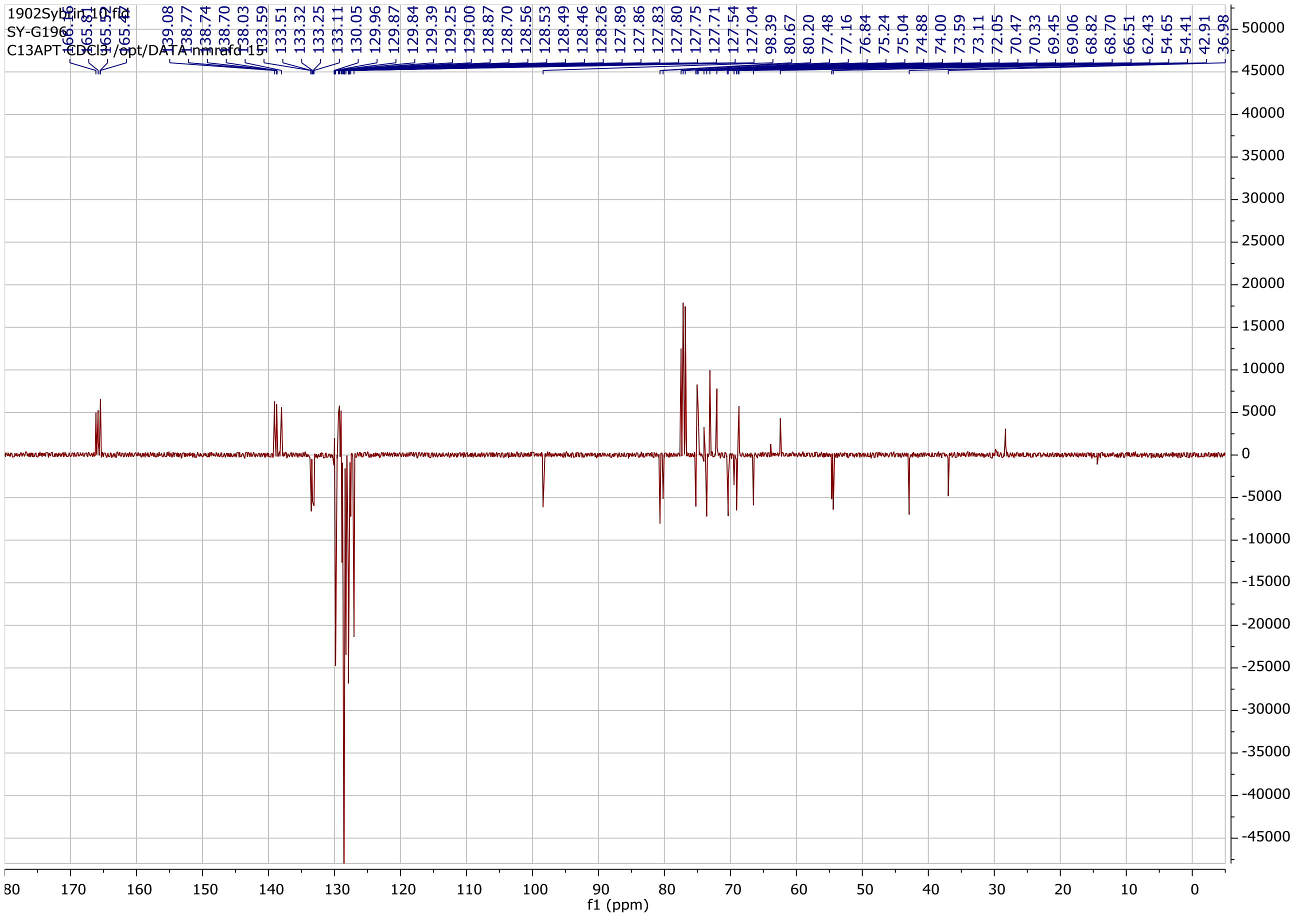
1902Sybrin.10.fid
SY-G196
C13APT CDCl3 /opt/DATA nmrafd 15
139.08
138.77
138.74
138.70
138.03
133.59
133.51
133.32
133.25
133.11
130.05
129.96
129.87
129.84
129.39
129.25
129.00
128.87
128.70
128.56
128.53
128.49
128.46
128.26
127.89
127.86
127.83
127.80
127.75
127.71
127.54
127.04
98.39
80.67
80.20
77.48
77.16
76.84
75.24
75.04
74.88
74.00
73.59
73.11
72.05
70.47
70.33
69.45
69.06
68.82
68.70
66.51
62.43
54.65
54.41
42.91
36.98
f1 (ppm)

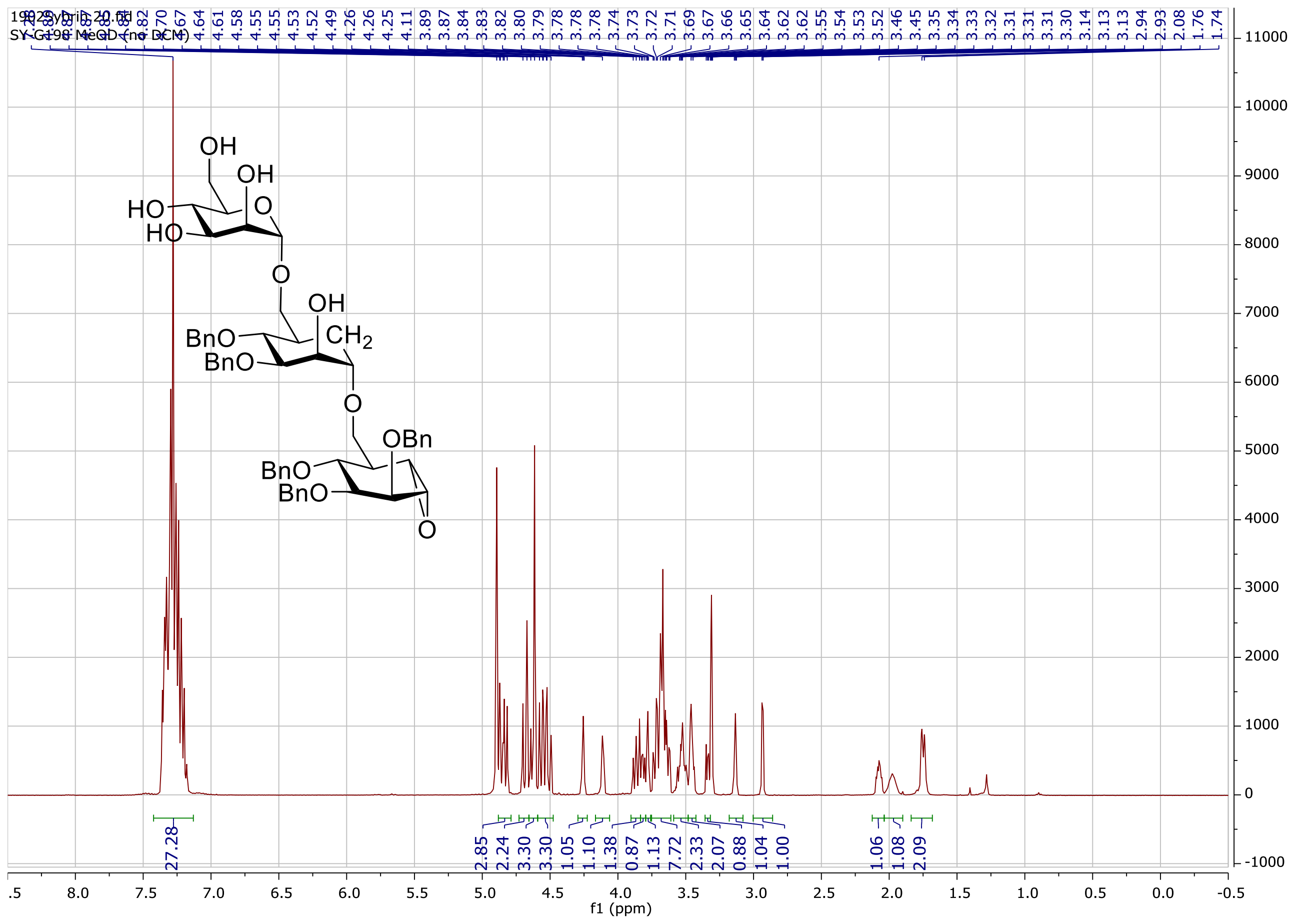
1902Sybrin.20.fid
SY-G198 MeOD (no DCM)
OH
OH
HO
HO
O
O
OH
CH2
BnO
BnO
O
O
OBn
BnO
BnO
O
f1 (ppm)

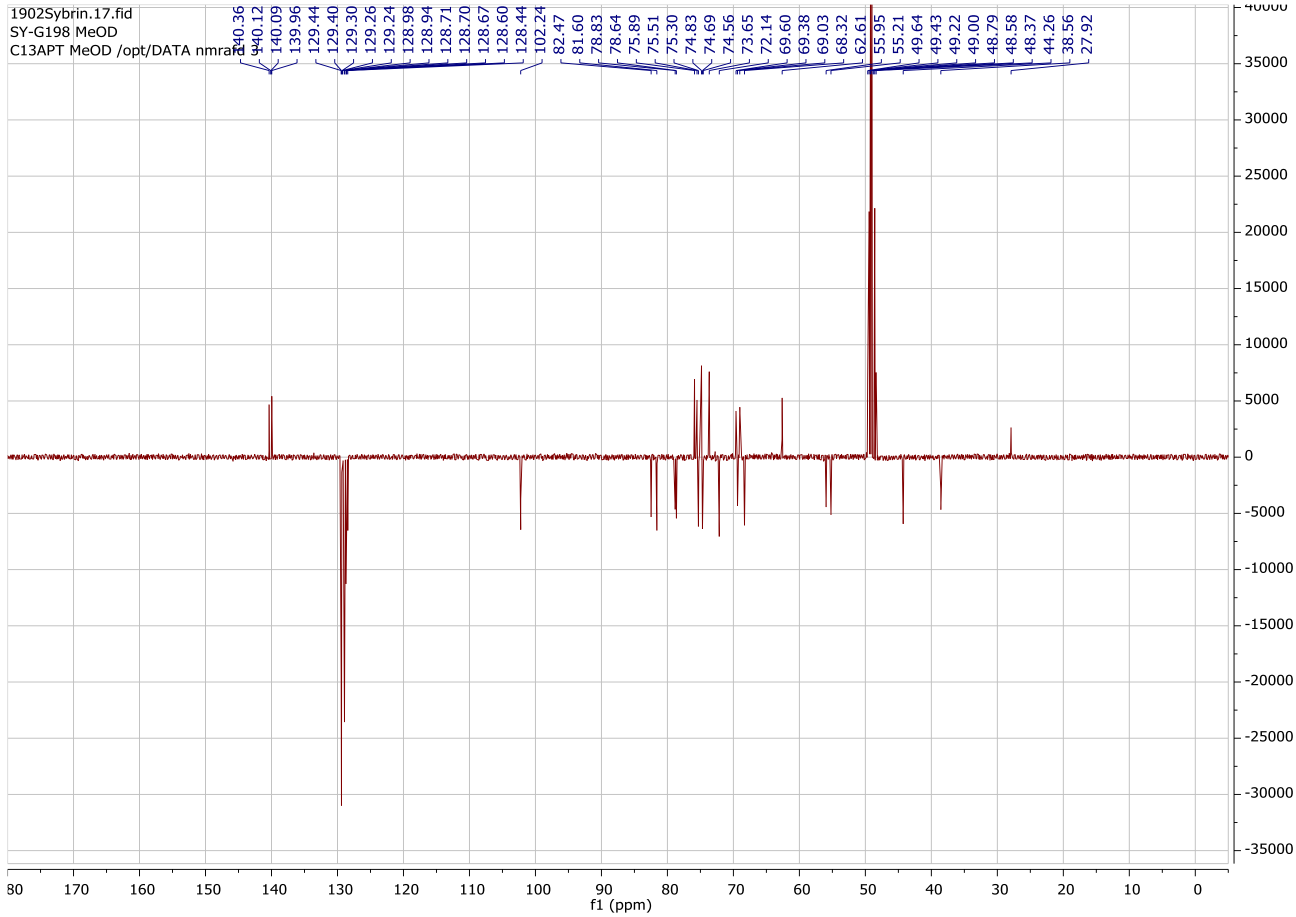
1902Sybrin.17.fid
SY-G198 MeOD
C13APT MeOD /opt/DATA nmrard
f1 (ppm)

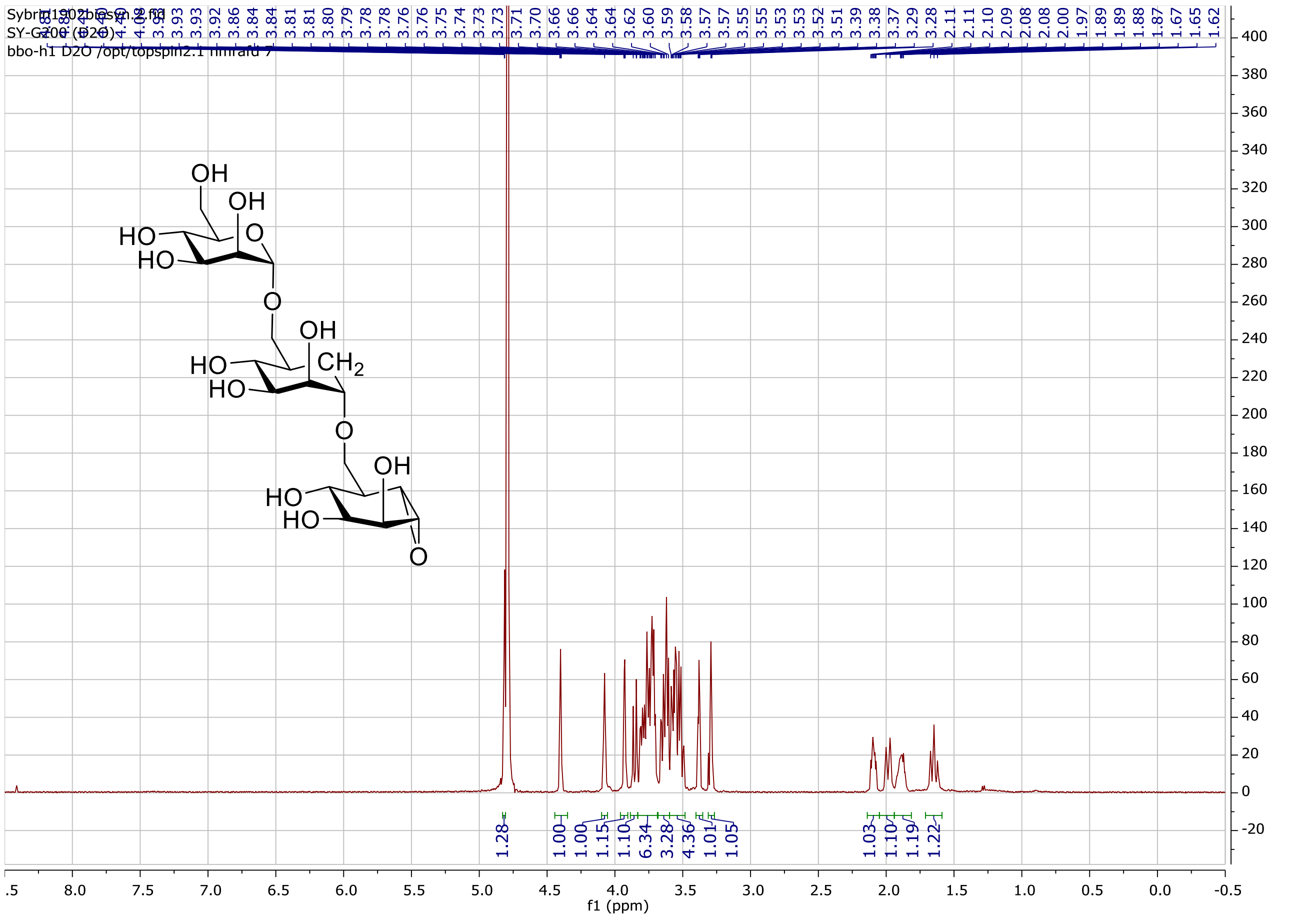
SY-G200 (D2O)
bbo-h1 D2O /opt/topspin2.1 nmrafd 7
f1 (ppm)

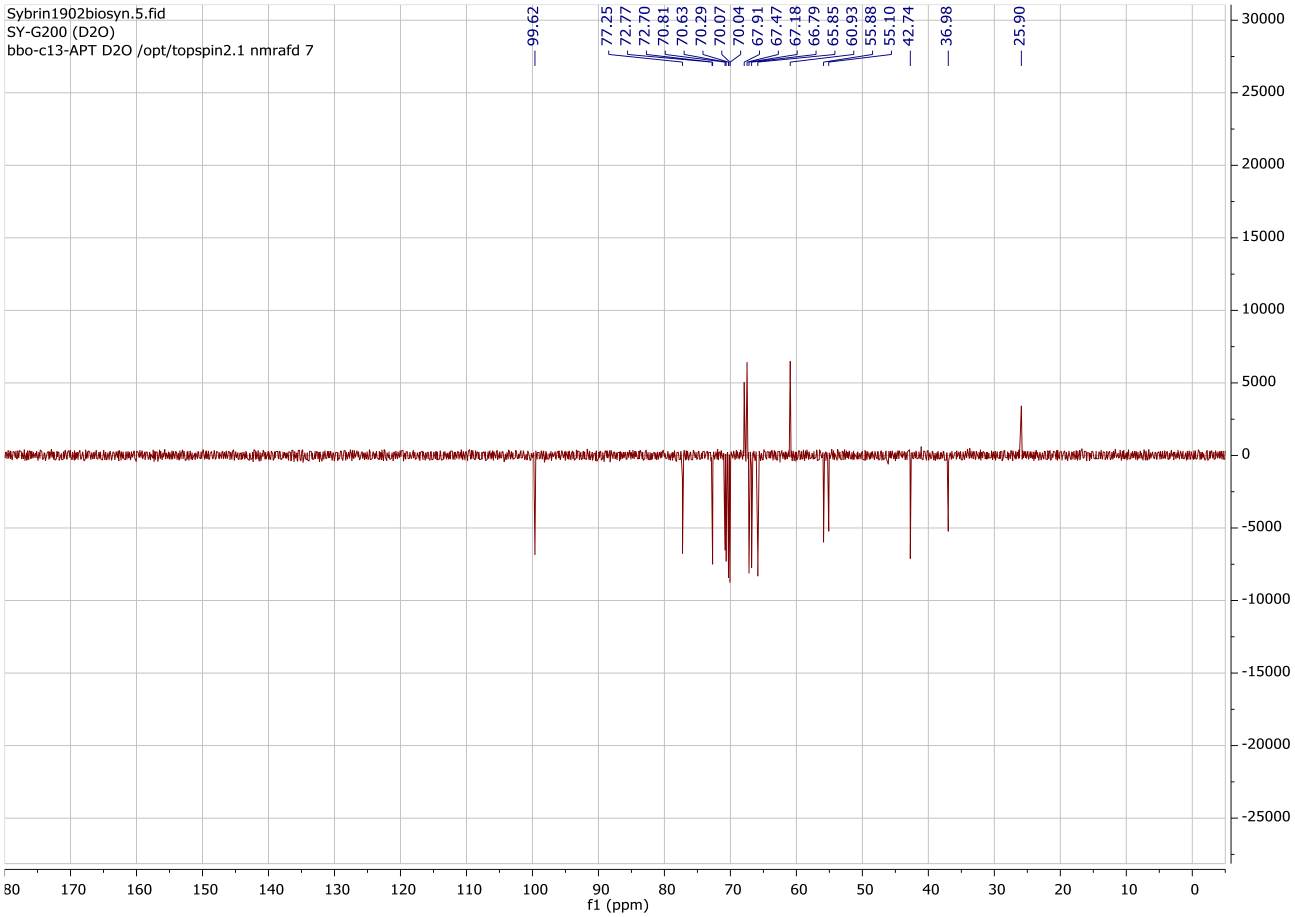
Sybrin1902biosyn.5.fid
SY-G200 (D2O)
bbo-c13-APT D2O /opt/topspin2.1 nmrafd 7
99.62
77.25
72.77
72.70
70.81
70.63
70.29
70.07
70.04
67.91
67.47
67.18
66.79
65.85
60.93
55.88
55.10
42.74
36.98
25.90
f1 (ppm)

5. References

[1] A. J. Thompson, G. Speciale, J. Iglesias-Fernandez, Z. Hakki, T. Belz, A. Cartmell, R. J. Spears, E. Chandler, M. J. Temple, J. Stepper, H. J. Gilbert, C. Rovira, S. J. Williams, G. J. Davies, *Angew. Chem. Int. Ed.* **2015**, *54*, 5378-5382; *Angew. Chem*. **2015**, *217*, 5468-5472.

[2] G. Winter, D. G. Waterman, J. M. Parkhurst, A. S. Brewster, R. J. Gildea, M. Gerstel, L. Fuentes-Montero, M. Vollmar, T. Michels-Clark, I. D. Young, N. K. Sauter, G. Evans, *Acta Crystallographica Section D* **2018**, *74*, 85-97.

[3] W. Kabsch, *Acta Crystallographica Section D* **2010**, *66*, 125-132.

[4] L. Potterton, J. Agirre, C. Ballard, K. Cowtan, E. Dodson, P. R. Evans, H. T. Jenkins, R. Keegan, E. Krissinel, K. Stevenson, A. Lebedev, S. J. McNicholas, R. A. Nicholls, M. Noble, N. S. Pannu, C. Roth, G. Sheldrick, P. Skubak, J. Turkenburg, V. Uski, F. von Delft, D. Waterman, K. Wilson, M. Winn, M. Wojdyr, *Acta Crystallographica Section D* **2018**, *74*, 68-84.

[5] A. Vagin, A. Teplyakov, *Acta Crystallogr D* **2010**, *66*, 22-25.

[6] G. N. Murshudov, P. Skubak, A. A. Lebedev, N. S. Pannu, R. A. Steiner, R. A. Nicholls, M. D. Winn, F. Long, A. A. Vagin, *Acta Crystallogr D* **2011**, *67*, 355-367.

[7] P. Emsley, B. Lohkamp, W. G. Scott, K. Cowtan, *Acta Crystallogr D* **2010**, *66*, 486-501.

[8] S. McNicholas, E. Potterton, K. S. Wilson, M. E. M. Noble, *Acta Crystallographica Section D* **2011**, *67*, 386-394.

[9] L. Konermann, E. Ahadi, A. D. Rodriguez, S. Vahidi, *Analytical Chemistry* **2013**, *85*, 2-9.

[10] Z. Armstrong, C. L. Kuo, D. Lahav, B. Liu, R. Johnson, T. J. M. Beenakker, C. de Boer, C. S. Wong, E. R. van Rijssel, M. F. Debets, B. I. Florea, C. Hissink, R. G. Boot, P. P. Geurink, H. Ovaa, M. van der Stelt, G. M. van der Marel, J. D. C. Codee, J. Aerts, L. Wu, H. S. Overkleeft, G. J. Davies, *J Am Chem Soc* **2020**, *142*, 13021-13029.

[11] B. Becker, R. H. Furneaux, F. Reck, O. A. Zubkov, *Carbohydr Res* **1999**, *315*, 148-158.

[12] H. Franzyk, M. Meldal, H. Paulsen, K. Bock, *Journal of the Chemical Society, Perkin Transactions 1* **1995**, 2883-2898.

[13] V. Bordoni, V. Porkolab, S. Sattin, M. Thépaut, I. Frau, L. Favero, P. Crotti, A. Bernardi, F. Fieschi, V. Di Bussolo, *RSC Advances* **2016**, *6*, 89578-89584.

[14] Q. Gao, C. Zaccaria, M. Tontini, L. Poletti, P. Costantino, L. Lay, *Organic & Biomolecular Chemistry* **2012**, *10*, 6673-6681.
